# Metabolome Profiling of Eight Chinese Yam (*Dioscorea polystachya* Turcz.) Varieties Reveals Metabolite Diversity and Variety Specific Uses

**DOI:** 10.3390/life11070687

**Published:** 2021-07-14

**Authors:** Xiaoxuan Zeng, Dahui Liu, Luqi Huang

**Affiliations:** 1 Faculty of Pharmacy, Hubei University of Chinese Medicine, Wuhan 430065, China; zengxx2021@126.com (X.Z.); liudahui@hbtcm.edu.cn (D.L.); 2 State Key Laboratory Breeding Base of Dao-di Herbs, National Resource Center for Chinese Materia Medica, China Academy of Chinese Medical Sciences, Beijing 100700, China

**Keywords:** amino acid biosynthesis in yam, Chinese yam, diosgenin, ESI-Q TRAP-MS/MS, riboflavin, *Dioscorea* spp., yam metabolite atlas

## Abstract

The Chinese yam (*Dioscorea polystachya* Turcz.) is an underutilized orphan tuber crop. However, in China it has been used in traditional medicine and food for centuries due to the presence of high starch, protein, fiber, and biologically active compounds. Knowledge on the metabolomic profiles of Chinese yam varieties is needed to explore the underutilized metabolites and variety specific uses. Here, the metabolome of eight Chinese yam varieties that are cultivated in different Chinese regions was profiled. A total of 431 metabolites belonging to different biochemical classes was detected. The majority of detected metabolites were classified as amino acids and derivatives. The different yam varieties offer unique uses; e.g., Hebei Ma Yam, Henan Huai Yam, and Henan Wild Yam were the most metabolically enriched and suitable as food and medicine. Yams from Hubei region had comparable nutritional profiles, which is most probably due to their geographical origin. Specifically, Henan Wild Yam had the highest concentrations of diosgenin, vitamins, and polysaccharides. Overall, this study presents a metabolome reference for *D. polystachya* varieties.

## 1. Introduction

Yam (*Dioscorea polystachya* Turcz.) is indigenous to China, Korea, Kuril Islands, and Taiwan. It has been introduced to United States of America, Japan, and Himalayas [1]. In China, it is commonly known as “Huai Shan Yao” and is cultivated for food and medical purposes [2]. Yam production has been doubled over the last two decades; in 2018, the global yam production reached 72.58 million tons (www.fao.org, accessed on 12 January 2021). China receives a major share (17%) of global yam production. Within China, it is mainly produced in Henan province, where different varieties/cultivars are grown [3]. The varieties/cultivars are distinguishable mainly due to the tuber shape e.g., cylindrical, flattened, round, etc. [4]. Nutritionally, yam tubers are rich in starch (65%), protein (9%), and fiber (1.2%) [5]. The percentage of these nutritional components depends on the cultivar. Yams have an increasing demand due to its preferred organoleptic and sensorial properties as compared to other carbohydrate sources. Moreover, yams have a better storage quality as compared to other crops, i.e., potato, cassava, and sweet potato [6].

Yam has been used in Traditional Chinese Medicine for the treatment of diarrhea, diabetes, and asthma [7]. The utility of yam tubers in medicine depends largely on the presence and contents of bioactive compounds and nutritional components [1,8]. The main ingredients with pharmacological activities include antioxidants, polysaccharides, sapogenins (diosgenin), and dioscorin [9,10]. Studies have also been conducted in other countries on the medicinal uses of yams e.g., Korean yam (*Dioscorea opposite*) showed the presence of phenolic compounds with pancreatic lipase inhibitory activity [11]. On the other hand, yams are used in animal food and feed, instant noodles, and drinks because of their high starch contents [7]. High polysaccharide contents in yams have made them a useful functional food and medicine source since it has been reported to improve insulin resistance and obesity with antidiabetic and antimicrobial effects [8,12,13]. These properties are linked with sapogenin contents, and according to an estimate, more than 500 million USD market share is because of its use as a source of diosgenin [14]. Together with polysaccharides, diosgenin has been used to formulate a wound-healing cream as well as an agent to reduce skin inflammation [15].

The abundance of various compounds, i.e., saponins as compared to *Dioscorea* species as well as other rhizome/tuber containing species, has long been known and is being continuously exploited ([16] and references therein). To further explore the abundance of nutritional and functional components, it is necessary to discover the metabolic profiles of yam tubers of different varieties. Discovery of existing and novel metabolites in different yam genotypes will add value to the existing information on the health beneficial compounds. Such information will also result in the improvement of varieties having new traits. Recent omics advances can be a useful tool to capture an overall comparative metabolite profile. In particular, the metabolomics can give a direct profile of metabolites of interest [17]. However, this promising avenue of yam research has not been investigated much. The metabolome profiles of different *Dioscorea* species were recently explored, which revealed that the diversity in this genus is underutilized and the species are rich in high value compounds [18]. Another study explored the diversity of nutritional and bioactive compounds in 25 cultivars (*Dioscorea* spp.) belonging to different Chinese regions. Both these studies helped to understand the metabolome profiles of different *Dioscorea* species and cultivars. Champagne et al. [19] profiled the metabolomes of tropical root crops, including *Dioscorea* species, and revealed a wealth of inter- and intra-species diversity. The authors emphasized that global metabolomics has potential for identification of compounds within species and is a much-needed avenue in the genus *Dioscorea* [19,20]. A literature survey demonstrated that different cultivars within a species have pronounced variations in terms of metabolomic profiles e.g., *Amaranthus gangeticus* [21,22,23], *A. hypochondriacus* [24] drought-tolerant *A. tricolor* [22,25,26], stem amaranth [27], *A. blitum* [28], green morph amaranth [29], weedy amaranth [30], and red morph amaranth [31]. Similarly, it has been reported that the secondary metabolite profiles of *Dioscorea* spp. differ [32]. A study on the *Dioscorea* species of India attempted to screen the ethnobotanical importance of root tubers and proposed that differences exist within species in terms of phytochemicals and their concentrations [33,34,35]. The secondary metabolite profiling of the *Dioscorea alata* L. also helped to clarify the traditional selection process of the grater yam cultivars [36]. Thus, attempts at the metabolite profiling of root or tuber crops are needed to facilitate modern breeding [37]. Considering the presence of intra-specific metabolite variability in root or tuber crops, we hypothesized that different cultivar of yams within same species of *D. polystachya* may have variations in metabolite profiles.

Different *D. polystachya* cultivars and varieties are grown in China, but the presence and diversity of metabolites have not been explored at metabolome level. Since, *D. polystachya* cultivars differ in tuber shape and size and different cultivars/varieties are preferred in different regions, it is necessary to fill the knowledge gap. Metabolomic profiles of these different genotypes will help to represent each variety based on the respective presence and concentration of metabolites. Furthermore, it will also aid in the identification of suitable cultivars for nutritional and/or medicinal uses. Here, we report and characterize the metabolome profiles of eight *D. polystachya* cultivars.

## 2. Materials and Methods

### 2.1. Plant Materials

**Figure 1:**
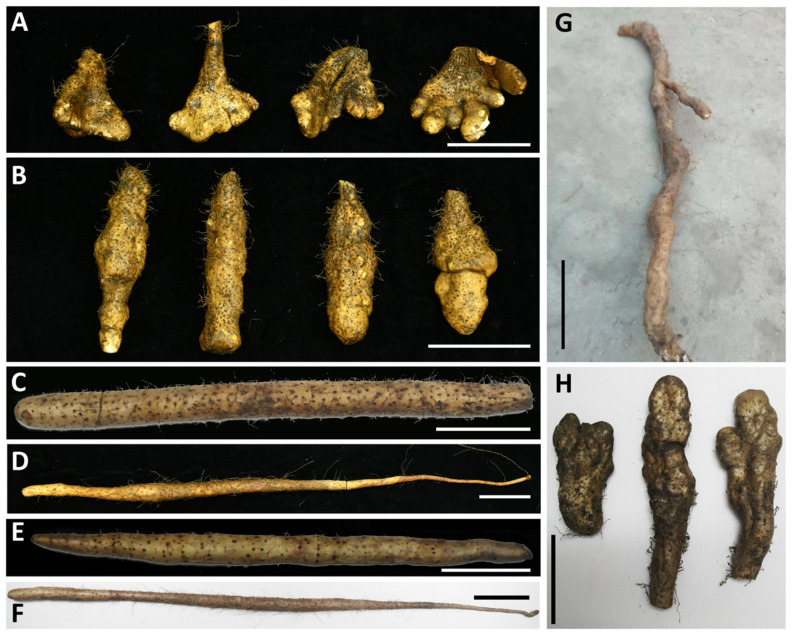
Tubers of different *Dioscorea polystachya* (yam) varieties. (**A**) Hubei Qichun Jiaozhang Yam (JZ), (**B**) Hubei Qichun Yam (QC), (**C**) Hebei Ma Yam (MS), (**D**) Shandong Tiegun Yam (ST), (**E**) Henan Huai Yam (HS), (**F**) Henan Tiegun Yam (TG), (**G**) Henan Wild Yam (WS), and (**H**) Jiangxi Ruichang Yam (RC). The bars are equal to 10 cm in each figure panel.

Eight yam varieties including Hebei Ma Yam (MS), Henan Huai Yam (HS), Henan Tiegun Yam (TG), Henan Wild Yam (WS), Hubei Qichun Jiaozhang Yam (JZ), Hubei Qichun Yam (QC), Jiangxi Ruichang Yam (RC), and Shandong Tiegun Yam (ST), that are cultivated in different regions of China, were chosen for this study (Figure 1). TG, QC, and RC are domestic varieties that are approved for local cultivation, while WS naturally grows in Henan Shenlong Mountains. The remaining samples are local cultivars. The yam varieties were selected based on their diverse morphology, i.e., tuber size and shape as well as preference by consumers in different Chinese regions. Moreover, these varieties cover the main yam producing areas. Each sample was collected from its respective producing area during post-frost harvest period in 2020 and identified as *D. polystachya* by Professor Dahui Liu. Three replicates of the yam tubers were collected, washed thoroughly with tap water and then with distilled water. Tubers were then dried and stored at −80 °C.

### 2.2. Sample Preparation and Extraction

Three technical replicates for each variety were freeze-dried in vacuum. The samples were then crushed in a mixer mill (MM 400, Retsch). The crushed powdered lyophilized sample (100 mg) was dissolved in 1.2 mL MeOH (70%) and mixed six times using a vortex for 30 s each. The mixture was then refrigerated at 4 °C until next morning and then centrifuged at 12,000× *g* for 10 min. Finally, extracts were filtered by using 0.22 µm filter and processed for UPLC-MS/MS analysis.

### 2.3. UPLC Conditions and ESI-Q TRAP-MS/MS

The extracted samples were then analyzed in UPLC-ESI-MS/MS system (Applied Biosystems 4500 Q TRAP). The UPLC-ESI-MS/MS conditions included Agilent SB-C18 1.8 µm, 2.1 mm × 100 mm column, and the mobile phase consisting of solvent A, pure water with 0.1% formic acid, and solvent B, acetonitrile with 0.1% formic acid. The measurements of the samples were taken by following a gradient program given below.

The starting conditions were 95% A and 5% B. Within 9 min, a linear gradient was programmed and a composition of 5% A and 95% B was maintained for 5 min. Next, a composition of 95% A, 5% B was adjusted within 1.10 and maintained for 2.9 min. The other settings included an injection volume of 4 µL, the flow velocity was set to 0.35 mL per minute, and the oven was set to 40 °C. The effluent was alternatively connected to an ESI-triple quadrupole-linear ion trap (QTRAP)-MS. A LIT and triple quadrupole-linear ion trap mass spectrometer was used to acquire QQQ scans on a triple quadrupole-linear ion trap mass spectrometer (Q TRPA), AB4500 Q TRAP UPLC-MS/MS System. The mass spectrometer was equipped with ESI turbo ion-spray interface and was operated in both positive and negative modes. It was controlled by Analyst 1.6.3 software (AB Sciex). The parameters that were used for ESI source operation, instrument tuning, and mass calibration were followed as reported earlier [38]. Briefly, for ion source and turbo spray, source temperature was 550 °C and the ion spray voltage was 5500 V (+ve) and/or −4500 V (−ve). The ion source gas I, gas II, III, and curtain gas were set at 50, 60, and 25.0 psi, respectively. The collision-activated dissociation was high. The instrument tuning and mass calibration were performed with 10 and 100 µmol/L polypropylene glycol solution in QQQ and LIT modes, respectively. QQQ scans were acquired as MRM experiments with collision gas (nitrogen) set to medium. DP and CE for individual MRM transitions were conducted with further DP and CE optimization. A specific set of MRM transitions were monitored for each period according to the metabolites eluted within this period.

Quality control samples were prepared by mixing sample extracts to analyze the repeatability of samples under the same processing method. In the process of instrumental analysis, a quality control sample was inserted into every 10 test analysis samples to monitor the repeatability of the analysis process.

The substances were qualitatively determined according to the secondary spectrum information based on a self-built database. The isotope signals and repetitive signals containing K+, Na+, and NH4+ ions were removed. The metabolite quantification was performed using multiple reaction monitoring (MRM) mode analysis of QQQ MS. In this mode, the quadrupole screens the precursor ions of the target substance, eliminates the ions corresponding to other molecular weight substances to preliminarily eliminate interference; the precursor ions are induced by the collision cell to be ionized and then fragmented to form many fragment ions. Following this, QQQ filter selects the required characteristics fragment ion, eliminates the interference of non-target ions in order to accurately quantify the substance and ensure good repeatability. Once the spectrum data of the metabolites are achieved, the mass spectrum peaks of the metabolites are integrated, followed by correction of the mass spectrum peaks of the same metabolites in different samples [39].

### 2.4. Data Analyses

Unsupervised principal component analysis (PCA) was performed in prcomp in R. Hierarchical cluster analysis (HCA) of normalized signal intensities and Pearson correlation coefficients (PCC) between the varieties/cultivars/WS as well as between their replicates was computed in R and presented as heatmaps. Orthogonal Projections to Latent Structures Discriminant Analysis (OPLS-DA) was performed MetaboAnalystR (in R) and VIP values were extracted. Log2 foldchange values were calculated and means were centered before OPLS-DA. Furthermore, a permutation test was performed. Finally, we determined the significantly differentially accumulated metabolites between the yam varieties based on variable importance in projection (VIP) ≥ 1 and absolute Log2FC (fold change) ≥1.

The identified metabolites were annotated in KEGG compound database (http://www.kegg.jp/kegg/compound/, accessed on 12 January 2021) followed by mapping on KEGG pathways (http://www.kegg.jp/kegg/pathway.html, accessed on 12 January 2021). The pathways in which the metabolites were significantly enriched, were processed in metabolites sets enrichment analysis (MSEA) and their significance was determined by *p*-values of the hypergeometric test. To study the change trend of the relative content of metabolites in different varieties, the average value of the relative content of different metabolites in each variety were standardized by z-score, and then k-means clustering analysis was performed [40].

## 3. Results

### 3.1. Metabolome Analysis

**Figure 2:**
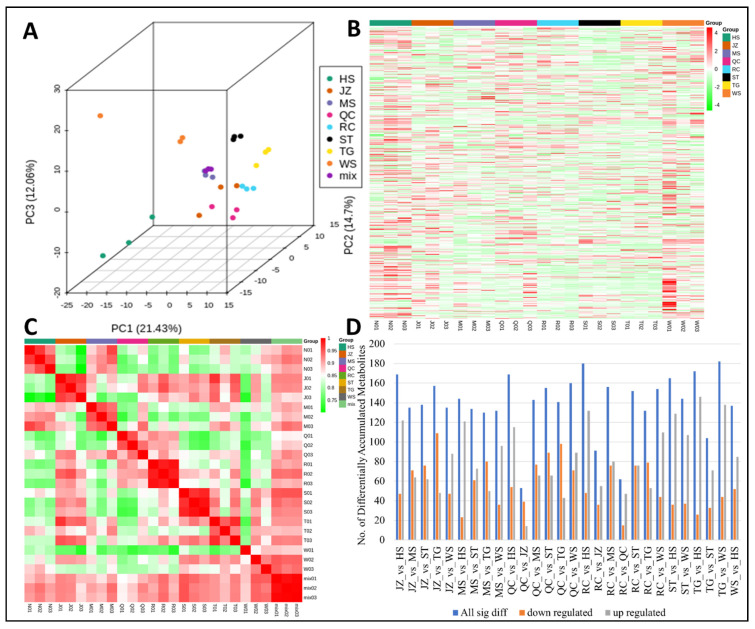
(**A**) Principal component analysis, (**B**) heatmap of metabolite concentrations, (**C**) Pearson correlation coefficients between metabolite profiles of replicates and treatments, and (**D**) summary of differentially accumulated metabolites between different Chinese Yam varieties. The variety names are Hubei Qichun Jiaozhang Yam (JZ), Hubei Qichun Yam (QC), Hebei Ma Yam (MS), Shandong Tiegun Yam (ST), Henan Huai Yam (HS), Henan Tiegun Yam (TG), Henan Wild Yam (WS), and Jiangxi Ruichang Yam (RC).

Profiling of the yam tuber extracts through UPLC-MS/MS analysis resulted in the detection of 431 metabolites (Supplementary Figure S1). Clustering metabolites in PCA highlighted the robustness of the analysis and represented 48.19% variation divided into three components (Figure 2A; Supplementary Table S1). ST and TG replicates were grouped in PCA, whereas HS and WS replicates were distributed close to ST and TG but not strictly clustered together. Similarly, RC, JZ, and QC replicates clustered together. Sample-wise accumulation of the detected metabolites is represented below as a heatmap (Figure 2B; Supplementary Table S1). Overall, the Pearson correlation coefficient between sample replicates was higher than 0.75 (Figure 2C; Supplementary Table S1), indicating a linear response in the detected metabolites. Overall, we observed 53–182 metabolites were differentially accumulated between different varieties (Figure 2D; Supplementary Table S1).

### 3.2. Yam Metabolite Atlas

**Figure 3:**
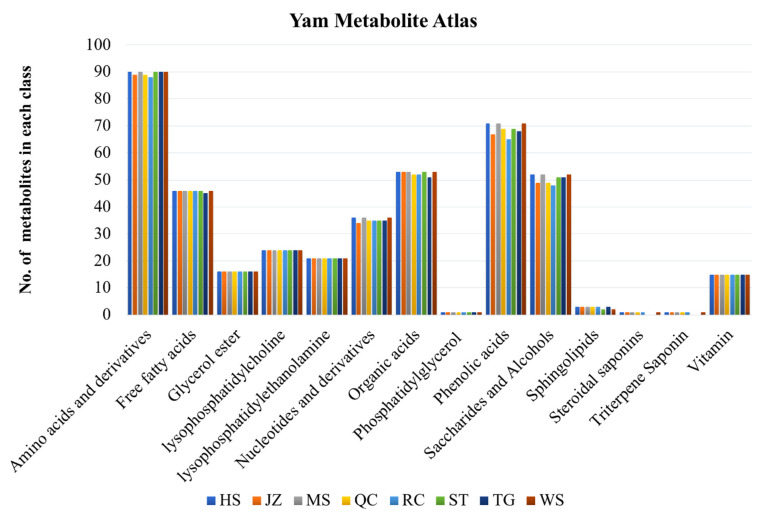
Metabolite count on compound-class basis in eight yam varieties; Hebei Ma Yam (MS), Henan Huai Yam (HS), Henan Tiegun Yam (TG), Henan Wild Yam (WS), Hubei Qichun Jiaozhang Yam (JZ), Hubei Qichun Yam (QC), Jiangxi Ruichang Yam (RC), and Shandong Tiegun Yam (ST).

The highest number of all the metabolites was the amino acids and derivatives followed by phenolic acids, organic acids, saccharides and alcohols, free fatty acids, nucleotides and derivatives, lysophosphatidylcholine, lysophosphatidylethanolamine, glycerol esters, vitamins, sphingolipids, phosphatidyl glycerol, saponins, and triterpenoid saponins (Figure 3). Overall, there was little or no variation among varieties in the number of detected metabolites in each class. Among the top-10 accumulated metabolites in each yam varieties, we found palmitaldehyde (a free fatty acid) was the most accumulated metabolite (Supplementary Table S2). Additionally, L-glutamic acid was also one of the top-10 most accumulated metabolites in all varieties. The least-accumulated metabolites were 12-oxo-phytodienoic acid (HS), cyclic 3′,5′-adenylic acid (JZ and TG), cyclo(Tyr-Leu) (MS and ST), 6-*O*-caffeoylarbutin (QC), LysoPE 15:1(2n isomer) (RC), and 5-*O*-caffeoylshikimic acid (WS). 12-oxo-Phytodienoic acid was detected in all varieties, however, its content was different in each variety. Three varieties i.e., HS, JZ, and WS had riboflavin (vitamin B2) in the least-10 accumulated metabolites, while in other varieties, its content was relatively higher. The HS and WS had D-fructose in top-10 accumulated metabolites. In addition to D-fructose, WS also had D-galactose and D-mannose, suggesting rich sugar contents in WS.

**Figure 4:**
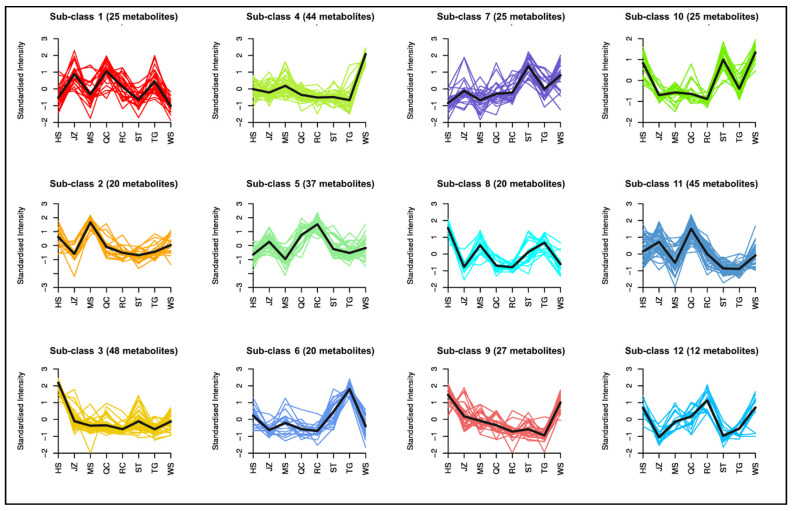
K-means diagram of the differentially accumulated metabolites between eight yam varieties. The abscissa represents the name of the sample, the ordinate represents the relative content of standardized metabolites, sub-class represents the number of the metabolite category with the same changing trend, and metabolite represents the number of metabolites in the category (metabolites within each sub-class are given in Supplementary Table S2). The eight yam varieties are Hebei Ma Yam (MS), Henan Huai Yam (HS), Henan Tiegun Yam (TG), Henan Wild Yam (WS), Hubei Qichun Jiaozhang Yam (JZ), Hubei Qichun Yam (QC), Jiangxi Ruichang Yam (RC), and Shandong TiegunYam (ST).

K-means clustering analysis revealed that the metabolites were detected in 12 clusters (Figure 4). The metabolites in sub-class 1 showed higher accumulation levels in JZ, QC, and TG. These metabolites were mainly classified as amino acids and derivatives (L-cysteine, N-acetyl-L-glutamic acid, N-acetyl-L-arginine, and oxiglutathione), nucleotides and derivatives, and phenolic acids (notably, p-coumaric acid-4-*O*-glucoside, 1-*O*-feruloyl-D-glucose, and vanillin). Two metabolites classified as amino acids (dehydroascorbic acid and pyridoxine-5′-*O*-glucoside) were also included in sub-class 1 metabolites. These observations suggest that the three varieties (JZ, QC, and TG) can be regarded as a rich source of the said metabolites as compared to the other varieties. The standard intensity of the 20 metabolites included in sub-class 2 was less than 1 in all varieties except for MS. Notably, these metabolites included L-alanine, fumaric acid, p-coumaric acid, and coniferyl alcohol, and methyldopa. Thus, MS could be an appropriate breeding material as well as a rich source of these metabolites as compared to the rest of the varieties. Forty-eight metabolites showed higher standard intensity in HS variety; these metabolites belonged to almost all classes mainly amino acids and derivatives, nucleotides and derivatives, and lipids. A higher lipid profile of HS indicates its suitability for high fat powder food production [9]. Similarly, K-means analysis indicated the higher content of sub-classes 4, 5, 6, 7, 8, 9, 10, 11, and 12 in WS, RC, TG, ST, HS, HS and WS, HS, TG, and WS, QC, and HS, QC, and WS. From these analyses, a relatively better metabolite profile of HS and WS suggests them as a superior source of the detected metabolites (Figure 4).

### 3.3. Variety Demarcation and Specific Metabolites

Overall, the number of detected metabolites were almost same in HS (430), MS (430), and WS (429). Similarly, JZ (420) and TG (420) and QC (422) and ST (423) showed almost same numbers of detected metabolites. The least number of metabolites were detected in RC (416) (Supplementary Table S1). Specially, we noticed that eleven (JZ), one (MS), nine (QC), fifteen (RC), eight (ST), eleven (TG), and two (WS) metabolites were not detected in respective varieties (Supplementary Table S1). These metabolites belonged to different compound classes. For example, the ones not detected in WS were dihydrosphingosine and 4-aminobenzoic acid. On the other hand, seven of the sixteen metabolites absent in RC were phenolic acids. These observations suggest that based on the differences in metabolite profile of each tested variety, their respective use can be specified and further pathways could be explored. When we looked at the individual metabolites, we found that 3-*O*-feruloylquinic acid was not detected in JZ, MS, QC, and RC. Seven metabolites (3-*O*-feruloylquinic acid, L-glutaminyl-L-valyl-L-valyl-L-cysteine, chlorogenic acid methyl ester, D-glucono-1,5-lactone, 5-*O*-p-coumaroylquinic acid, stachyose, and D(+)-melezitose O-rhamnoside) were detected in other varieties except JZ, QC, RC, suggesting relatively lower phenolic acid and saccharide content in these three varieties.

We also considered the fact that some of the varieties belonged to same geographical areas but different producing areas e.g., we had three varieties from Henan (HS, TG, and WS) and two from Hubei (JZ and QC). First of all, the varieties from the same geographical region did not group closely in the PCA plot e.g., HS, TG, and WS were located apart from each other in the PCA plot. Contrarily, the varieties from Hubei (JZ and QC) were grouped closely. This might indicate that the secondary metabolite profiles of these two are similar. To this regard, we found many similarities, e.g., both varieties lacked L-glutaminyl-L-valyl-L-valyl-L-cysteine, 3-*O*-feruloylquinic acid, 5-*O*-p-coumaroylquinic acid, D-glucono-1,5-lactone, stachyose, and D (+)-melezitose *O*-rhamnoside. However, there were other differences regarding the quantities of the detected metabolites; there were 53 metabolites that were differentially accumulated between both varieties (Supplementary Table S3). Thirty-nine and 14 metabolites had higher and lower content in QC as compared to JZ, respectively. Mainly, the compounds with higher content in QC were classified as amino acids and derivatives, organic acids, and phenolic acids. On the other hand, free fatty acids, nucleotides and derivatives, and saccharides and alcohols were present in higher quantities in JZ. This comparison indicates that 378 metabolites were not differentially accumulated between both varieties indicating that the secondary metabolite profile of both varieties is largely similar. This is in accordance with the observations made in PCA. Notably, the RC yam (belonging to Jiangxi) was also very closely grouped with Hubei varieties. From this observation, we expected a similar or nearly similar secondary metabolite profile. It can be seen from Figure 1D that there were 91 and 62 DAMs between RC vs. JZ and RC vs. QC, respectively. Thus, the metabolite profile of RC is more similar to QC based on these statistics.

### 3.4. Accumulation of Metabolites Related to Amino Acid Biosynthesis Pathway

Based on the observation that the number of metabolites related to amino acid and derivatives was high, we specifically checked the content of these metabolites among the yam samples. We detected a maximum content of L-glutamic acid and minimum content of L-alanine in HS. All the varieties had high content of L-glutamic acid, while the amino acids pathway related metabolites with lowest content varied in other genotypes (Table 1). We observed that 2-hydroxy-2-methyl-3-oxobutanoic acid content was high in HS, MS, and WS. Two varieties, i.e., QC and RC, were devoid of shikimic acid, while WS showed the highest content among all the varieties. Sum of the contents of all the metabolites related to amino acid biosynthesis pathway was lowest in MS (68597970.3) and highest in RC (171932218). Considering this, RC can be regarded as an amino acid rich variety. Interestingly, these two varieties, i.e., QC and RC, also showed higher contents of most of the polysaccharides and alcohols. In addition, HS was also rich in polysaccharides and alcohols. Thus, these three varieties must be considered for their utility in food, functional food, and medicines.

### 3.5. Key Medicinal Metabolites

**Figure 5:**
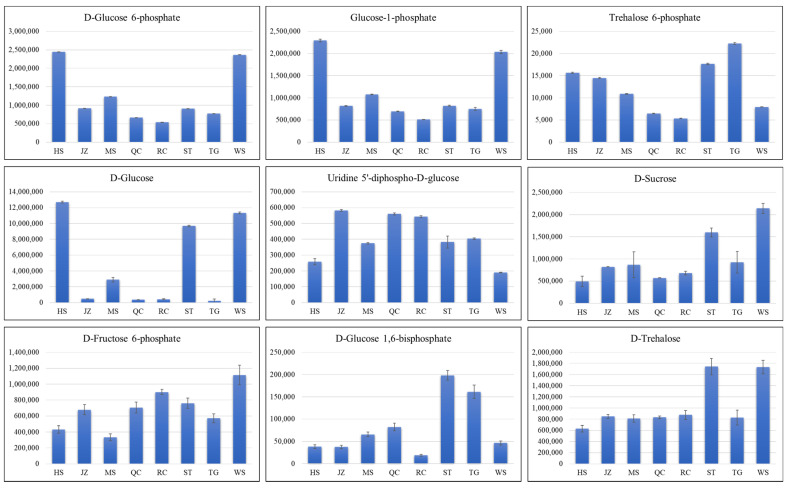
Contents of metabolites that were enriched in starch and sucrose biosynthesis pathway. *Y*-axis shows the amount of each metabolite, and the *x*-axis represents the eight yam varieties i.e., Hebei Ma Yam (MS), Henan Huai Yam (HS), Henan Tiegun Yam (TG), Henan Wild Yam (WS), Hubei Qichun Jiaozhang Yam (JZ), Hubei Qichun Yam (QC), Jiangxi Ruichang Yam (RC), and Shandong Tiegun Yam (ST). The error bars represent standard deviation.

**Figure 6:**
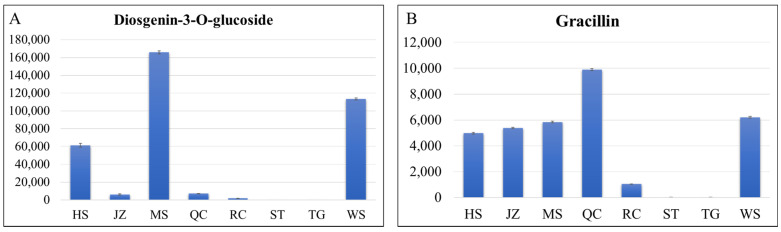
Contents of (**A**) diosgenin-3-*O*-glucoside and (**B**) gracillin in the tubers of eight yam varieties. *Y*-axis shows the amount of each metabolite, and the *x*-axis represents the eight yam varieties i.e., Hebei Ma Yam (MS), Henan Huai Yam (HS), Henan Tiegun Yam (TG), Henan Wild Yam (WS), Hubei Qichun Jiaozhang Yam (JZ), Hubei Qichun Yam (QC), Jiangxi Ruichang Yam (RC), and Shandong Tiegun Yam (ST). The error bars represent standard deviation.

Chinese yams are known for their utility in traditional medicine due to the presence of specific metabolites, e.g., starches, vitamins, diosgenin, and other saponins [7]. Our metabolome analysis revealed that the metabolites that were enriched in starch and sucrose biosynthesis pathway had variable content in the tubers of the eight yam varieties (Figure 5). It was observed that in most cases, the contents of the metabolites in this pathway were high in HS and WS. Specifically, the contents of D-glucose-6P, glucose-1P, D-glucose, D-sucrose, D-fructose-6P, and D-trehalose were highest in the WS. One major use of yams is for the extraction of diosgenin. Three varieties i.e., MS, HS, and WS had highest trillin (diosgenin-3-*O*-glucoside) content (Figure 6A). Another steroidal saponin, i.e., gracillin had highest content in QC (Figure 6B). Among vitamins, we detected fifteen vitamins in all varieties. Notably, we found that all varieties had riboflavin, with HS having the highest concentration. TG and ST had highest contents of vitamin C (Table 2). These results imply that when considering medicinal uses of *D. polystachya,* respective bioactive concentrations of each variety should be considered.

## 4. Discussion

### 4.1. D. polystachya Is Rich in Amino Acids, Polysaccharides, and Phenolic Acids

Chinese yam tubers are consumed directly as vegetables and its flour is used for baking and making noodles [41]. Our results on the number of detected metabolites and their specific classes indicated high amino acids, polysaccharides, and phenolic acid contents (Figure 2). The number of detected metabolites in our study is higher than the previous reports in *Dioscorea* spp. [18]. For example, in 49 accessions belonging to four *Dioscorea* spp. only 200 compounds were measured [16,18]. The differences could be because of different species under investigation since our study only included *D. polystachya* varieties or extraction and analytic methodology. On the other hand, the higher contents of phenolic acid and polysaccharide metabolites were expected since it is known that yam tubers are rich in starch, polysaccharides, and proteins [42,43]. These observations provide detailed insight into each detected metabolite and its contents. Such details offer an increased resolution within one tissue of one yam species and are greatly helpful for future studies to focus on specific type of metabolites and their respective pathways. A very high number of detected metabolites that were classified as amino acids and derivatives is indicative of higher protein contents. The amino acid profiles suggest *D. polystachya* varieties as a good source of amino acids which is consistent with the results of an earlier study which also revealed that wild yam has significant content of essential and non-essential amino acids [44]. Taken together, it could be stated that based on the metabolomic profiles, the Chinese yam tubers of different varieties offer calories, nutrients, and bioactive compounds when consumed freshly harvested.

### 4.2. D. polystachya Varieties Differ in Metabolome Profiles

Different *Dioscorea* spp. have been previously explored for their tuber and/or leaf metabolome and it was reported that yams differ in their metabolome profiles [5,16,18]. Our study is a pioneering attempt to explore and understand the diversity and abundance of metabolites within *D. polystachya*. Wild yams have long been used for the medicinal and food purposes. This could be possibly due to its readily availability in respective natural habitats as well as due to its unique bioactive compound profiles [45]. We found that WS was rich in polysaccharides e.g., D-fructose, D-galactose, and D-mannose, and the other varieties have lower contents (Supplementary Table S1). This could be due to the different soil profiles and or cultivation practices being followed for growing the varieties. Such differences have been previously explored in other tuber crops and it was suggested that sucrose metabolism might be differently regulated [46]. A higher number of metabolites detected in HS, MS, and WS suggests that these tubers could have increased metabolic activity at the time of harvesting. For example, lower number of metabolites classified as phenolic acids in RC could be due to different natural and growing conditions as observed previously in different potato varieties [47]. Changes in phenolic compound contents have also been linked to prevalent abiotic stress conditions such as drought [48,49,50] and salinity [51,52]. A detailed investigation on prevalence and effect of such conditions within *D. polystachya* spp. is a promising avenue to be explored. The diversity in terms of contents of specific metabolites, e.g., amino acids biosynthesis pathway-related metabolites is also an interesting observation in our study. We say this because eight of the nine essential amino acids (except L-lysine) were in high quantities in all tested varieties. Yams are a major share in the diet of Chinese (17% of yam exports) and, therefore, yams provide essential (as well as non-essential) amino acids to these populations and contribute to their health. A balanced portion of yams could possibly constitute an optimal nutrition for such populations [53]. From the agronomic perspective, the amino acid profile of the studied varieties is indicative of relatively different agronomic advantage in different growing areas of China as reported in most of the root and tuber crops [6]. Wild yams have been proven to be a good source of amino acids [44]; however, overall, relatively abundant metabolites related to amino acid biosynthesis pathway in all tested varieties indicates that regardless of the variety, *D. polystachya* spp. is a rich source of amino acids and when consumed raw or processed should be considered a nutritionally rich diet. Overall, the three varieties with rich metabolomic profile, i.e., HS, MS, and WS could be regarded as nutritionally valuable as compared to the others. When, the varieties are considered from a geographical point of view, it is proposed that QC and JZ are nutritionally similar, while RC presents a more similar profile to QC. The higher contents of amino acid and derivatives, organic acids, and phenolic acids in QC make it nutritionally superior to JZ. On the other hand, the higher polysaccharide and derivatives, and free fatty acid profile of JZ is also interesting. Hence, both Hubei varieties should be preferably used from their secondary metabolite profile presented in this study.

### 4.3. Different D. polystachya Varieties Offer Different Utility Based on Metabolome Profiles and Contents

Yam is used in traditional Chinese medicine for centuries and China has been regarded as an isolated domestication center of yam. Despite such a large-scale production and economical importance in food and traditional medicine, the biochemical characterization of yam varieties being produced in China is scarce. The knowledge on the presence of specific metabolites in these varieties is a fruitful addition and will strengthen the choice of varieties for particular purposes. The metabolome profiles of eight varieties revealed in this study has put forward interesting and useful information for the utility of these genotypes. The most important bioactive compound that is extracted from yam is diosgenin, which has a broad medicinal value, e.g., it enhances cognitive function and has hyperlipidemic, hyperglycemic, antioxidant, anti-inflammatory, and antiproliferative properties [54,55]. Previously, it has been widely extracted from wild yams and converted into steroids, estrogen, and dehydroepiandrosterone [55]. From this point of view, the high content of diosgenin-3-*O*-glucoside in MS and WS makes them highly useful varieties for large-scale diosgenin extraction. Furthermore, a comparative genomic study could reveal the genetic mechanism of higher diosgenin production in both MS and WS and no production (as per our metabolome results) in ST and TG. A high proportion of yams is used for nutritional purposes in China [45] and, therefore, both ST and TG are rich in amino acids and derivatives as well as organic acids and phenolic acids. Thus, the metabolite profiles of each of the tested varieties offer unique uses. On the other hand, the high contents of riboflavin in JZ, HS, and WS make these varieties useful for the large-scale production of riboflavin or as a preferred variety to feed patients with vitamin B deficiency. A previous study has suggested that yam species may differ in riboflavin content and the method to prepare yam flour may affect its content [56]. Our results suggest that some varieties within a species may have relatively higher riboflavin content. This could be due to the different growing or environmental conditions of the studied yam varieties [56]. One of the main reasons for high yam utility as food is high polysaccharide contents. The high contents of polysaccharides in all yam varieties are indicative of their nutritive value. However, the richest was WS. This report is consistent with previous studies supporting large-scale wild yam usage in food and medicine industry [7].

### 4.4. Future Research on D. polystachya

Exploration of metabolite profile of each of these eight varieties has given a detailed biochemical picture of the yam tubers. We have discussed variety specific uses. The future studies on *D. polystachya* varieties should focus on the account of environmental, geographical, and climatic effects on the concentration of the specific metabolites. This in turn will enable researchers to produce yams with higher/lower contents of specific metabolites. Another interesting question that future studies should address is the genetics of high/low or no presence of specific metabolites in the varieties. For example, the lack of detection of diosgenin and gracillin in some varieties and relatively high amounts detected in WS should be explored from transcriptome and genetics point of view. Moreover, the relatively similar profiles of QC, JZ, and RC also invite studies on regional climate effects on varieties and their nutritional contents. Such studies would enable breeding of yams with specific nutritional profiles.

## 5. Conclusions

This is a first large-scale metabolome profiling study of eight *D. polystachya* varieties. All the studied yam varieties/genotypes are rich in amino acids and derivatives, phenolic acids, organic acids, saccharides, and alcohols. Palmitaldehyde and L-glutamic acid were among the top-10 most accumulated metabolites in all varieties suggesting that all yam genotypes can be a rich source of amino acids and derivatives. Highest saccharide contents, diosgenin, vitamins, and other polysaccharides in WS make it a nutritionally favorable genotype and it should be included in yam breeding and improvement programs. The comparative metabolome profiles indicated that within varieties, the number of detected metabolites and their respective concentrations differed. Three varieties, i.e., HS, MS, and WS, had the highest number of detected metabolites, suggesting that these should be preferred for nutritional and medicinal purposes.

## Figures and Tables

**Table 1:** Quantities (relative content) of the amino acid biosynthesis pathway related metabolites in eight yam varieties.

Compound Name	HS	JZ	MS	QC	RC	ST	TG	WS
2-Hydroxy-2-methyl-3-oxobutanoic acid	20,189	2774	20,750	13,369	9100	8501	9	29,304
L-Asparagine	512,580	246,787	438,293	212,913	903,170	162,117	314,913	991,700
Shikimic acid	280,557	10,818	325,290	9	9	108,597	166,289	1,528,013
L-Glycine	54,524	31,847	99,641	46,566	166,227	31,485	61,560	91,819
L-Aspartic Acid	179,627	383,653	173,230	292,570	454,543	276,557	400,117	298,600
L-Leucine	1,023,030	217,523	504,200	1,086,607	883,730	264,000	655,027	1,113,757
L-Threonine	8,872,567	4,996,267	6,290,300	7,236,100	12,276,333	7,484,467	5,347,000	9,397,000
L-Tyrosine	7,563,567	4,342,400	3,939,633	23,188,433	30,096,333	2,100,900	1,447,500	6,041,733
L-Histidine	3,163,367	5,547,900	2,477,800	10,705,967	12,294,433	4,492,767	2,030,200	5,124,167
L-Valine	4,869,233	936,337	2,946,067	3,401,400	3,674,300	917,830	2,068,267	3,873,667
L-Isoleucine	1,031,653	216,743	489,500	1,065,467	876,860	287,447	669,460	1,132,440
L-Arginine	1,092,357	1,852,067	1,098,087	1,773,967	1,945,767	684,817	860,083	413,363
L-Tryptophan	3,141,013	820,333	308,770	15,000,333	2,424,500	13,194,333	3,090,397	9,606,433
L-Homoserine	9,104,733	5,182,267	6,424,467	7,368,867	1,295,6333	7,814,533	5,731,633	9,846,433
O-Acetylserine	56,170	56,702	76,012	42,476	51,966	23,477	35,622	66,658
Phosphoenolpyruvate	68,188	66,762	47,585	42,489	77,714	89,989	73,858	64,938
Argininosuccinic acid	95,243	75,388	93,438	59,969	60,440	30,598	46,636	165,560
2-Isopropylmalic Acid	1,018,967	75,689	507,123	160,350	133,218	992,057	677,057	296,380
L-Proline	798,060	868,233	527,353	1,769,780	1,407,867	199,073	249,720	730,737
L-Citrulline	4,798,100	7,407,300	4,684,833	2,993,233	11,067,267	8,424,600	5,464,200	1,092,600
L-Serine	8,318,867	4,711,400	7,853,300	5,979,333	9,210,933	4,236,267	4,128,400	9,583,200
L-Glutamic acid	17,475,000	38,746,667	18,312,667	32,689,667	40,976,000	38,307,667	28,774,000	31,668,333
L-Phenylalanine	3,713,100	1,881,600	2,317,600	13,634,900	5,681,867	667,383	768,980	5,905,200
L-Lysine	197,727	297,223	146,947	395,507	468,727	181,067	109,435	161,207
N-Acetyl-L-glutamic acid	113,515	377,400	361,547	226,463	466,277	272,503	443,220	159,013
L-Glutamine	188,893	267,073	148,850	399,223	439,100	166,707	107,900	150,788
L-Cysteine	36,773	81,414	39,362	50,923	49,264	34,895	95,566	35,369
2,6-Diaminooimelic acid	546,180	464,830	250,790	645,430	1,786,833	1,150,607	656,837	850,843
L-Methionine	1,532,673	248,787	925,987	1,233,383	1,294,523	257,847	884,340	1,868,263
L-Saccharopine	17,265	18,701	18,410	23,905	26,602	46,919	33,352	25,745
L-Alanine	7673	2188	17,661	6898	10,939	2125	3706	4032
α-Ketoglutaric acid	178,203	136,004	109,423	90,059	117,576	107,107	30,908	129,640
L-Ornithine	135,533	293,903	97,413	249,387	418,970	105,368	64,881	31,494
S-Adenosyl-L-methionine	42,219	19,932	26,620	34,835	30,260	19,435	14,517	16,205
L-Cystathionine	283,277	607,923	328,547	1,127,953	1,119,487	1,298,530	641,040	558,327
N-α-Acetyl-L-ornithine	6,323,067	17,055,667	6,126,967	13,141,000	18,058,667	4,866,900	5,826,533	1,909,933
Dihydroxyacetone phosphate	90,476	14,331	29,318	14,343	11,172	38,319	21,761	37,559
D-Erythrose-4-phosphate	31,412	6884	14,192	6210	4911	13,696	6532	21,288

**Table 2:** Quantities (relative content) of the vitamins in eight yam varieties.

Compounds	HS	JZ	MS	QC	RC	ST	TG	WS
Riboflavin (Vitamin B2)	3124	1860	2374	2300	2087	2722	1953	2406
N-(beta-D-Glucosyl)nicotinate	9360	8849	4451	7140	4050	3886	6298	4020
L-Ascorbic acid (Vitamin C)	52,139	3397	60,939	1751	1590	192,033	290,330	6761
Biotin	7817	12,808	12,286	11,324	14,041	10,141	8462	8709
Delta-Tocopherol	10,716	11,074	11,677	11,140	10,617	11,789	11,365	11,465
Pyridoxal	9895	15,313	8158	7995	7228	6595	6298	12,730
Nicotinic acid (Vitamin B3)	13,554	13,904	24,652	20,598	23,574	29,894	26,770	18,077
Orotic acid	496,593	37,893	43,963	23,132	16,767	213,033	46,264	58,923
Pyridoxine-5′-*O*-glucoside	244,953	446,547	131,430	367,150	129,638	193,710	187,913	94,560
Menatetrenone (Vitamin K2)	107,573	115,347	113,775	91,789	90,104	89,383	93,762	99,026
Dehydroascorbic acid	156,740	371,043	210,967	422,020	208,047	212,207	267,823	194,550
Nicotinamide	263,703	355,080	573,840	340,820	275,997	288,603	1,520,000	295,040
Pyridoxine	17,721	68,595	1,215,400	54,216	35,292	64,701	461,267	1,143,010
D-Pantothenic acid	1,330,137	1,817,033	2,018,067	2,388,933	1,475,200	2,063,467	2,351,233	1,976,033
Nicotinate D-ribonucleoside	1,136,437	2,357,433	2,083,867	2,711,567	1,604,133	3,770,933	3,500,067	2,302,200

## Data Availability

All data used in this study can be found in the text or its supplementary files. Additional data sets related to metabolites can be accessed by directly contacting the corresponding author.

## Supplementary Materials

The following are available online at https://www.mdpi.com/article/10.3390/life11070687/s1. Table S1: Detailed report on the detected metabolites, their classification, and content in the tubers of eight Chinese Yam varieties. Table S2: Lists of top-10 and least-10 accumulated metabolites in each of eight Chinese Yam tubers. Figure S1: List of differentially accumulated metabolites between the two varieties belonging to Hubei, China (QC and JZ). Figure S2: Total ion chromatograms (TIC) of mass spectrometry detection of randomly selected 48 metabolites as an example.
