# Supplementary material for: Metabolome Profiling of Eight Chinese Yam (Dioscorea polystachya Turcz.) Varieties Reveals Metabolite Diversity and Variety Specific Uses
Source: Life (Basel). 2021 Jul 14;11(7):687. doi: 10.3390/life11070687 (PMC8308037; doi:10.3390/life11070687)
Supplement: Supplementary file 1 [file life-11-00687-s001.zip › Supplementary Figure 1.pdf]

Supplementary Figure 1. Total ion chromatograms (TIC) of mass spectrometery detection of randomly selected 48 metabolites as an example.

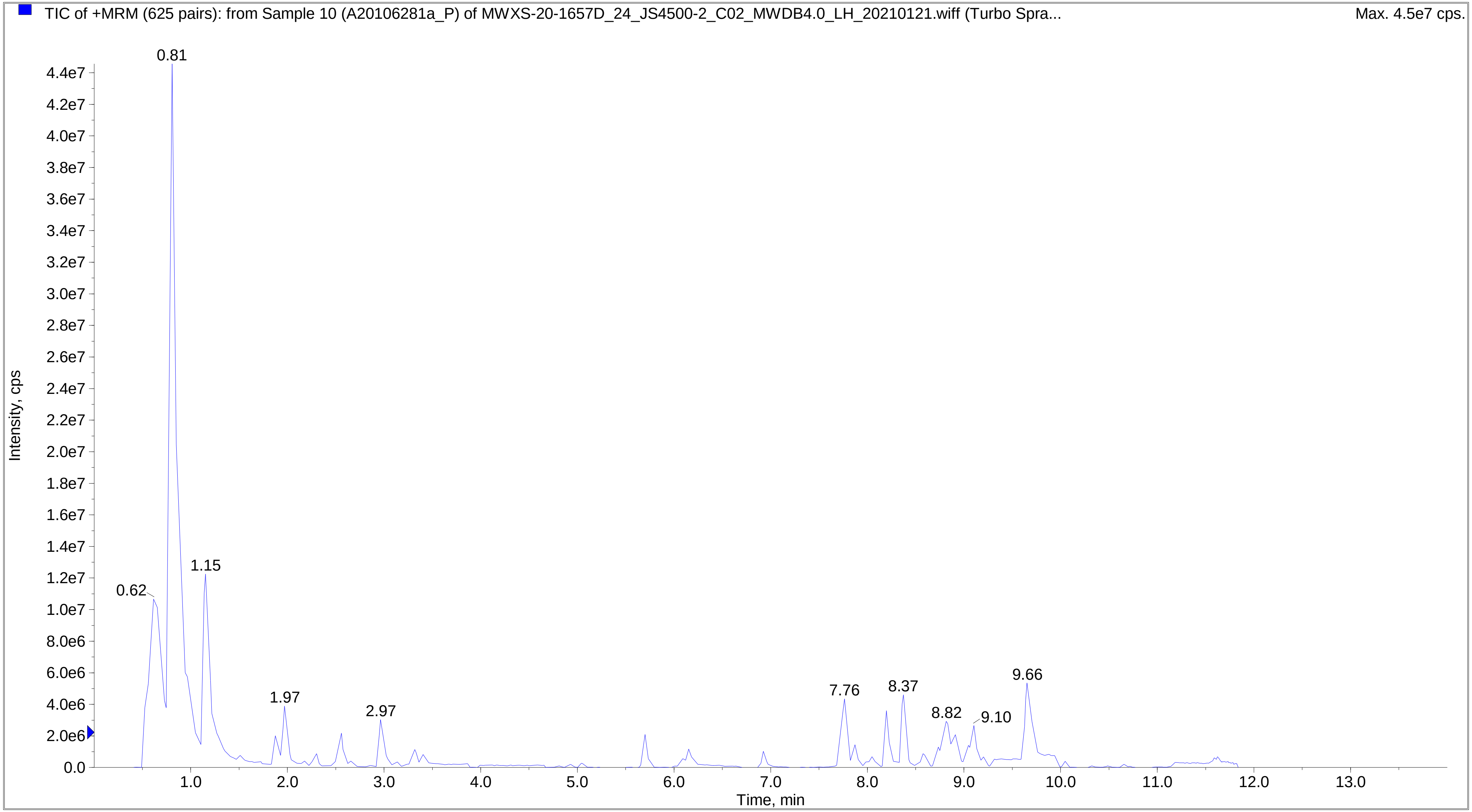
TIC of +MRM (625 pairs): from Sample 10 (A20106281a_P) of MWXS-20-1657D_24_JS4500-2_C02_MWDB4.0_LH_20210121.wiff (Turbo Spra...
Max. 4.5e7 cps.
Intensity, cps
Time, min
0.81
0.62
1.15
1.97
2.97
7.76
8.37
8.82
9.10
9.66
4.4e7
4.2e7
4.0e7
3.8e7
3.6e7
3.4e7
3.2e7
3.0e7
2.8e7
2.6e7
2.4e7
2.2e7
2.0e7
1.8e7
1.6e7
1.4e7
1.2e7
1.0e7
8.0e6
6.0e6
4.0e6
2.0e6
0.0
1.0
2.0
3.0
4.0
5.0
6.0
7.0
8.0
9.0
10.0
11.0
12.0
13.0

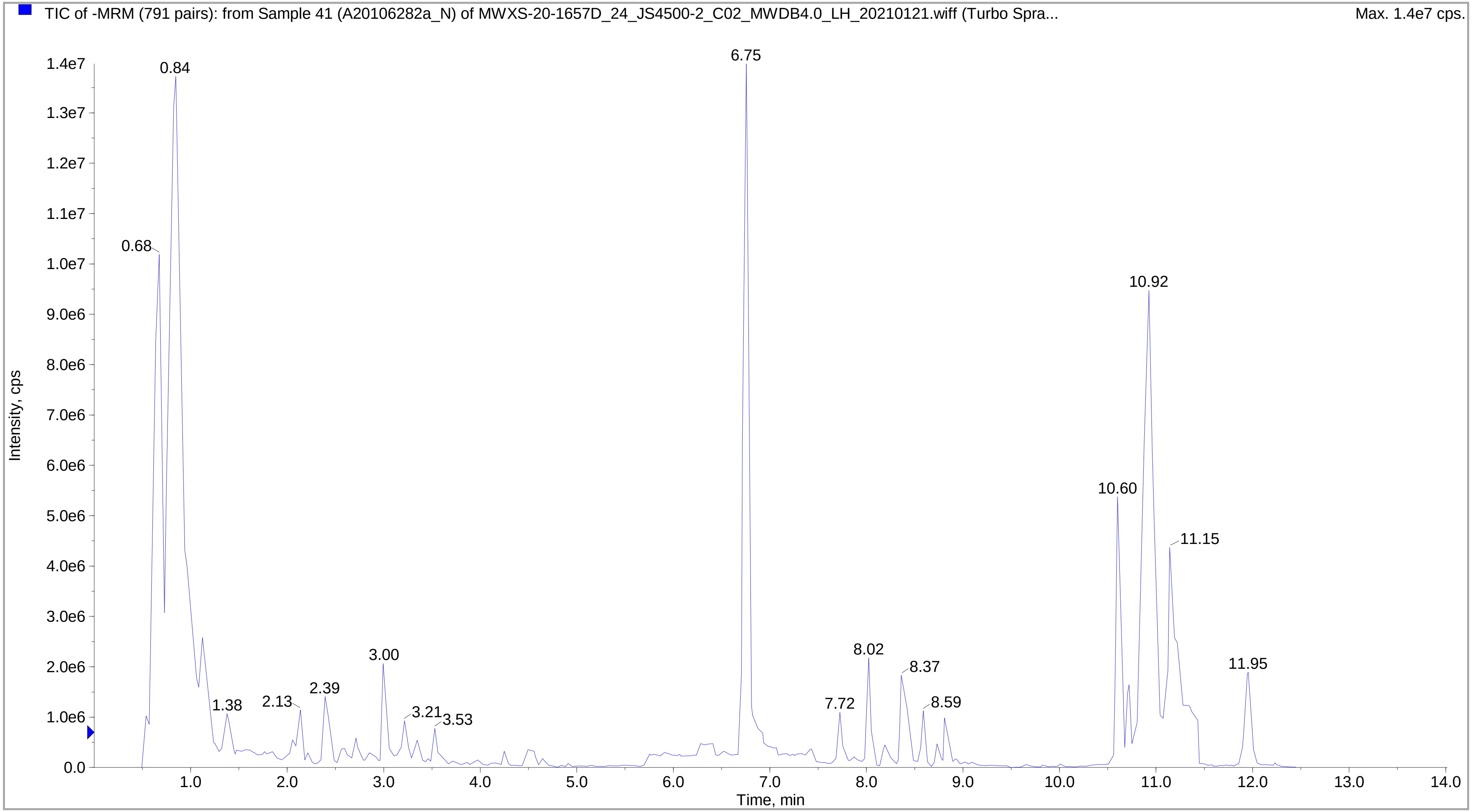

TIC of -MRM (791 pairs): from Sample 41 (A20106282a_N) of MWXS-20-1657D_24_JS4500-2_C02_MWDB4.0_LH_20210121.wiff (Turbo Spra...
Max. 1.4e7 cps.
Intensity, cps
Time, min
0.84
0.68
1.38
2.13
2.39
3.00
3.21
3.53
6.75
7.72
8.02
8.37
8.59
10.60
10.92
11.15
11.95

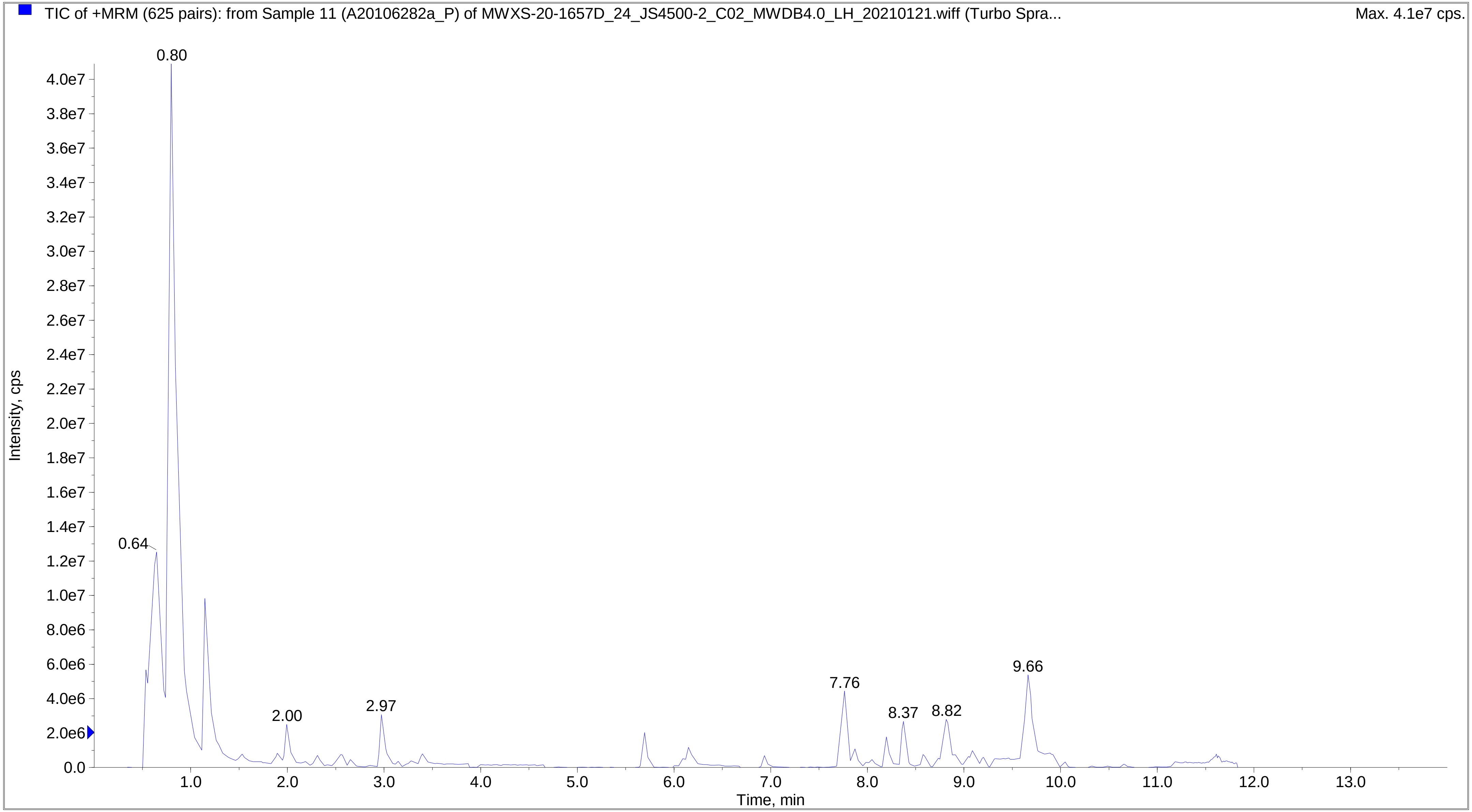

TIC of +MRM (625 pairs): from Sample 11 (A20106282a_P) of MWXS-20-1657D_24_JS4500-2_C02_MWDB4.0_LH_20210121.wiff (Turbo Spra...
Max. 4.1e7 cps.
Intensity, cps
Time, min
0.80
0.64
2.00
2.97
7.76
8.37
8.82
9.66
4.0e7
3.8e7
3.6e7
3.4e7
3.2e7
3.0e7
2.8e7
2.6e7
2.4e7
2.2e7
2.0e7
1.8e7
1.6e7
1.4e7
1.2e7
1.0e7
8.0e6
6.0e6
4.0e6
2.0e6
0.0
1.0
2.0
3.0
4.0
5.0
6.0
7.0
8.0
9.0
10.0
11.0
12.0
13.0

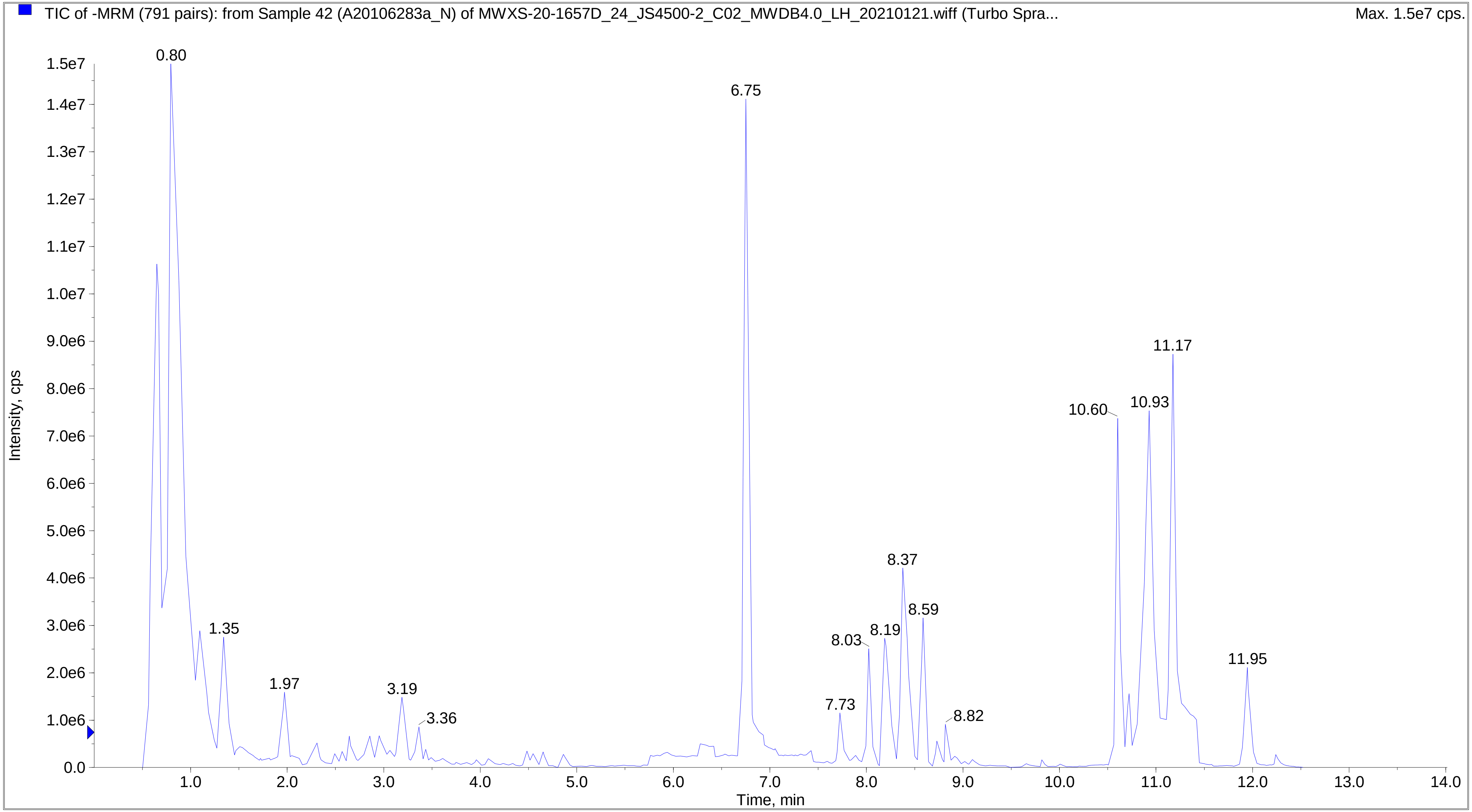

TIC of -MRM (791 pairs): from Sample 42 (A20106283a_N) of MWXS-20-1657D_24_JS4500-2_C02_MWDB4.0_LH_20210121.wiff (Turbo Spra...
Max. 1.5e7 cps.
Intensity, cps
Time, min
0.80
6.75
11.17
10.60
10.93
8.37
8.59
1.35
8.19
8.03
11.95
1.97
3.19
7.73
3.36
8.82
1.5e7
1.4e7
1.3e7
1.2e7
1.1e7
1.0e7
9.0e6
8.0e6
7.0e6
6.0e6
5.0e6
4.0e6
3.0e6
2.0e6
1.0e6
0.0
1.0
2.0
3.0
4.0
5.0
6.0
7.0
8.0
9.0
10.0
11.0
12.0
13.0
14.0

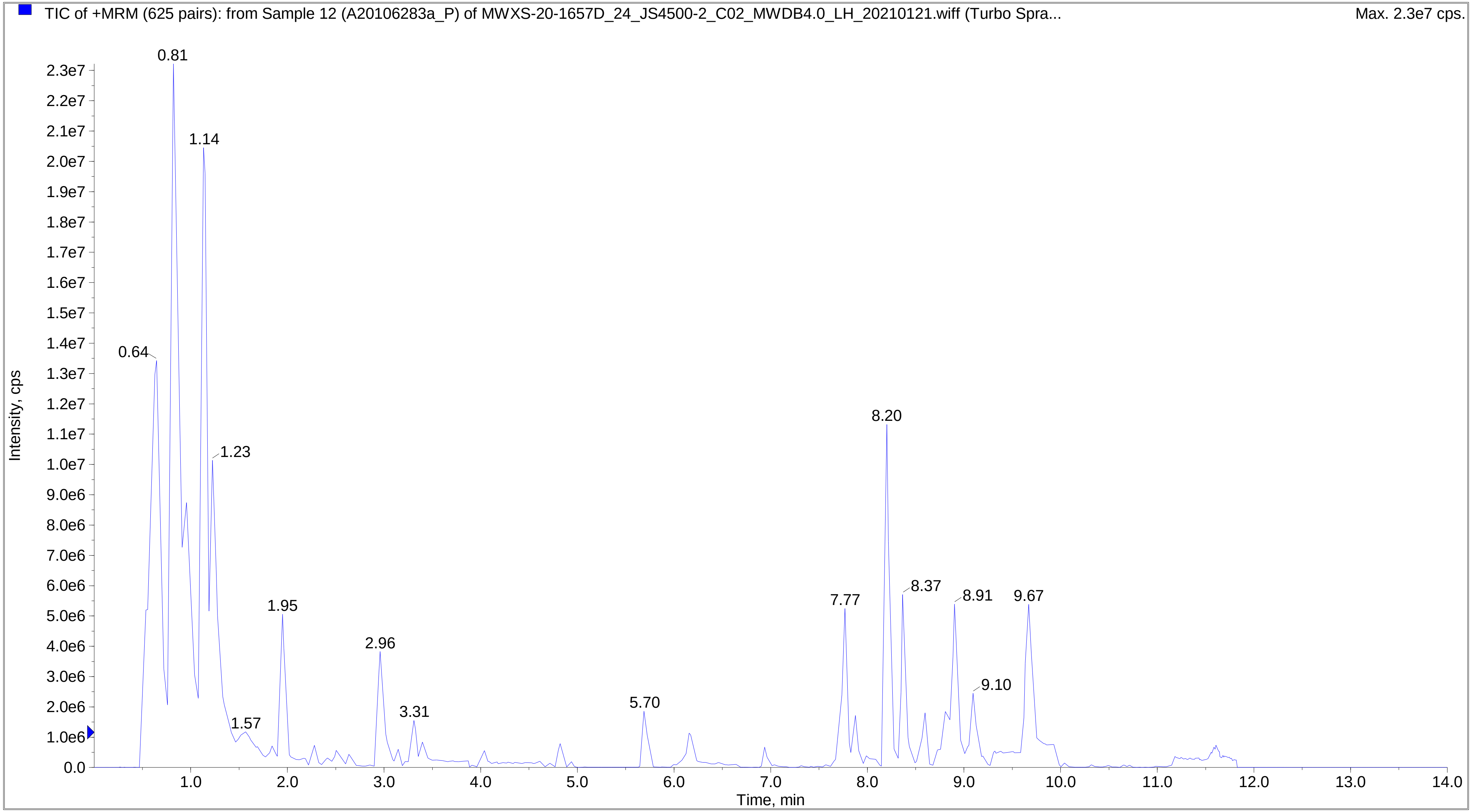

TIC of +MRM (625 pairs): from Sample 12 (A20106283a_P) of MWXS-20-1657D_24_JS4500-2_C02_MWDB4.0_LH_20210121.wiff (Turbo Spra...
Max. 2.3e7 cps.
Intensity, cps
Time, min
0.81
1.14
0.64
1.23
1.57
1.95
2.96
3.31
5.70
7.77
8.20
8.37
8.91
9.10
9.67

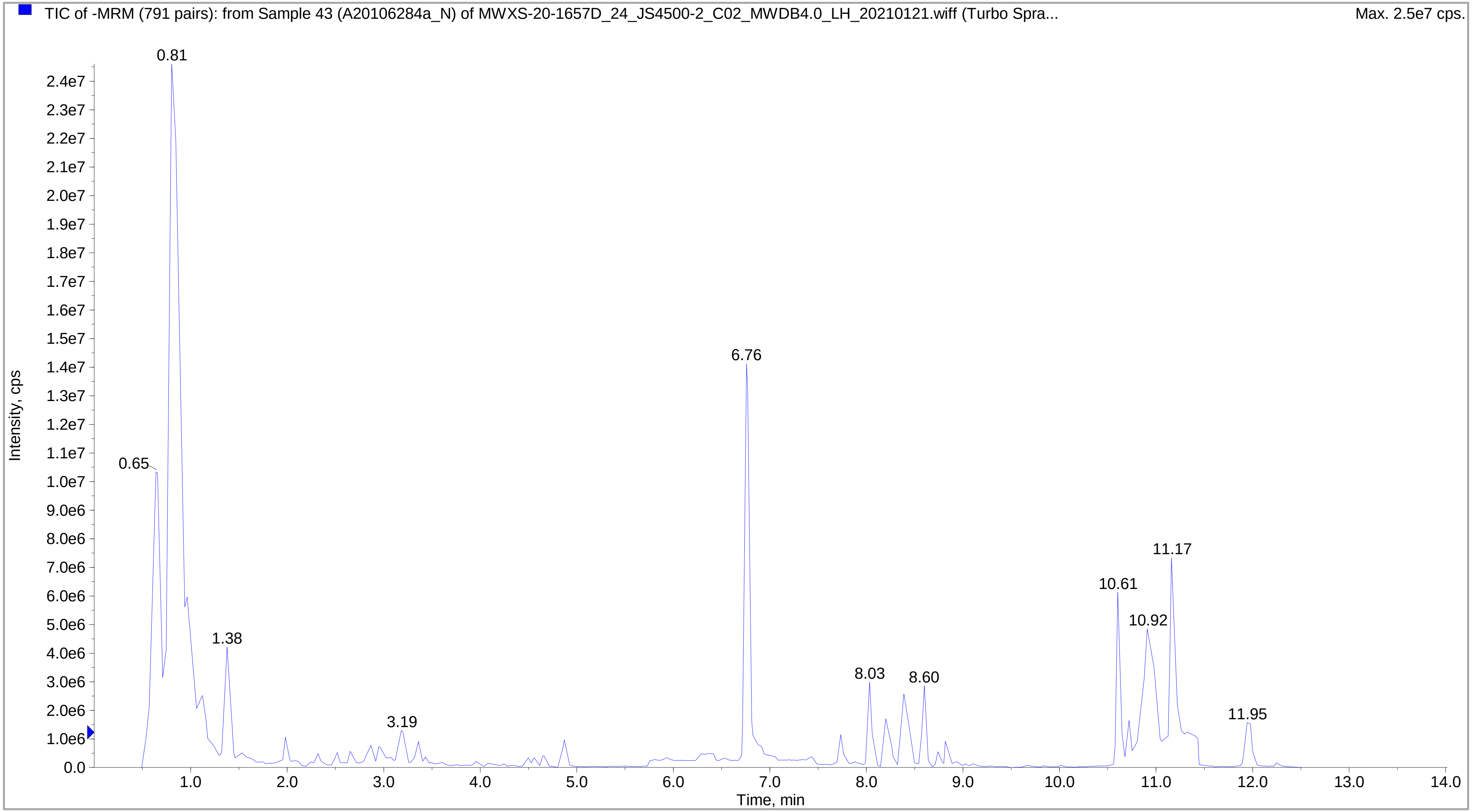

TIC of -MRM (791 pairs): from Sample 43 (A20106284a_N) of MWXS-20-1657D_24_JS4500-2_C02_MWDB4.0_LH_20210121.wiff (Turbo Spra...
Max. 2.5e7 cps.
Intensity, cps
Time, min
0.81
0.65
1.38
3.19
6.76
8.03
8.60
10.61
10.92
11.17
11.95

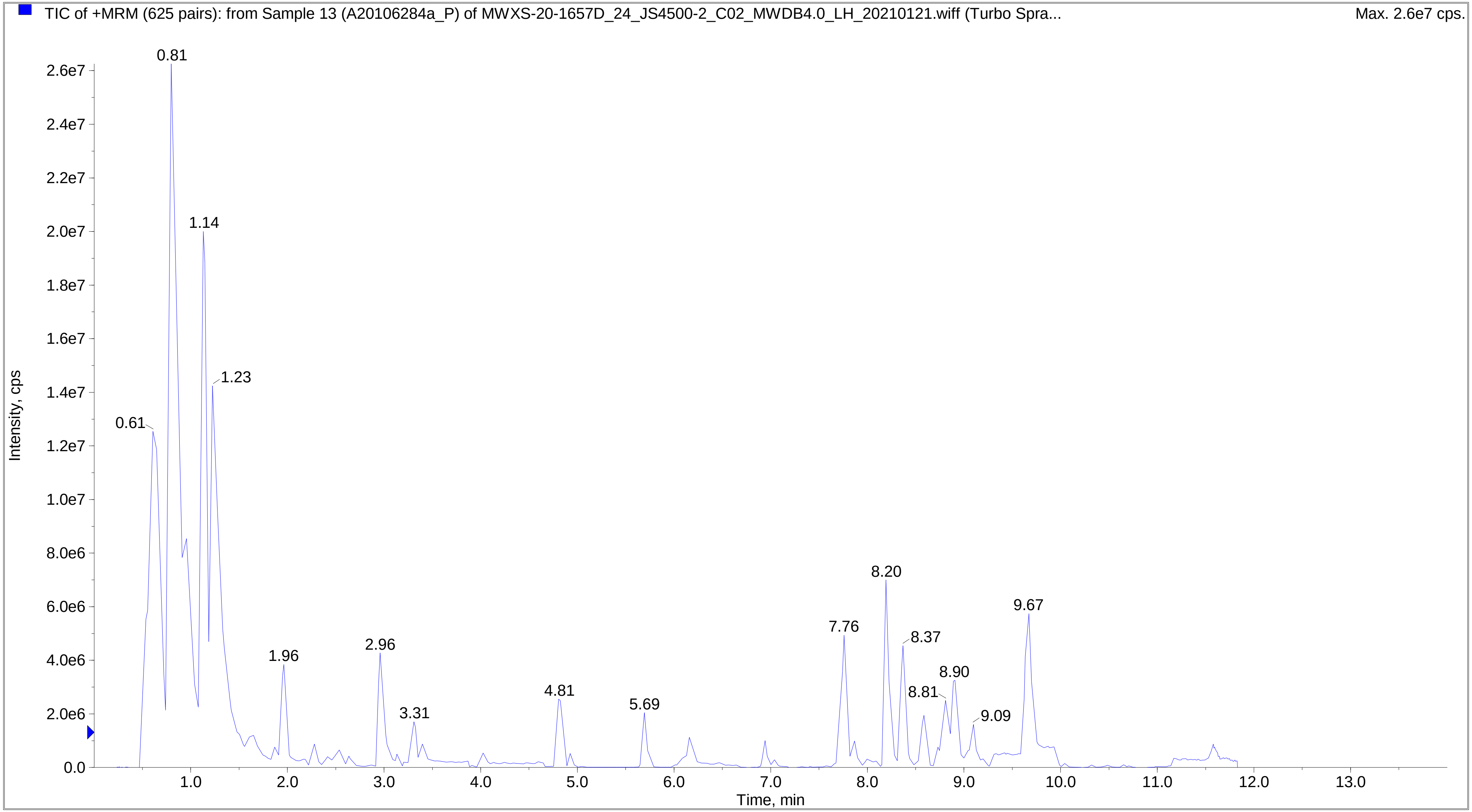

TIC of +MRM (625 pairs): from Sample 13 (A20106284a_P) of MWXS-20-1657D_24_JS4500-2_C02_MWDB4.0_LH_20210121.wiff (Turbo Spra...
Max. 2.6e7 cps.
Intensity, cps
Time, min
0.81
1.14
1.23
0.61
1.96
2.96
3.31
4.81
5.69
7.76
8.20
8.37
8.81
8.90
9.09
9.67

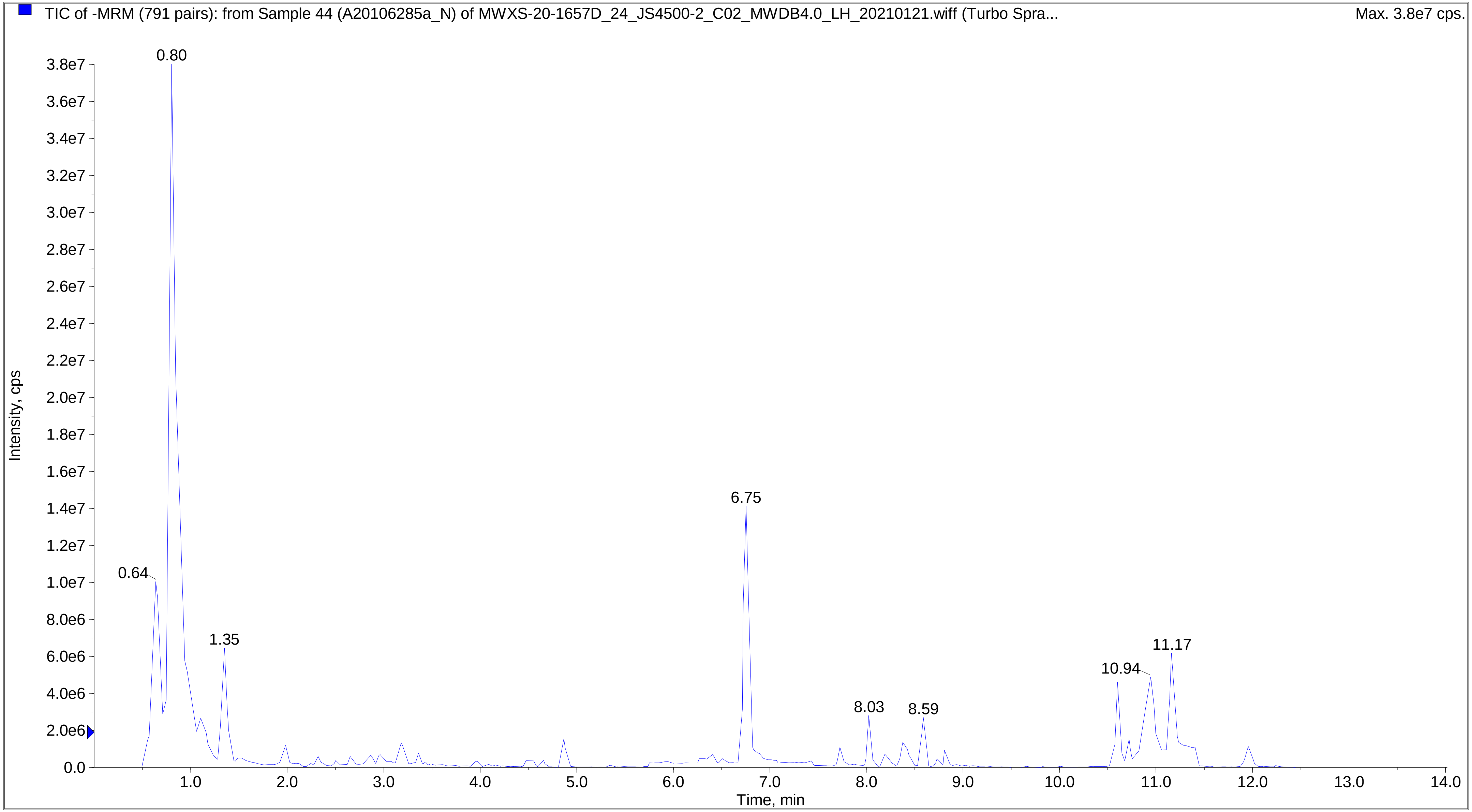

TIC of -MRM (791 pairs): from Sample 44 (A20106285a_N) of MWXS-20-1657D_24_JS4500-2_C02_MWDB4.0_LH_20210121.wiff (Turbo Spra...
Max. 3.8e7 cps.
Intensity, cps
Time, min
0.80
0.64
1.35
6.75
8.03
8.59
10.94
11.17

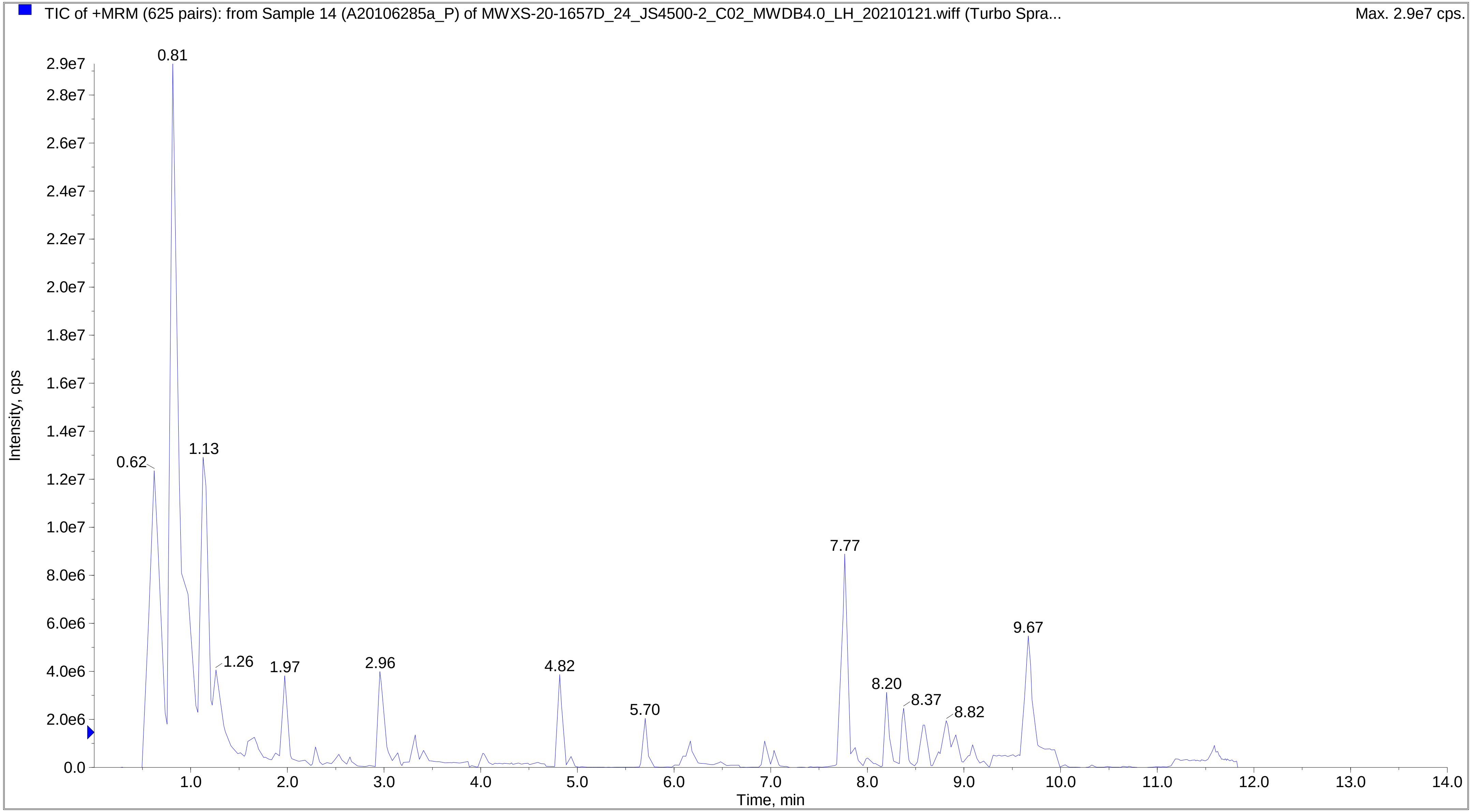
TIC of +MRM (625 pairs): from Sample 14 (A20106285a_P) of MWXS-20-1657D_24_JS4500-2_C02_MWDB4.0_LH_20210121.wiff (Turbo Spra...
Max. 2.9e7 cps.
Intensity, cps
Time, min
0.81
0.62
1.13
1.26
1.97
2.96
4.82
5.70
7.77
8.20
8.37
8.82
9.67

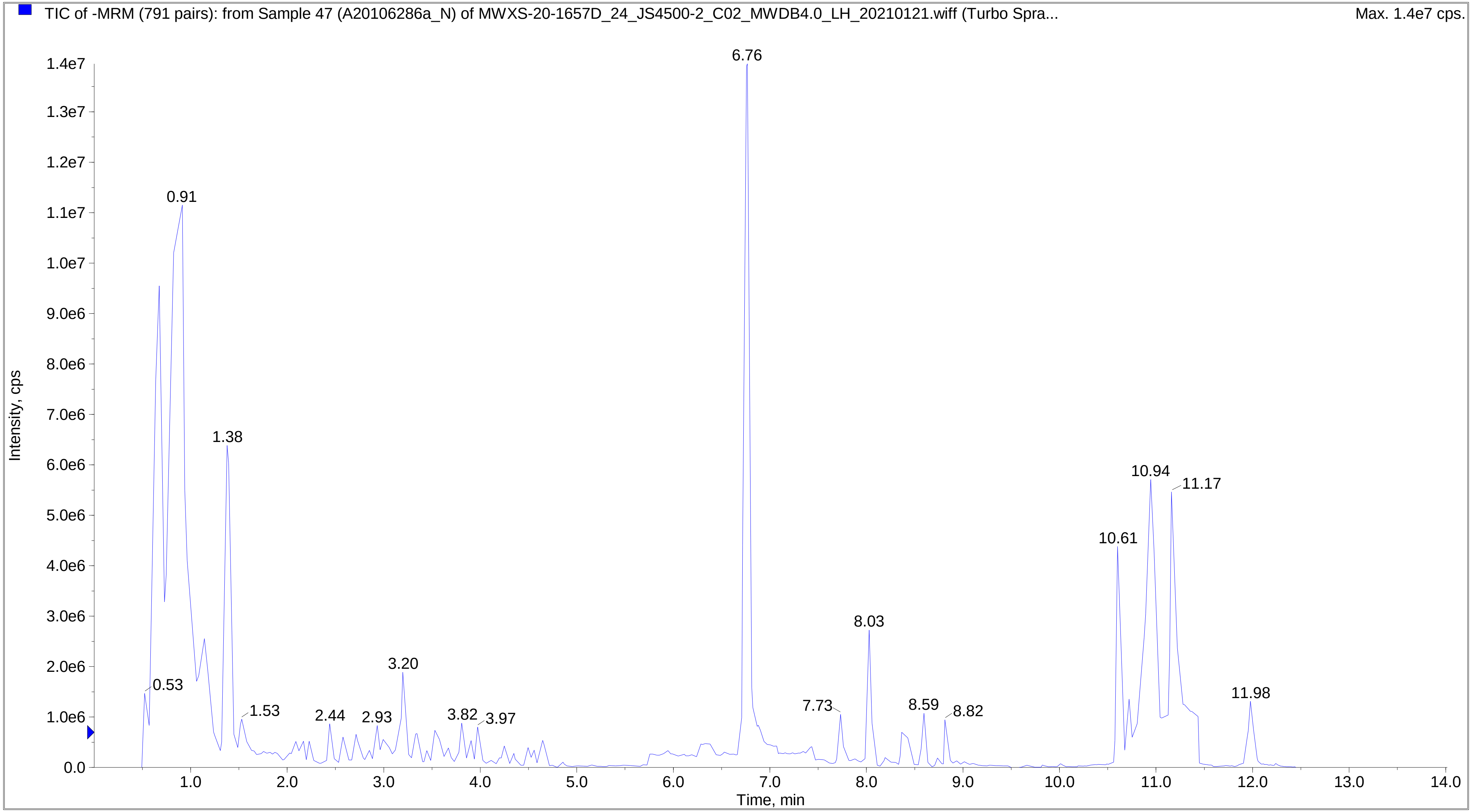

TIC of -MRM (791 pairs): from Sample 47 (A20106286a_N) of MWXS-20-1657D_24_JS4500-2_C02_MWDB4.0_LH_20210121.wiff (Turbo Spra...
Max. 1.4e7 cps.
Intensity, cps
Time, min
6.76
0.91
1.38
0.53
1.53
2.44
2.93
3.20
3.82
3.97
7.73
8.03
8.59
8.82
10.61
10.94
11.17
11.98

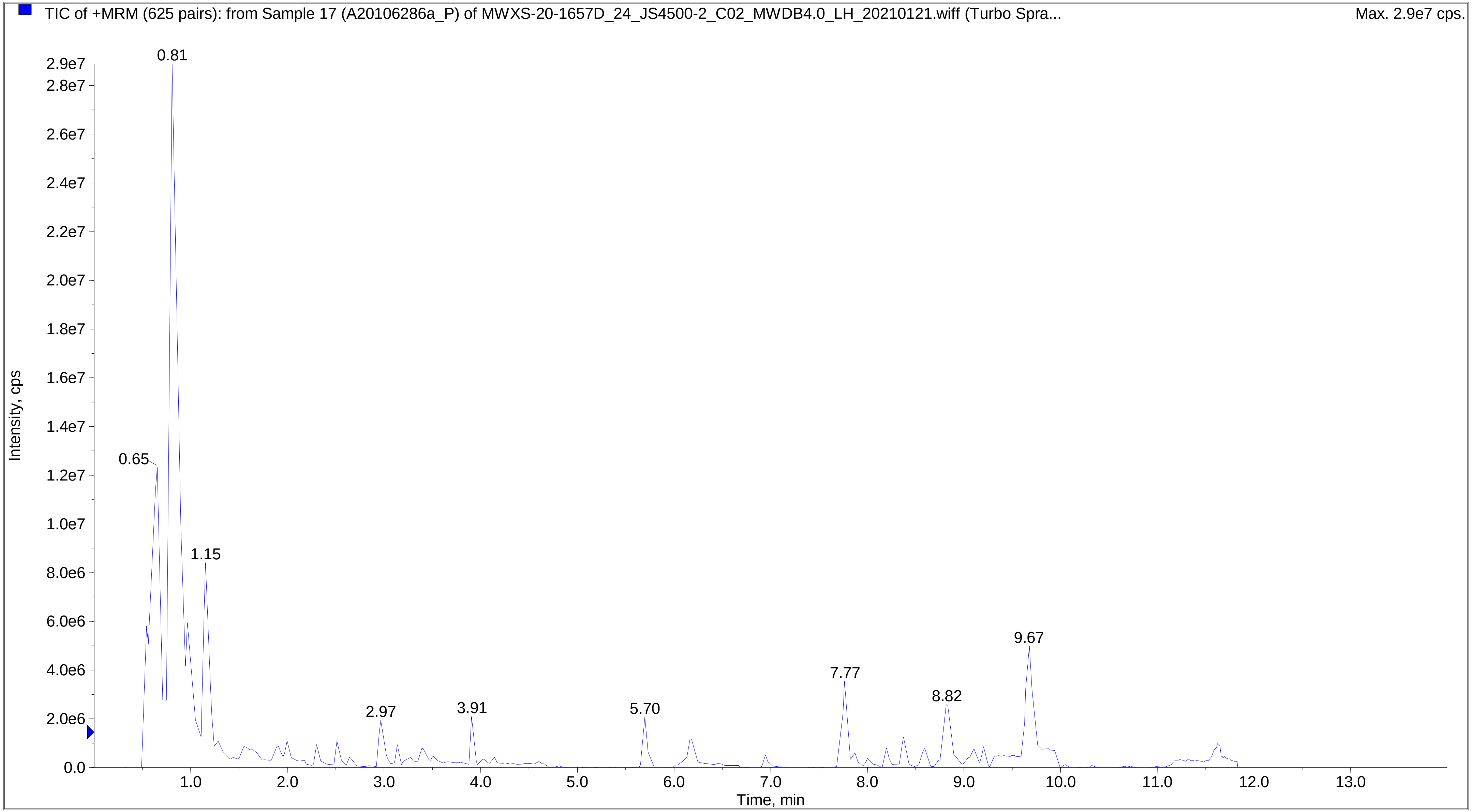

TIC of +MRM (625 pairs): from Sample 17 (A20106286a_P) of MWXS-20-1657D_24_JS4500-2_C02_MWDB4.0_LH_20210121.wiff (Turbo Spra...
Max. 2.9e7 cps.
Intensity, cps
Time, min
0.81
0.65
1.15
2.97
3.91
5.70
7.77
8.82
9.67

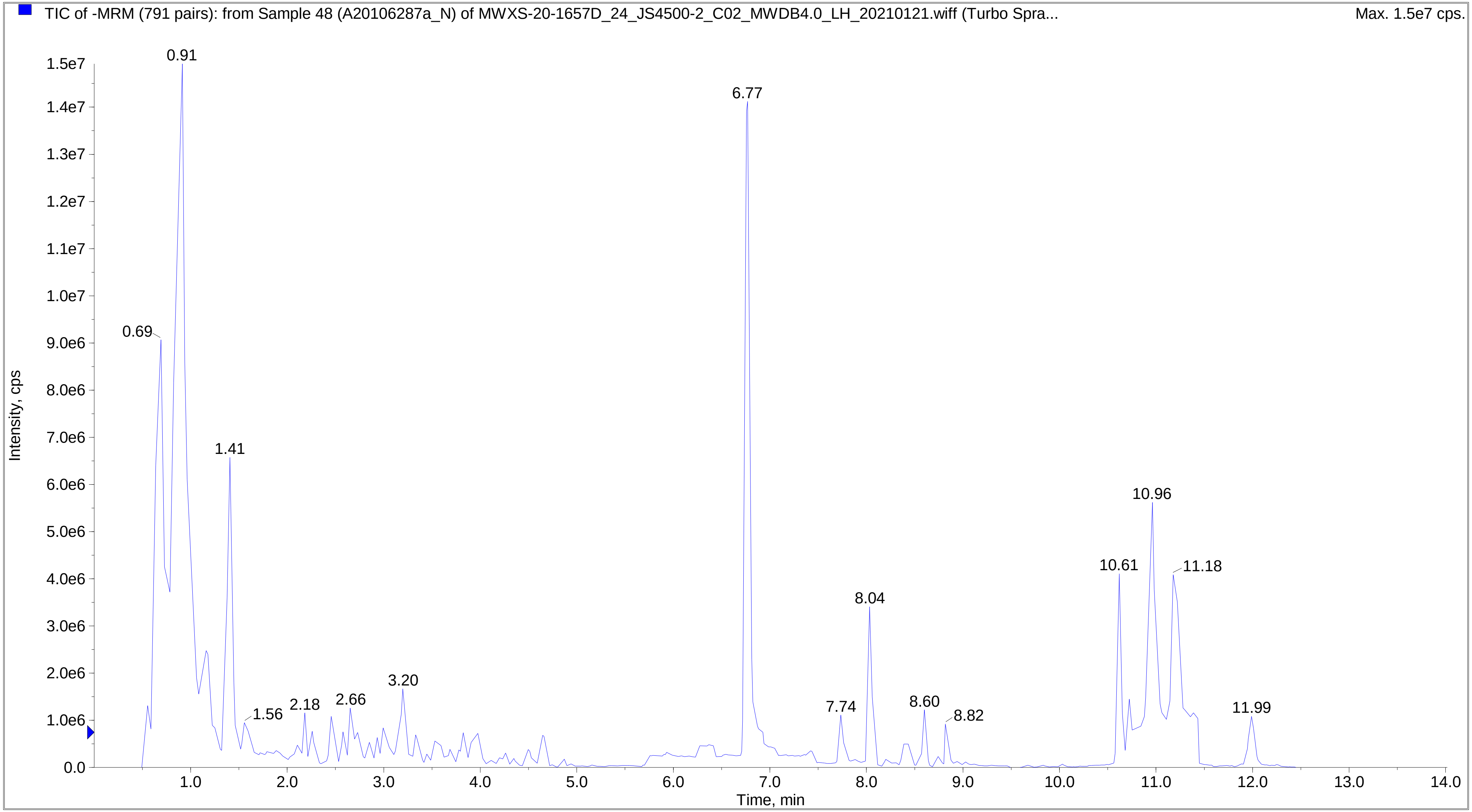

TIC of -MRM (791 pairs): from Sample 48 (A20106287a_N) of MWXS-20-1657D_24_JS4500-2_C02_MWDB4.0_LH_20210121.wiff (Turbo Spra...
Max. 1.5e7 cps.
Intensity, cps
Time, min
0.91
6.77
0.69
1.41
10.96
10.61
11.18
8.04
3.20
1.56
2.18
2.66
7.74
8.60
8.82
11.99

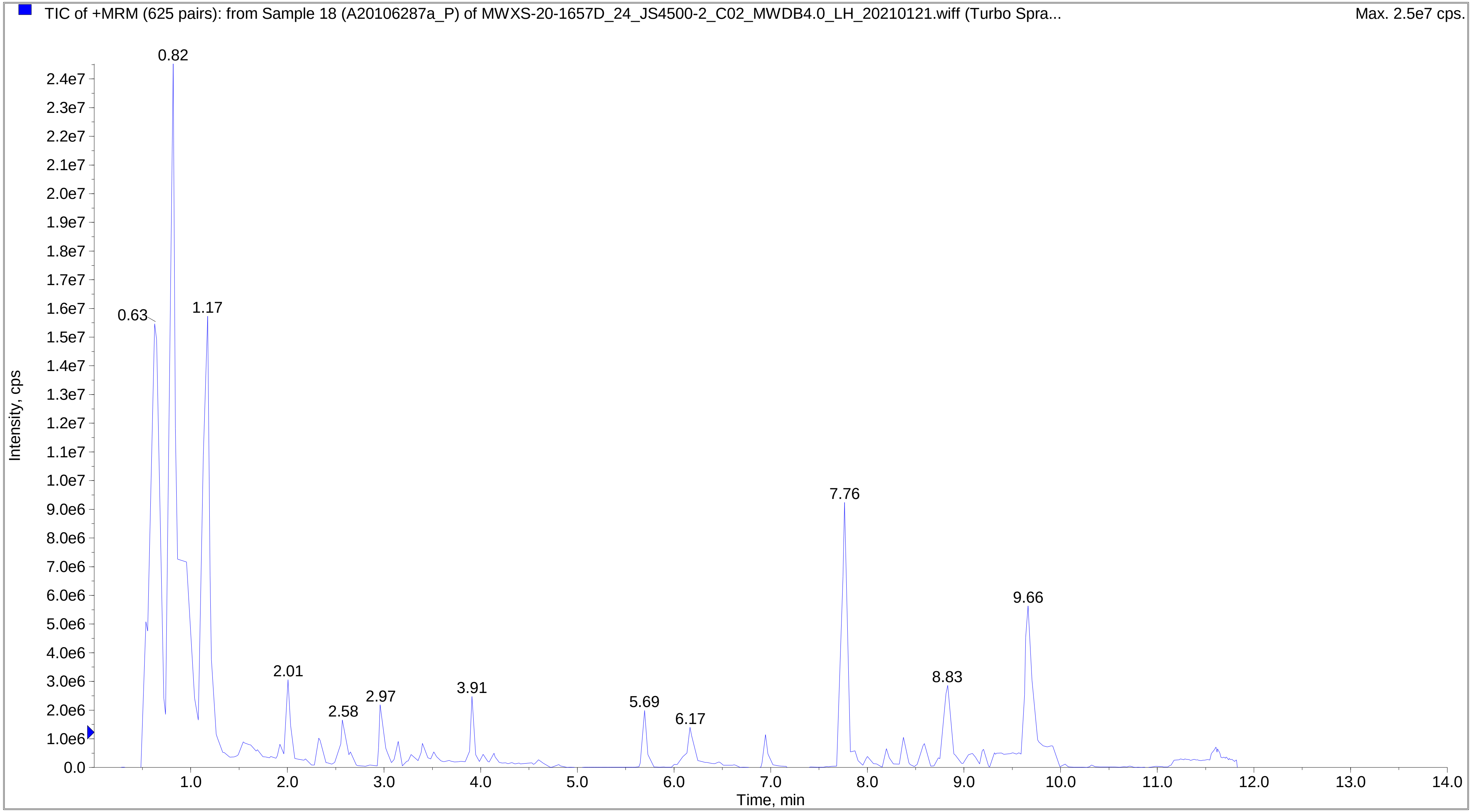

TIC of +MRM (625 pairs): from Sample 18 (A20106287a_P) of MWXS-20-1657D_24_JS4500-2_C02_MWDB4.0_LH_20210121.wiff (Turbo Spra...
Max. 2.5e7 cps.
Intensity, cps
Time, min
0.82
0.63
1.17
2.01
2.58
2.97
3.91
5.69
6.17
7.76
8.83
9.66

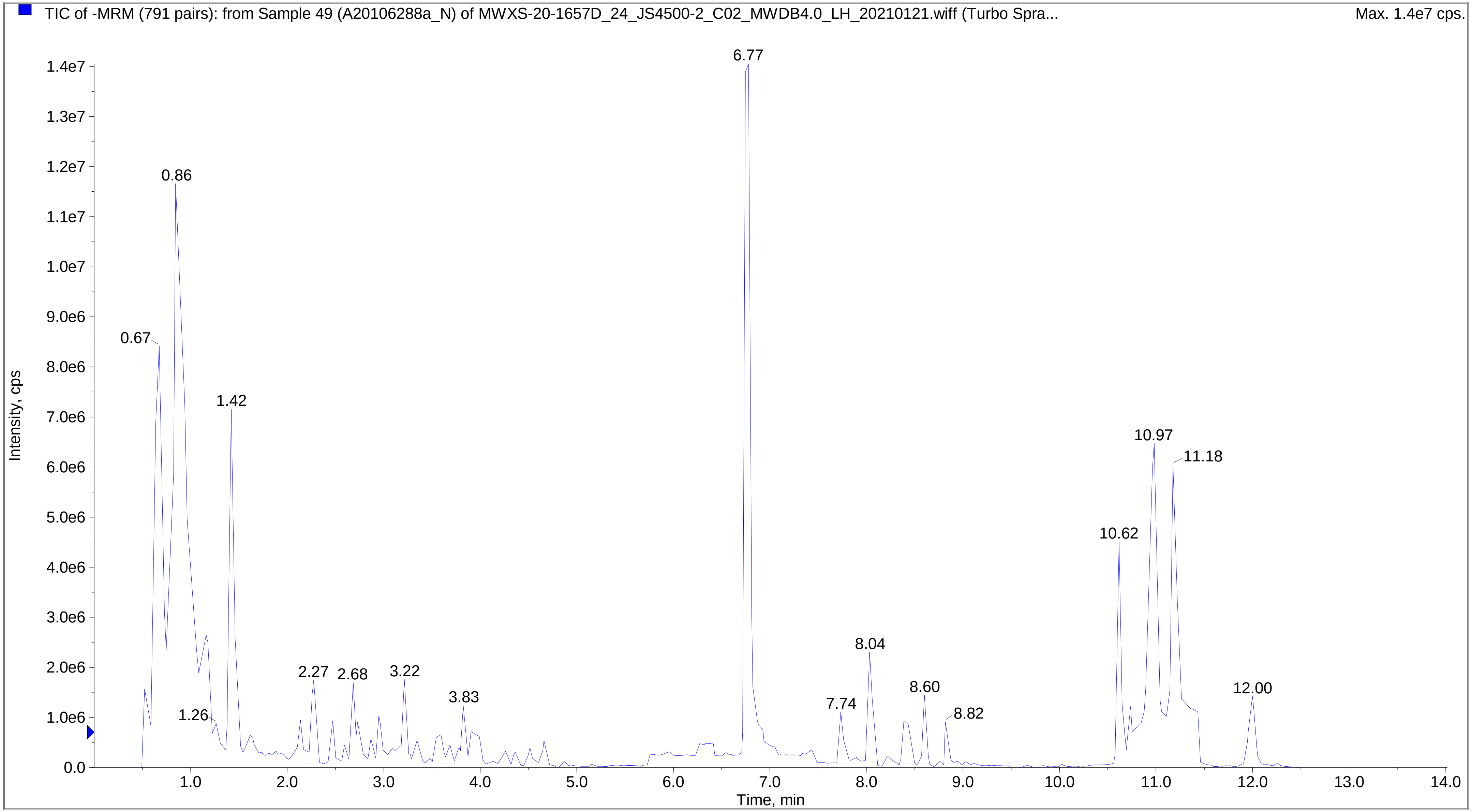
TIC of -MRM (791 pairs): from Sample 49 (A20106288a_N) of MWXS-20-1657D_24_JS4500-2_C02_MWDB4.0_LH_20210121.wiff (Turbo Spra...
Max. 1.4e7 cps.
Intensity, cps
Time, min
6.77
0.86
0.67
1.42
1.26
2.27
2.68
3.22
3.83
7.74
8.04
8.60
8.82
10.62
10.97
11.18
12.00

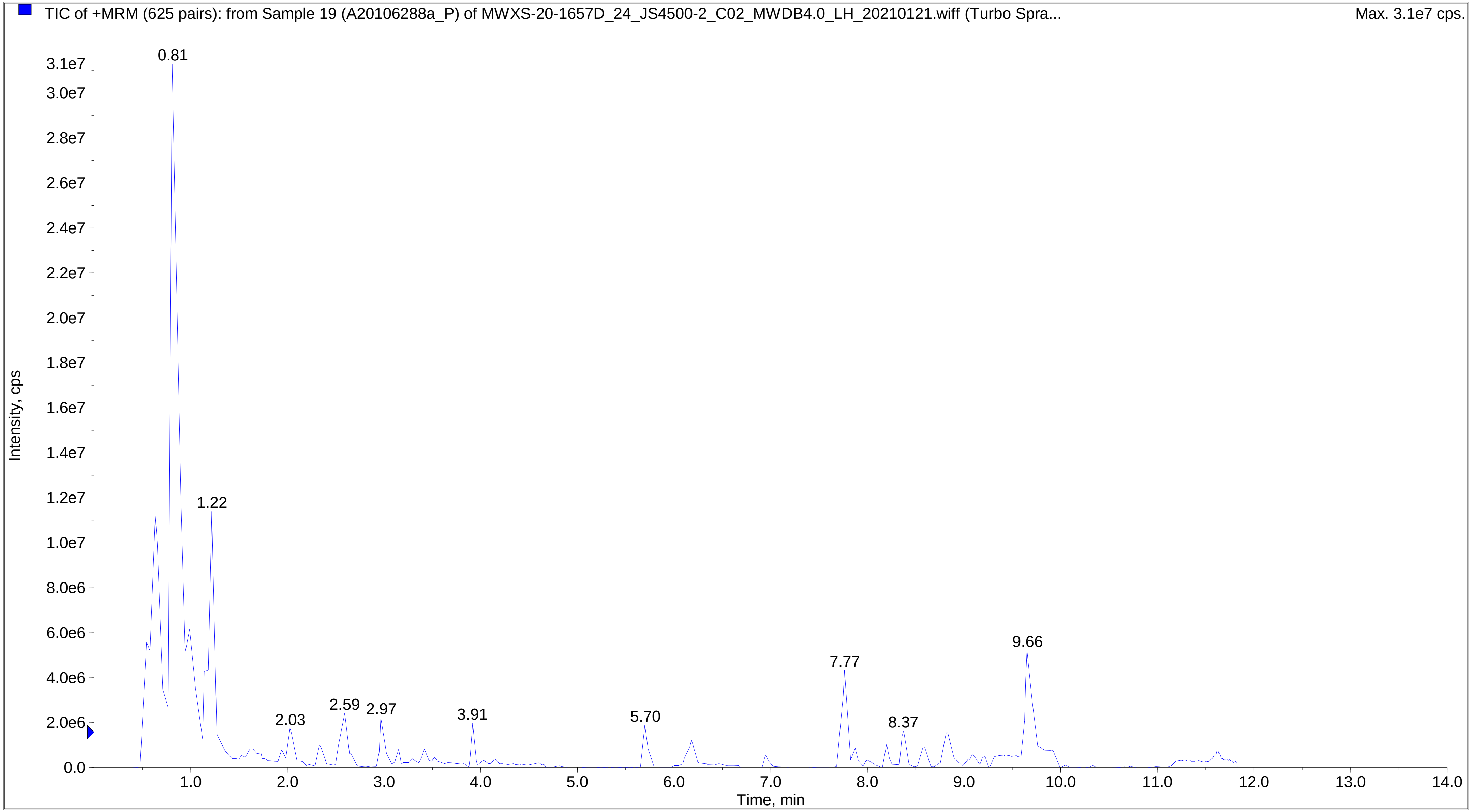

TIC of +MRM (625 pairs): from Sample 19 (A20106288a_P) of MWXS-20-1657D_24_JS4500-2_C02_MWDB4.0_LH_20210121.wiff (Turbo Spra...
Max. 3.1e7 cps.
Intensity, cps
Time, min
0.81
1.22
2.03
2.59
2.97
3.91
5.70
7.77
8.37
9.66
3.1e7
3.0e7
2.8e7
2.6e7
2.4e7
2.2e7
2.0e7
1.8e7
1.6e7
1.4e7
1.2e7
1.0e7
8.0e6
6.0e6
4.0e6
2.0e6
0.0
1.0
2.0
3.0
4.0
5.0
6.0
7.0
8.0
9.0
10.0
11.0
12.0
13.0
14.0

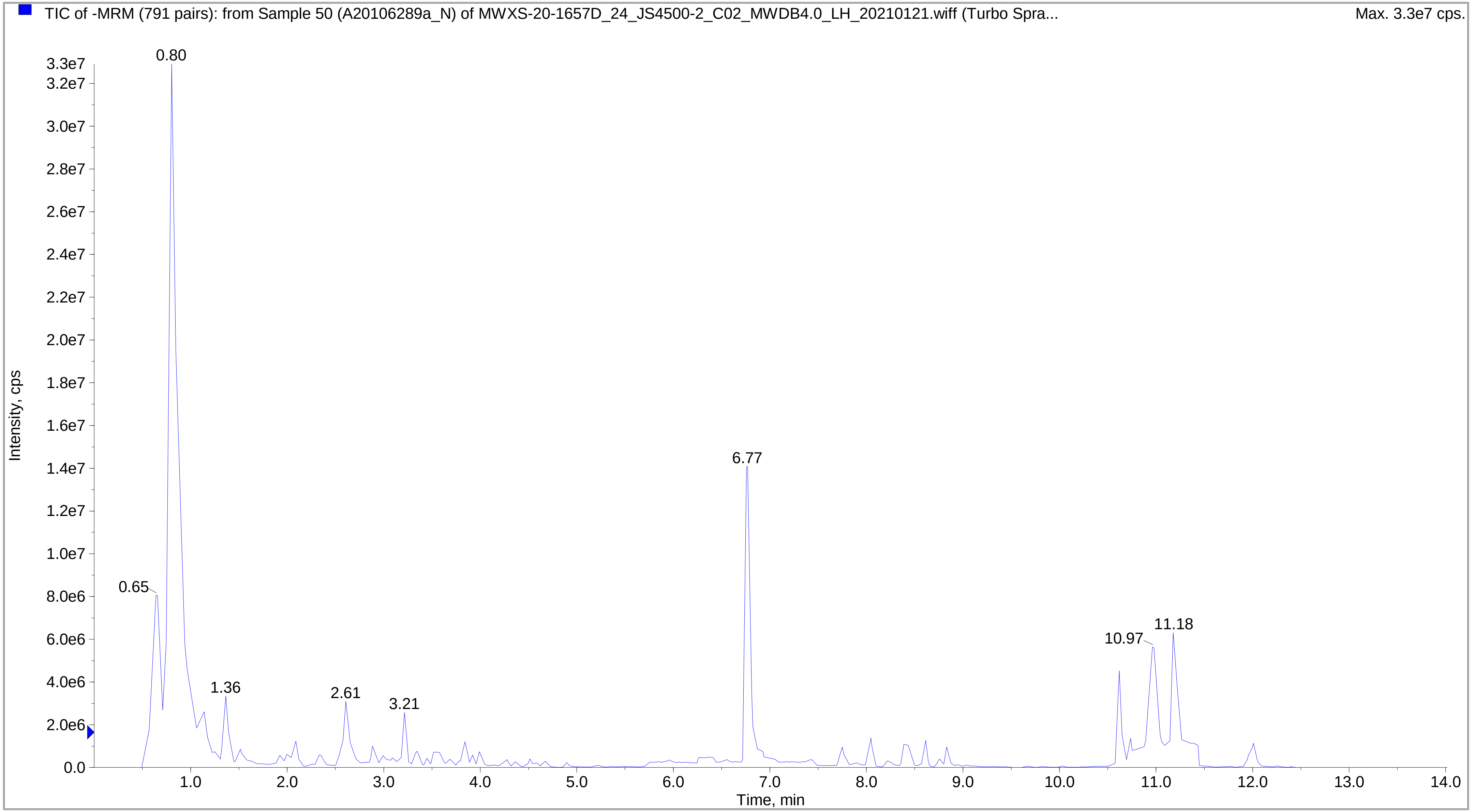

TIC of -MRM (791 pairs): from Sample 50 (A20106289a_N) of MWXS-20-1657D_24_JS4500-2_C02_MWDB4.0_LH_20210121.wiff (Turbo Spra...
Max. 3.3e7 cps.
0.80
0.65
1.36
2.61
3.21
6.77
10.97
11.18
3.3e7
3.2e7
3.0e7
2.8e7
2.6e7
2.4e7
2.2e7
2.0e7
1.8e7
1.6e7
1.4e7
1.2e7
1.0e7
8.0e6
6.0e6
4.0e6
2.0e6
0.0
Intensity, cps
1.0
2.0
3.0
4.0
5.0
6.0
7.0
8.0
9.0
10.0
11.0
12.0
13.0
14.0
Time, min

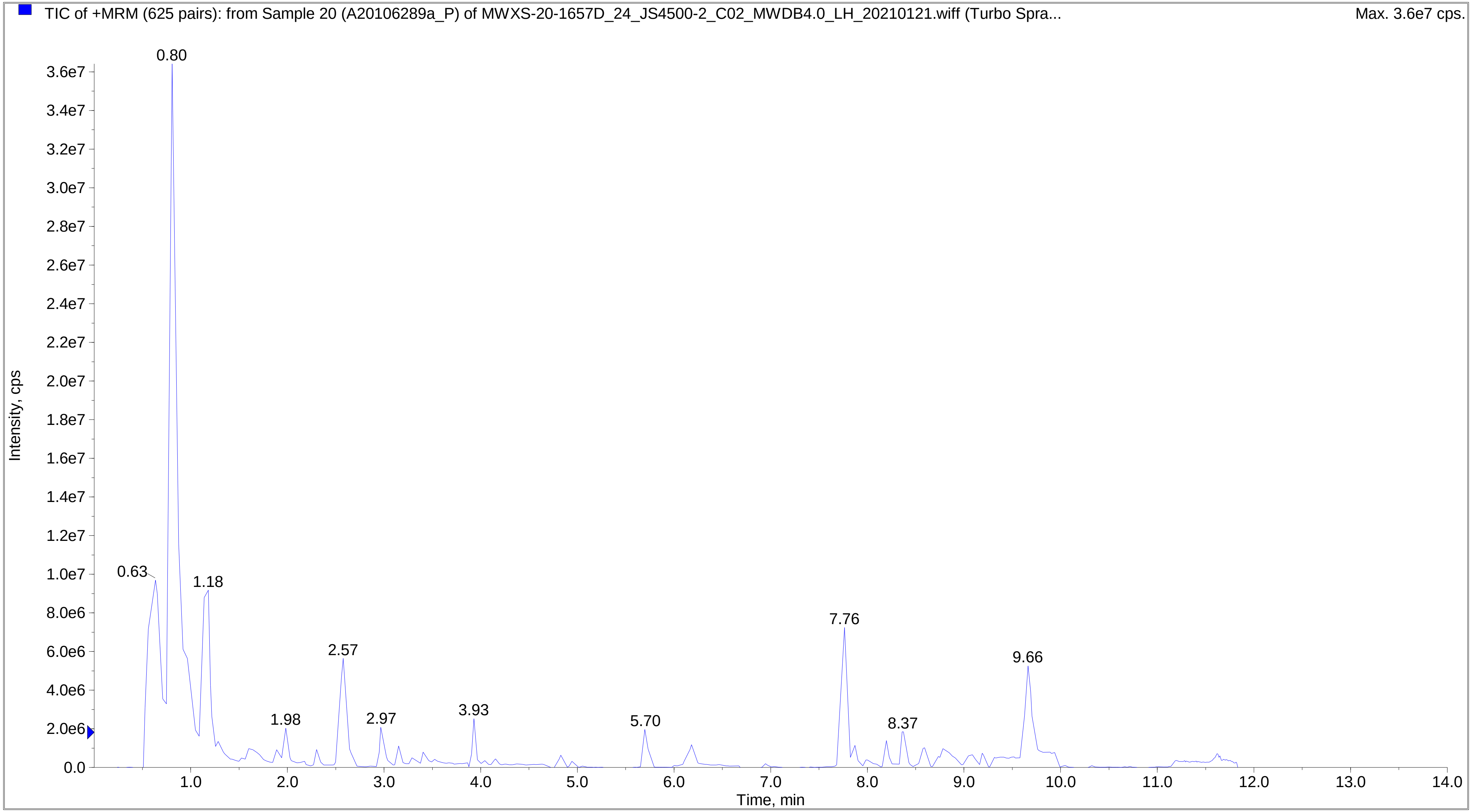
TIC of +MRM (625 pairs): from Sample 20 (A20106289a_P) of MWXS-20-1657D_24_JS4500-2_C02_MWDB4.0_LH_20210121.wiff (Turbo Spra...
Max. 3.6e7 cps.
Intensity, cps
Time, min
0.80
0.63
1.18
1.98
2.57
2.97
3.93
5.70
7.76
8.37
9.66

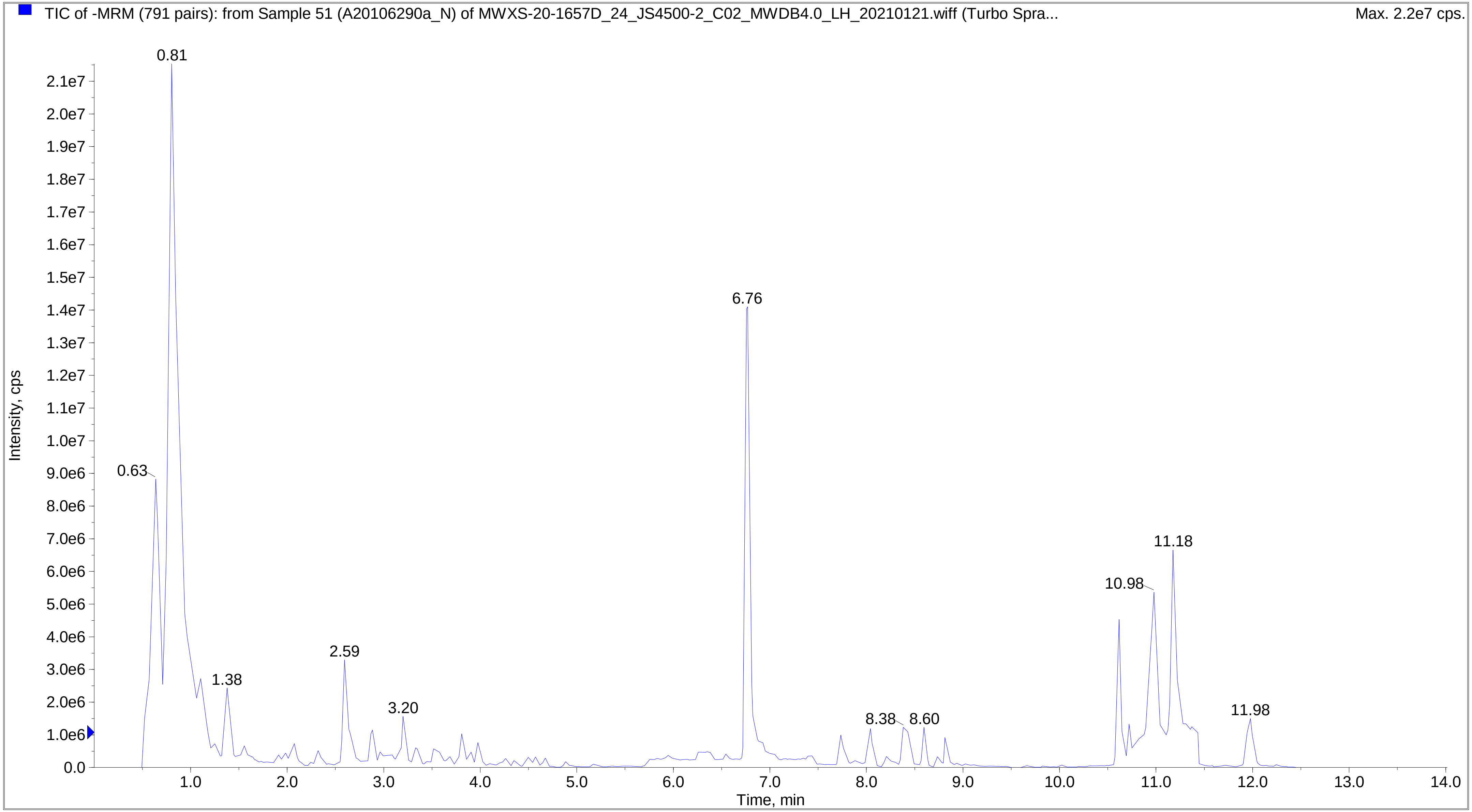

TIC of -MRM (791 pairs): from Sample 51 (A20106290a_N) of MWXS-20-1657D_24_JS4500-2_C02_MWDB4.0_LH_20210121.wiff (Turbo Spra...
Max. 2.2e7 cps.
Intensity, cps
Time, min
0.81
0.63
1.38
2.59
3.20
6.76
8.38
8.60
10.98
11.18
11.98

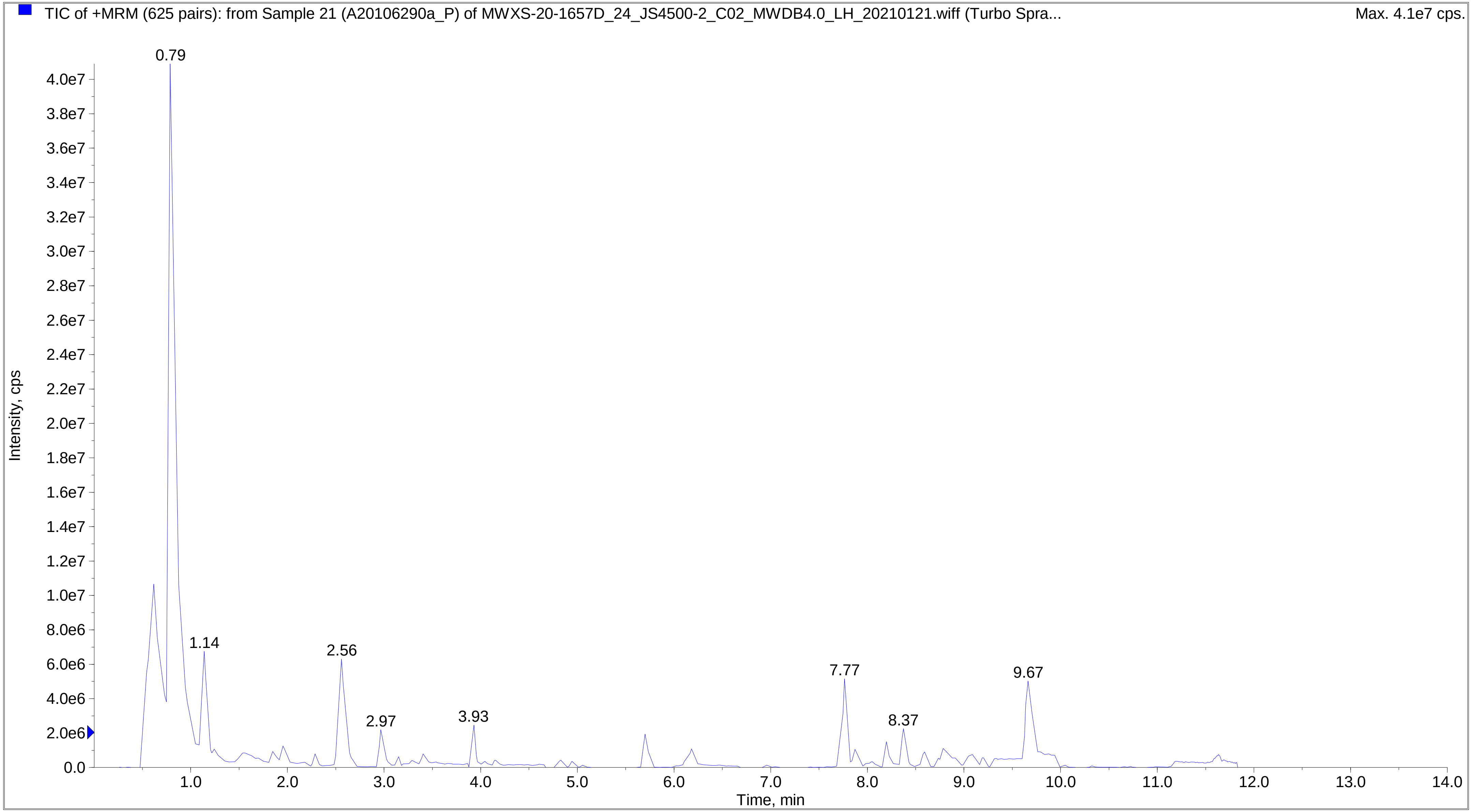

TIC of +MRM (625 pairs): from Sample 21 (A20106290a_P) of MWXS-20-1657D_24_JS4500-2_C02_MWDB4.0_LH_20210121.wiff (Turbo Spra...
Max. 4.1e7 cps.
0.79
1.14
2.56
2.97
3.93
7.77
8.37
9.67
4.0e7
3.8e7
3.6e7
3.4e7
3.2e7
3.0e7
2.8e7
2.6e7
2.4e7
2.2e7
2.0e7
1.8e7
1.6e7
1.4e7
1.2e7
1.0e7
8.0e6
6.0e6
4.0e6
2.0e6
0.0
Intensity, cps
1.0
2.0
3.0
4.0
5.0
6.0
7.0
8.0
9.0
10.0
11.0
12.0
13.0
14.0
Time, min

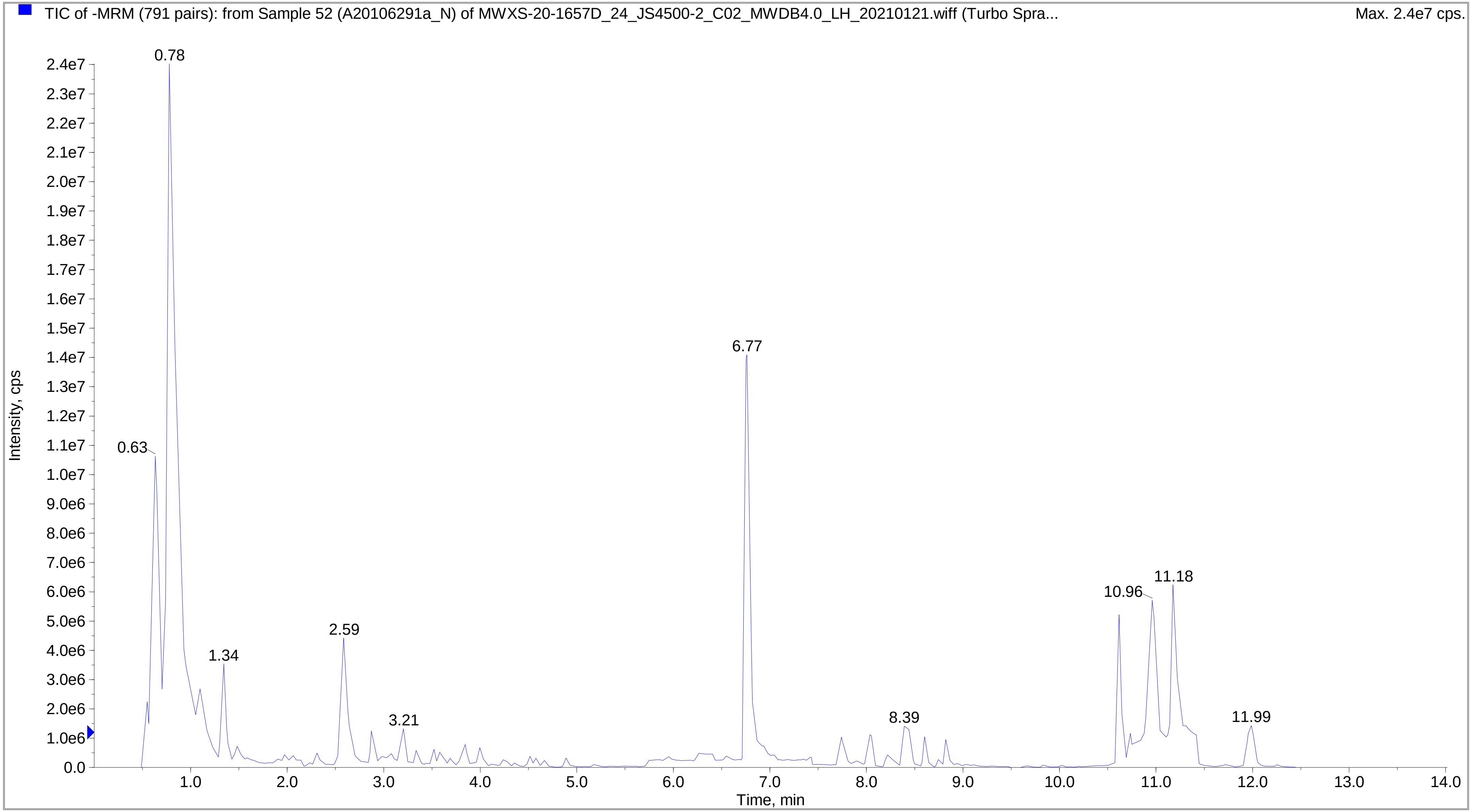
TIC of -MRM (791 pairs): from Sample 52 (A20106291a_N) of MWXS-20-1657D_24_JS4500-2_C02_MWDB4.0_LH_20210121.wiff (Turbo Spra...
Max. 2.4e7 cps.
Intensity, cps
Time, min
0.78
0.63
1.34
2.59
3.21
6.77
8.39
10.96
11.18
11.99
2.4e7
2.3e7
2.2e7
2.1e7
2.0e7
1.9e7
1.8e7
1.7e7
1.6e7
1.5e7
1.4e7
1.3e7
1.2e7
1.1e7
1.0e7
9.0e6
8.0e6
7.0e6
6.0e6
5.0e6
4.0e6
3.0e6
2.0e6
1.0e6
0.0
1.0
2.0
3.0
4.0
5.0
6.0
7.0
8.0
9.0
10.0
11.0
12.0
13.0
14.0

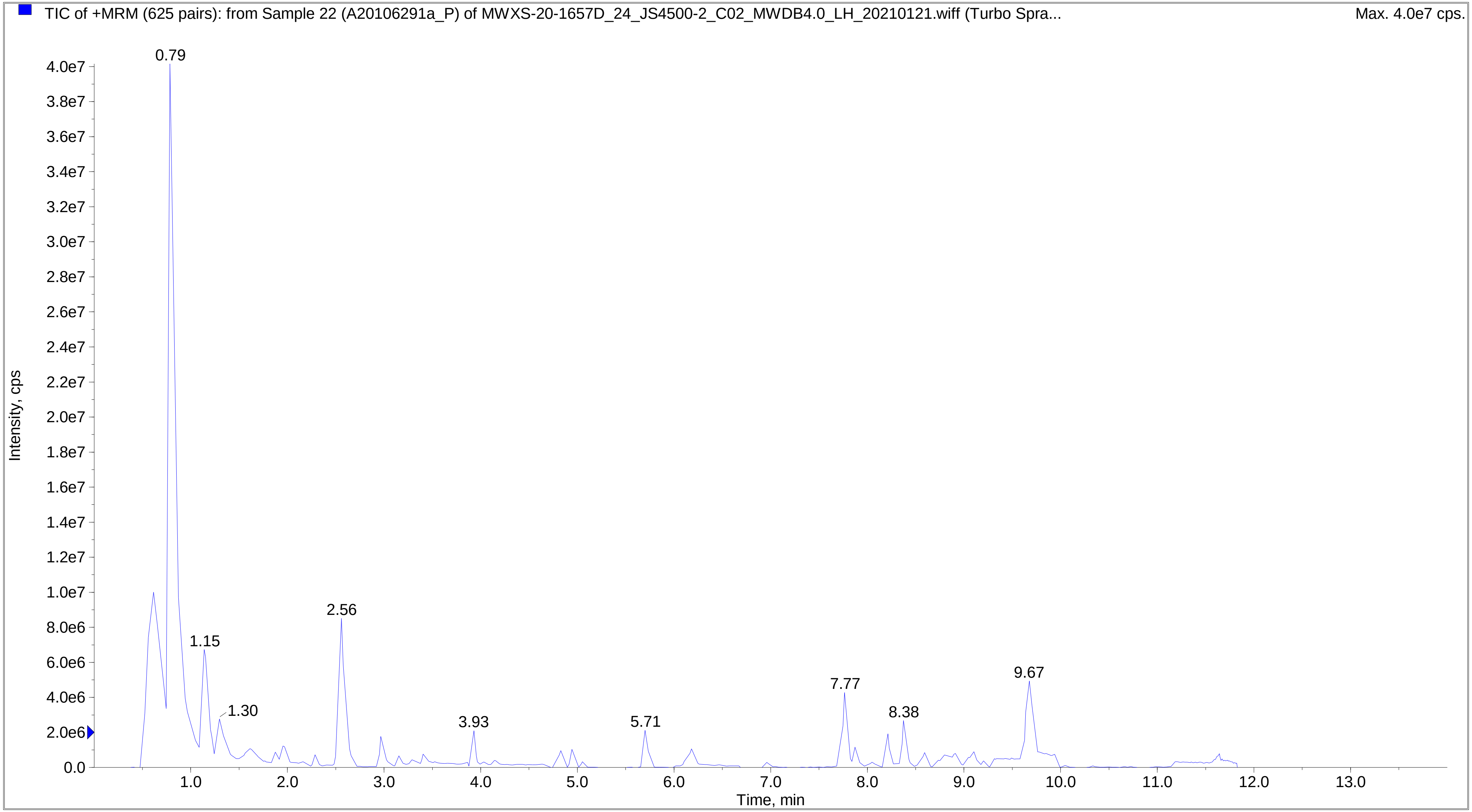

TIC of +MRM (625 pairs): from Sample 22 (A20106291a_P) of MWXS-20-1657D_24_JS4500-2_C02_MWDB4.0_LH_20210121.wiff (Turbo Spra...
Max. 4.0e7 cps.
Intensity, cps
Time, min
0.79
1.15
1.30
2.56
3.93
5.71
7.77
8.38
9.67
4.0e7
3.8e7
3.6e7
3.4e7
3.2e7
3.0e7
2.8e7
2.6e7
2.4e7
2.2e7
2.0e7
1.8e7
1.6e7
1.4e7
1.2e7
1.0e7
8.0e6
6.0e6
4.0e6
2.0e6
0.0
1.0
2.0
3.0
4.0
5.0
6.0
7.0
8.0
9.0
10.0
11.0
12.0
13.0

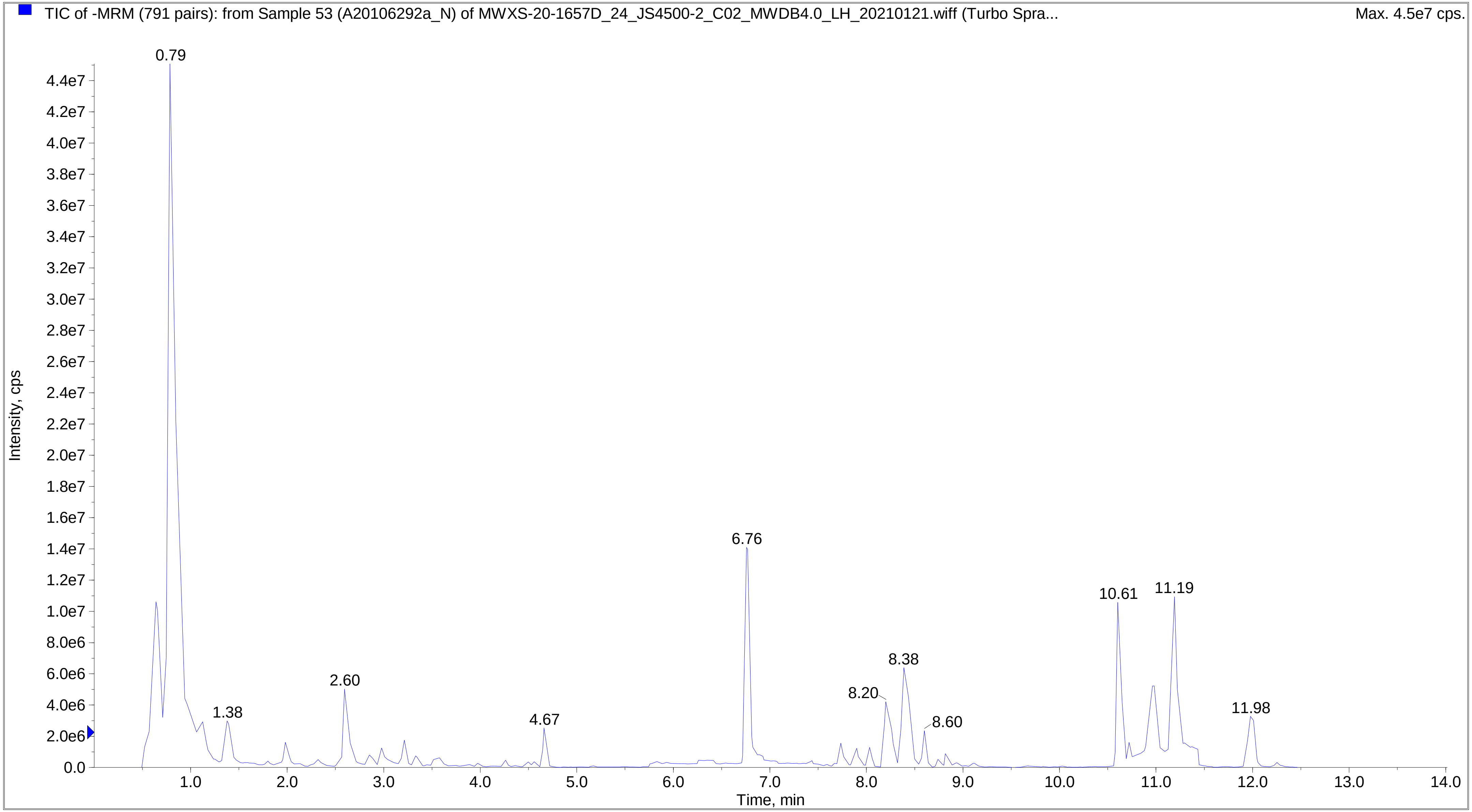

TIC of -MRM (791 pairs): from Sample 53 (A20106292a_N) of MWXS-20-1657D_24_JS4500-2_C02_MWDB4.0_LH_20210121.wiff (Turbo Spra...
Max. 4.5e7 cps.
Intensity, cps
Time, min
0.79
1.38
2.60
4.67
6.76
8.20
8.38
8.60
10.61
11.19
11.98

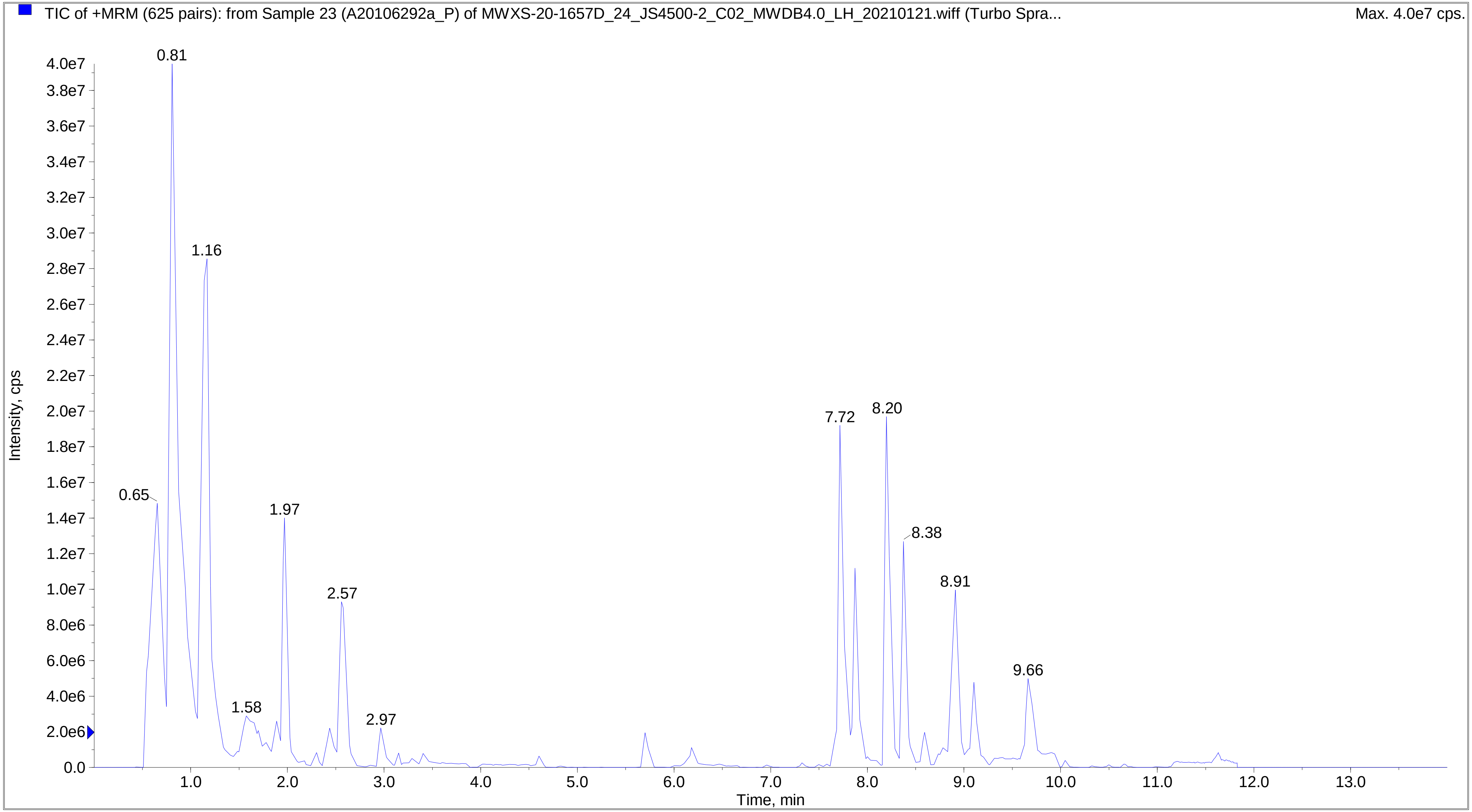
TIC of +MRM (625 pairs): from Sample 23 (A20106292a_P) of MWXS-20-1657D_24_JS4500-2_C02_MWDB4.0_LH_20210121.wiff (Turbo Spra...
Max. 4.0e7 cps.
Intensity, cps
Time, min
0.81
1.16
0.65
1.97
2.57
1.58
2.97
7.72
8.20
8.38
8.91
9.66
4.0e7
3.8e7
3.6e7
3.4e7
3.2e7
3.0e7
2.8e7
2.6e7
2.4e7
2.2e7
2.0e7
1.8e7
1.6e7
1.4e7
1.2e7
1.0e7
8.0e6
6.0e6
4.0e6
2.0e6
0.0
1.0
2.0
3.0
4.0
5.0
6.0
7.0
8.0
9.0
10.0
11.0
12.0
13.0

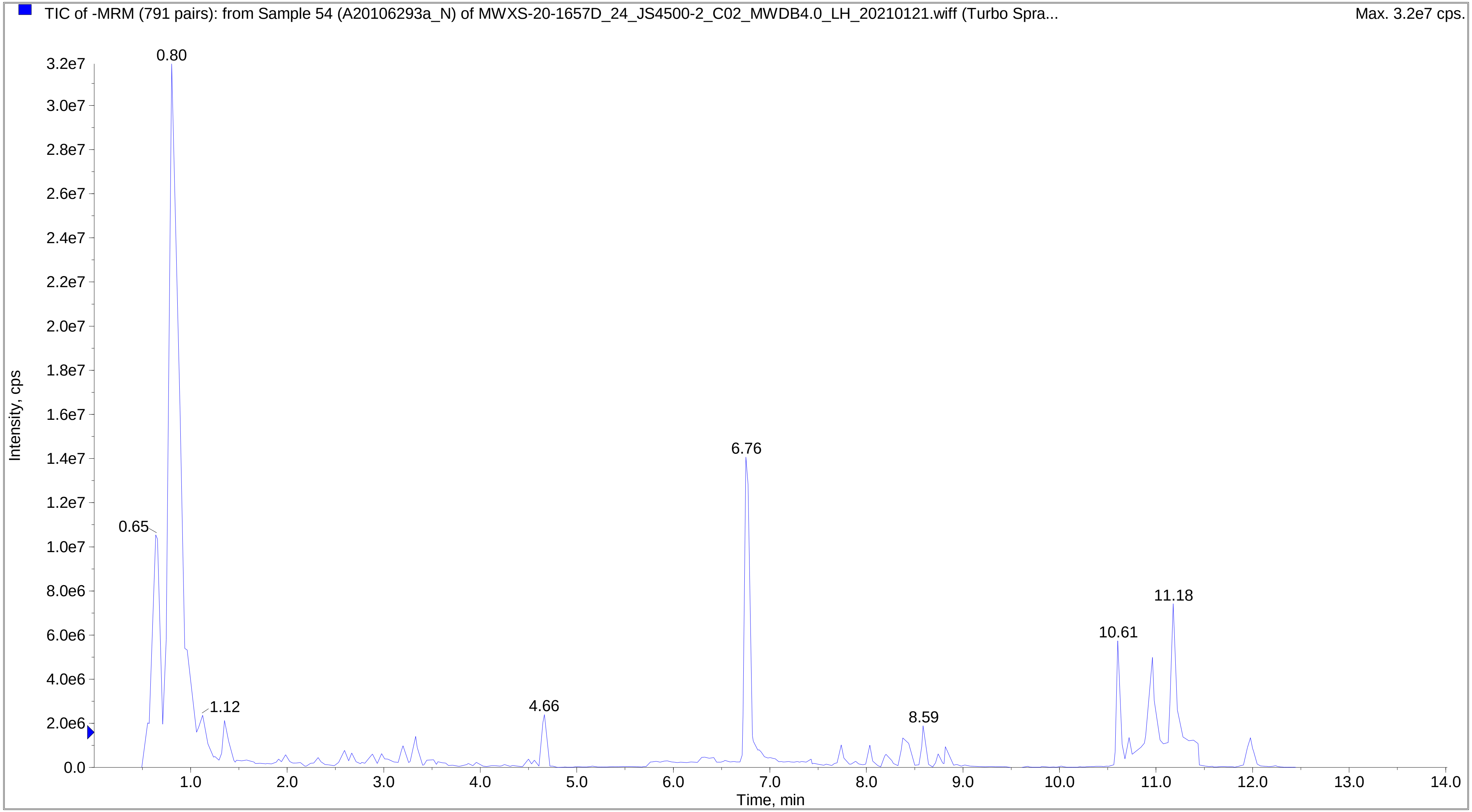

TIC of -MRM (791 pairs): from Sample 54 (A20106293a_N) of MWXS-20-1657D_24_JS4500-2_C02_MWDB4.0_LH_20210121.wiff (Turbo Spra...
Max. 3.2e7 cps.
0.80
0.65
1.12
4.66
6.76
8.59
10.61
11.18
Intensity, cps
3.2e7
3.0e7
2.8e7
2.6e7
2.4e7
2.2e7
2.0e7
1.8e7
1.6e7
1.4e7
1.2e7
1.0e7
8.0e6
6.0e6
4.0e6
2.0e6
0.0
1.0
2.0
3.0
4.0
5.0
6.0
7.0
8.0
9.0
10.0
11.0
12.0
13.0
14.0
Time, min

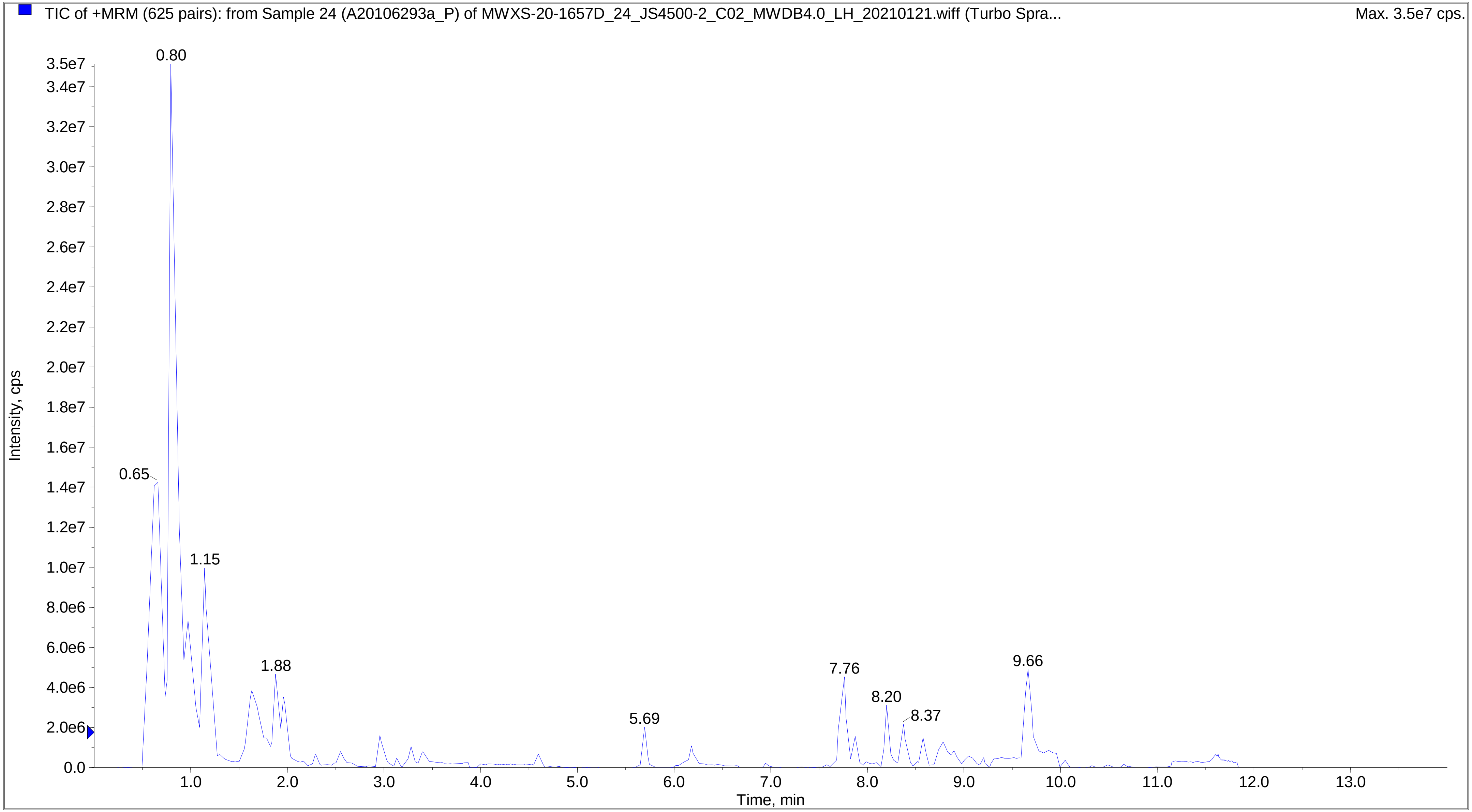
TIC of +MRM (625 pairs): from Sample 24 (A20106293a_P) of MWXS-20-1657D_24_JS4500-2_C02_MWDB4.0_LH_20210121.wiff (Turbo Spra...
Max. 3.5e7 cps.
Intensity, cps
Time, min
0.80
0.65
1.15
1.88
5.69
7.76
8.20
8.37
9.66

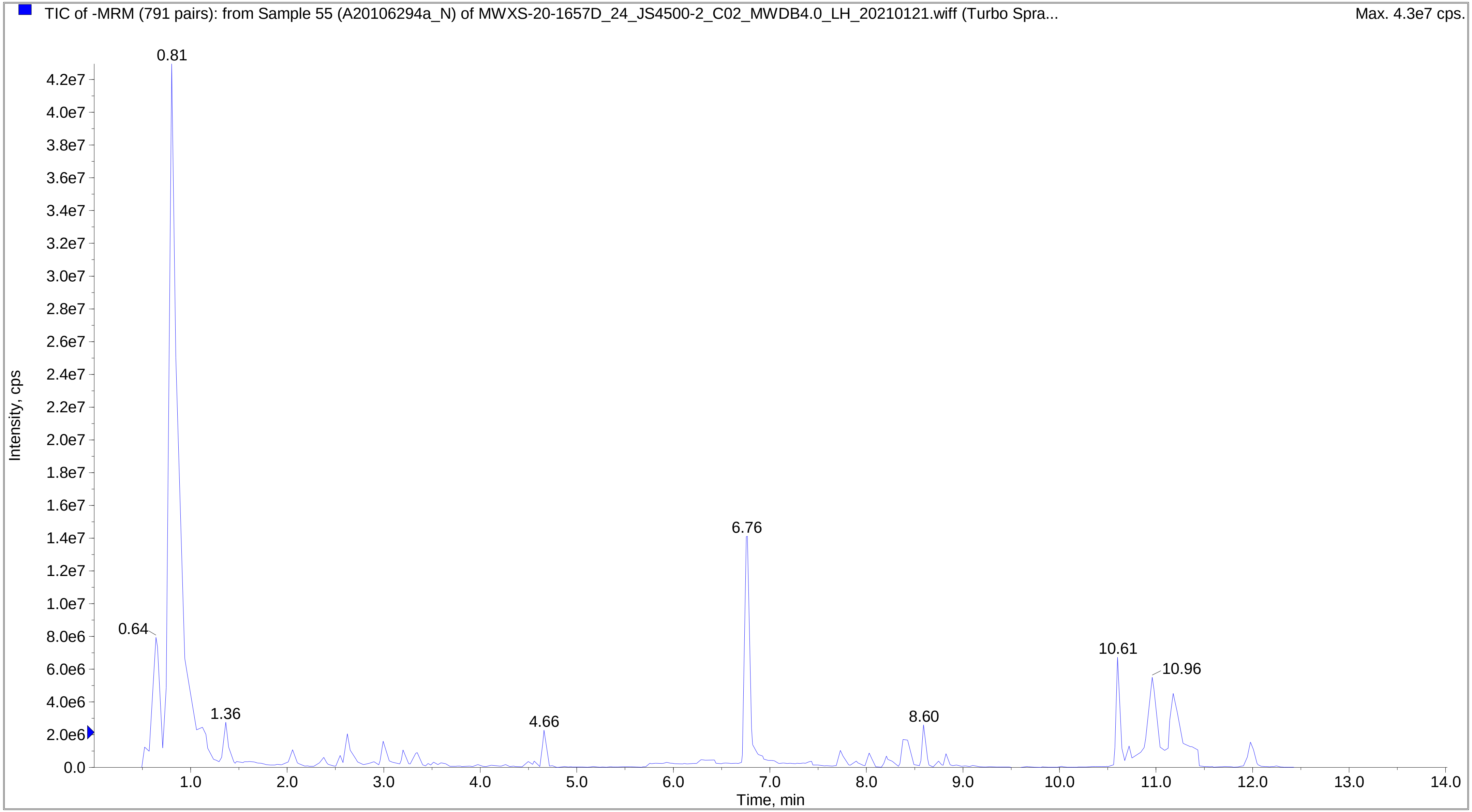

TIC of -MRM (791 pairs): from Sample 55 (A20106294a_N) of MWXS-20-1657D_24_JS4500-2_C02_MWDB4.0_LH_20210121.wiff (Turbo Spra...
Max. 4.3e7 cps.
Intensity, cps
Time, min
0.81
0.64
1.36
4.66
6.76
8.60
10.61
10.96

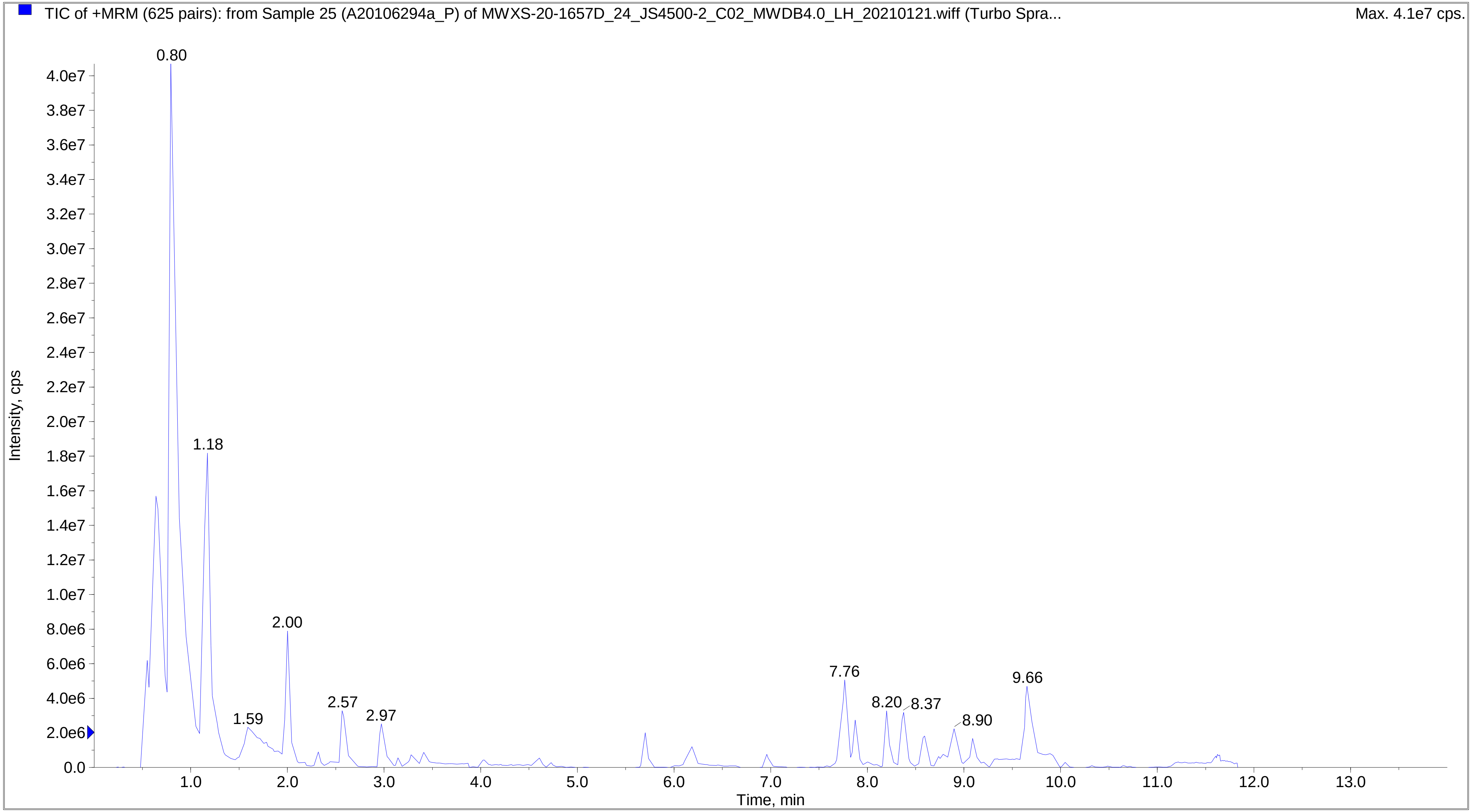

TIC of +MRM (625 pairs): from Sample 25 (A20106294a_P) of MWXS-20-1657D_24_JS4500-2_C02_MWDB4.0_LH_20210121.wiff (Turbo Spra...
Max. 4.1e7 cps.
Intensity, cps
Time, min
0.80
1.18
1.59
2.00
2.57
2.97
7.76
8.20
8.37
8.90
9.66

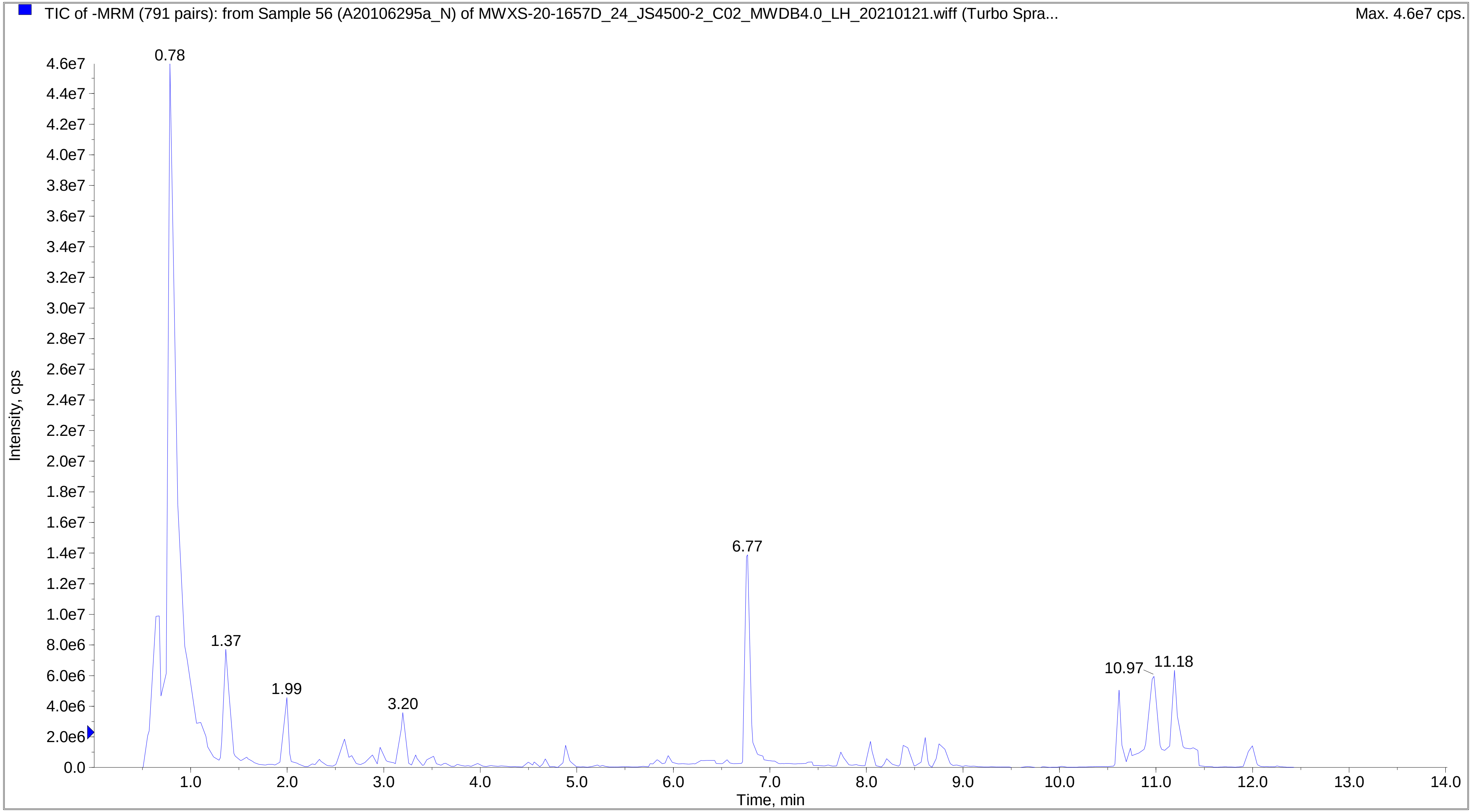
TIC of -MRM (791 pairs): from Sample 56 (A20106295a_N) of MWXS-20-1657D_24_JS4500-2_C02_MWDB4.0_LH_20210121.wiff (Turbo Spra...
Max. 4.6e7 cps.
Intensity, cps
Time, min
0.78
1.37
1.99
3.20
6.77
10.97
11.18

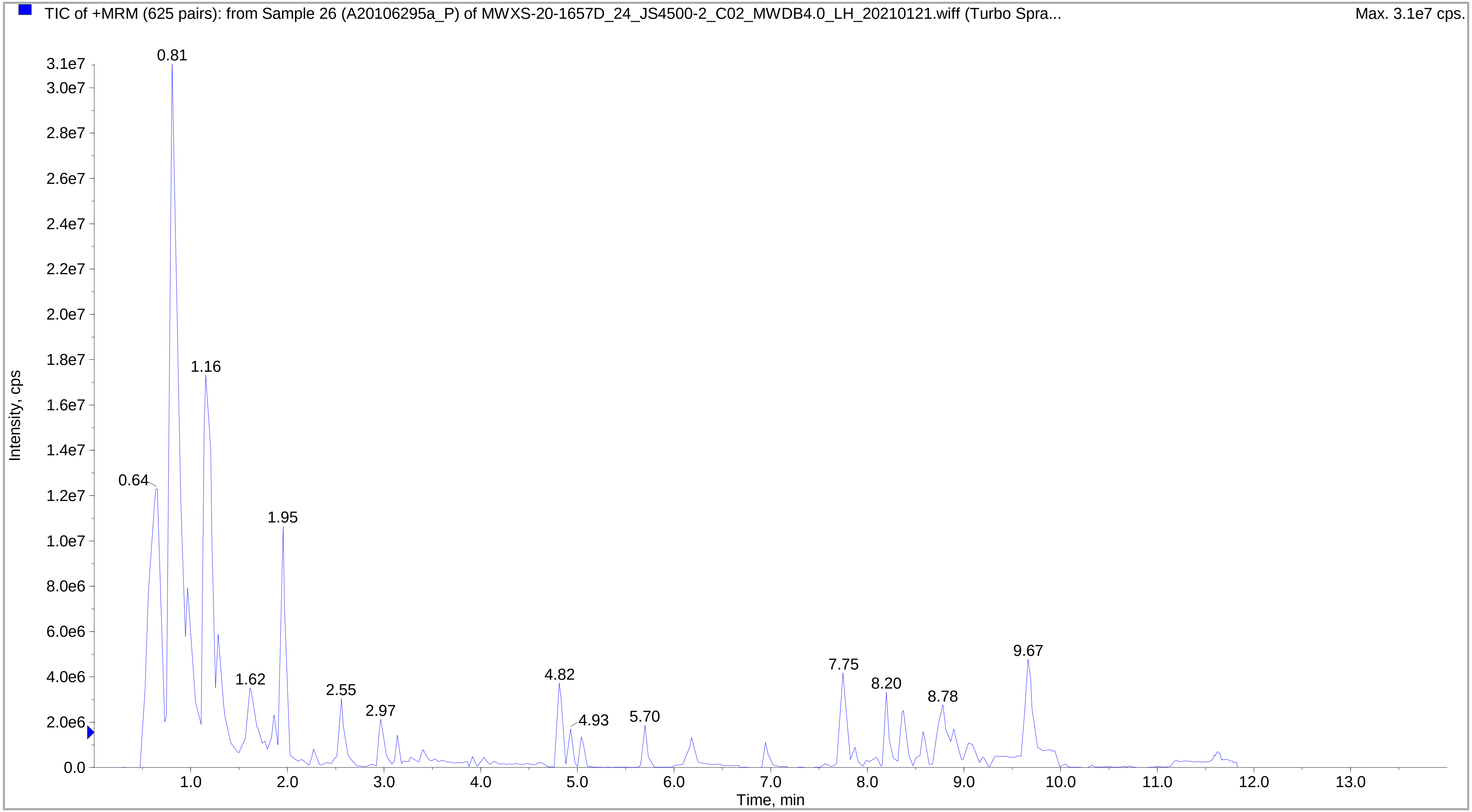

TIC of +MRM (625 pairs): from Sample 26 (A20106295a_P) of MWXS-20-1657D_24_JS4500-2_C02_MWDB4.0_LH_20210121.wiff (Turbo Spra...
Max. 3.1e7 cps.
Intensity, cps
Time, min
0.81
1.16
0.64
1.95
1.62
2.55
2.97
4.82
4.93
5.70
7.75
8.20
8.78
9.67

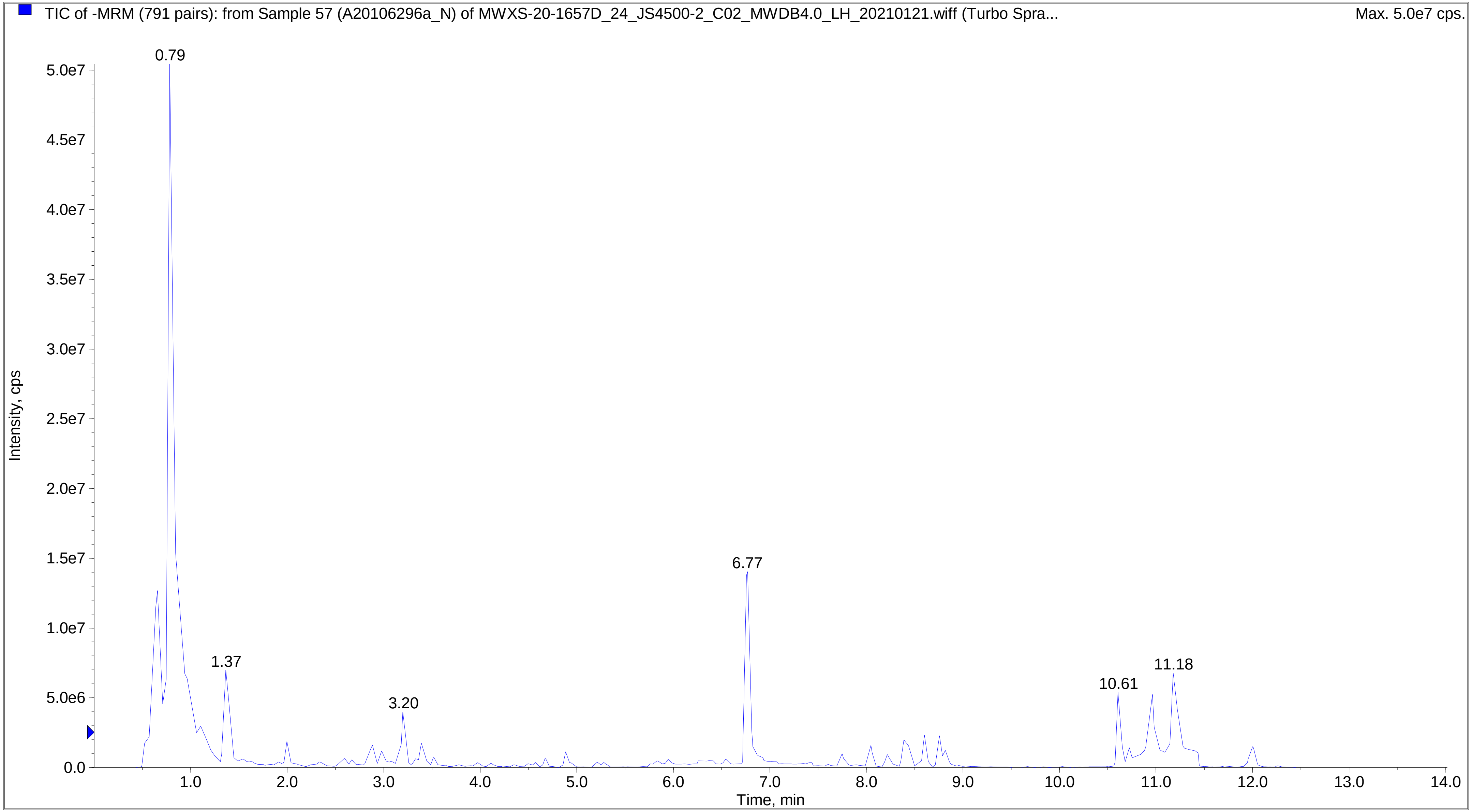
TIC of -MRM (791 pairs): from Sample 57 (A20106296a_N) of MWXS-20-1657D_24_JS4500-2_C02_MWDB4.0_LH_20210121.wiff (Turbo Spra...
Max. 5.0e7 cps.
0.79
1.37
3.20
6.77
10.61
11.18
Intensity, cps
Time, min
5.0e7
4.5e7
4.0e7
3.5e7
3.0e7
2.5e7
2.0e7
1.5e7
1.0e7
5.0e6
0.0
1.0
2.0
3.0
4.0
5.0
6.0
7.0
8.0
9.0
10.0
11.0
12.0
13.0
14.0

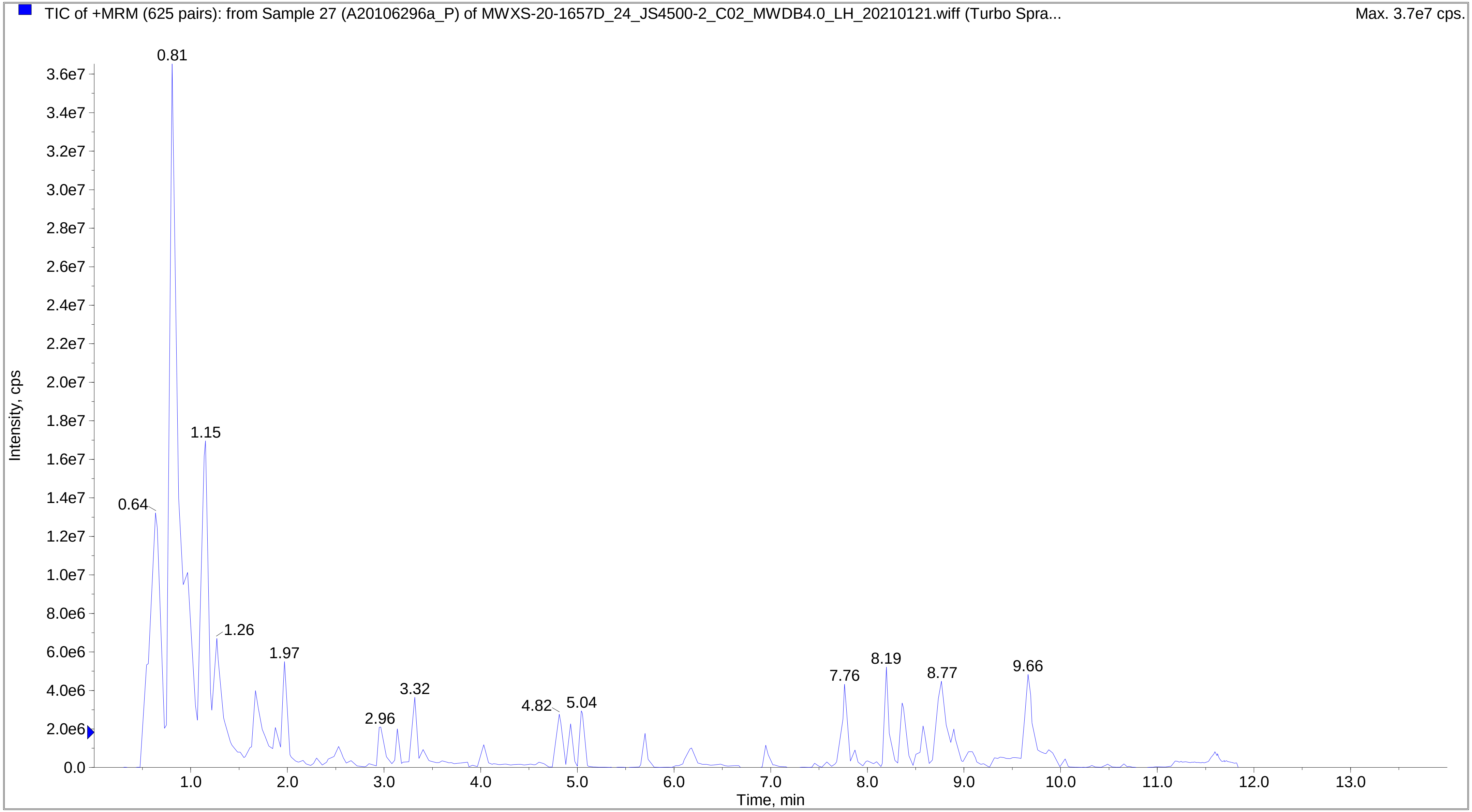

TIC of +MRM (625 pairs): from Sample 27 (A20106296a_P) of MWXS-20-1657D_24_JS4500-2_C02_MWDB4.0_LH_20210121.wiff (Turbo Spra...
Max. 3.7e7 cps.
Intensity, cps
Time, min
0.81
1.15
0.64
1.26
1.97
2.96
3.32
4.82
5.04
7.76
8.19
8.77
9.66

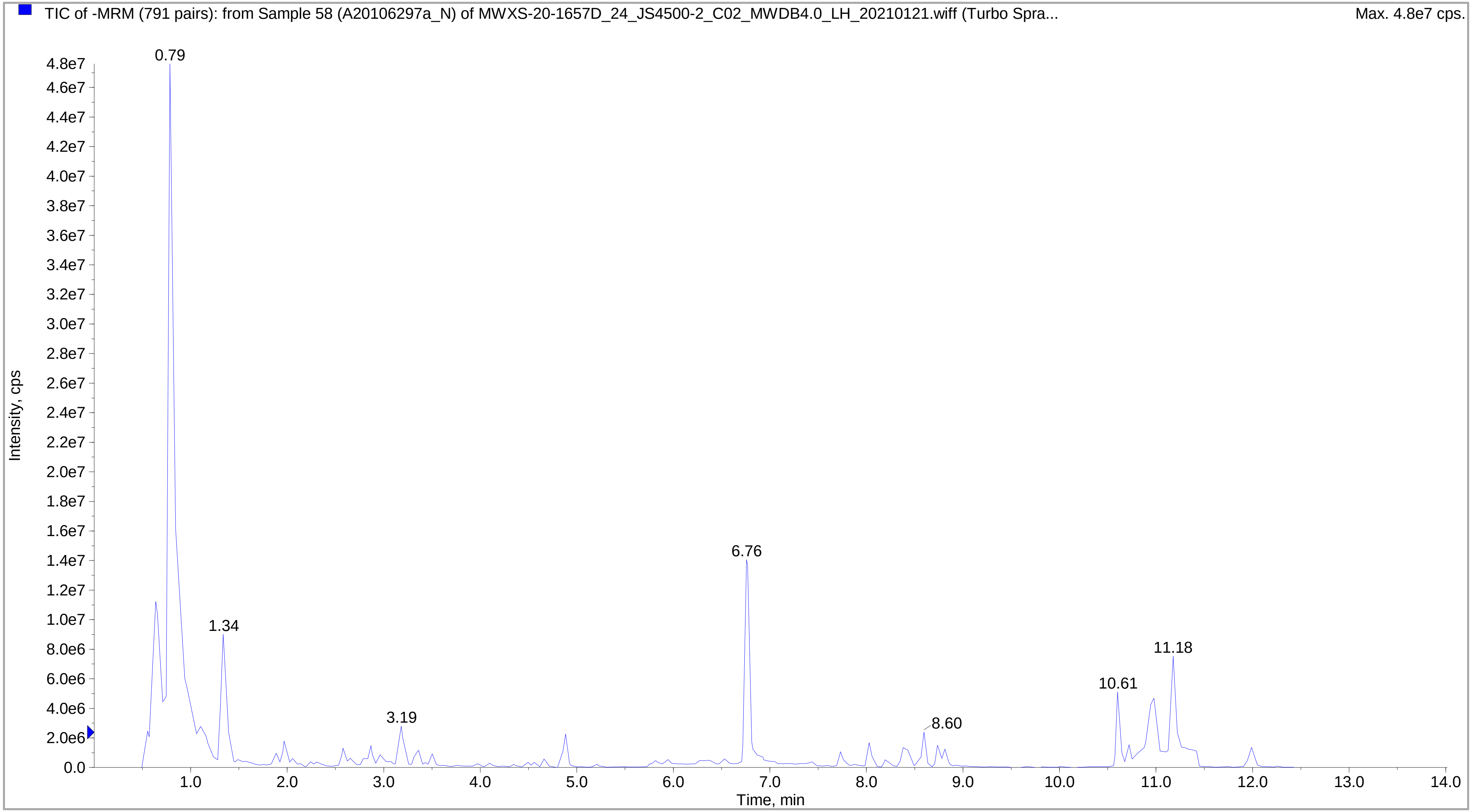

TIC of -MRM (791 pairs): from Sample 58 (A20106297a_N) of MWXS-20-1657D_24_JS4500-2_C02_MWDB4.0_LH_20210121.wiff (Turbo Spra...
Max. 4.8e7 cps.
Intensity, cps
Time, min
0.79
1.34
3.19
6.76
8.60
10.61
11.18

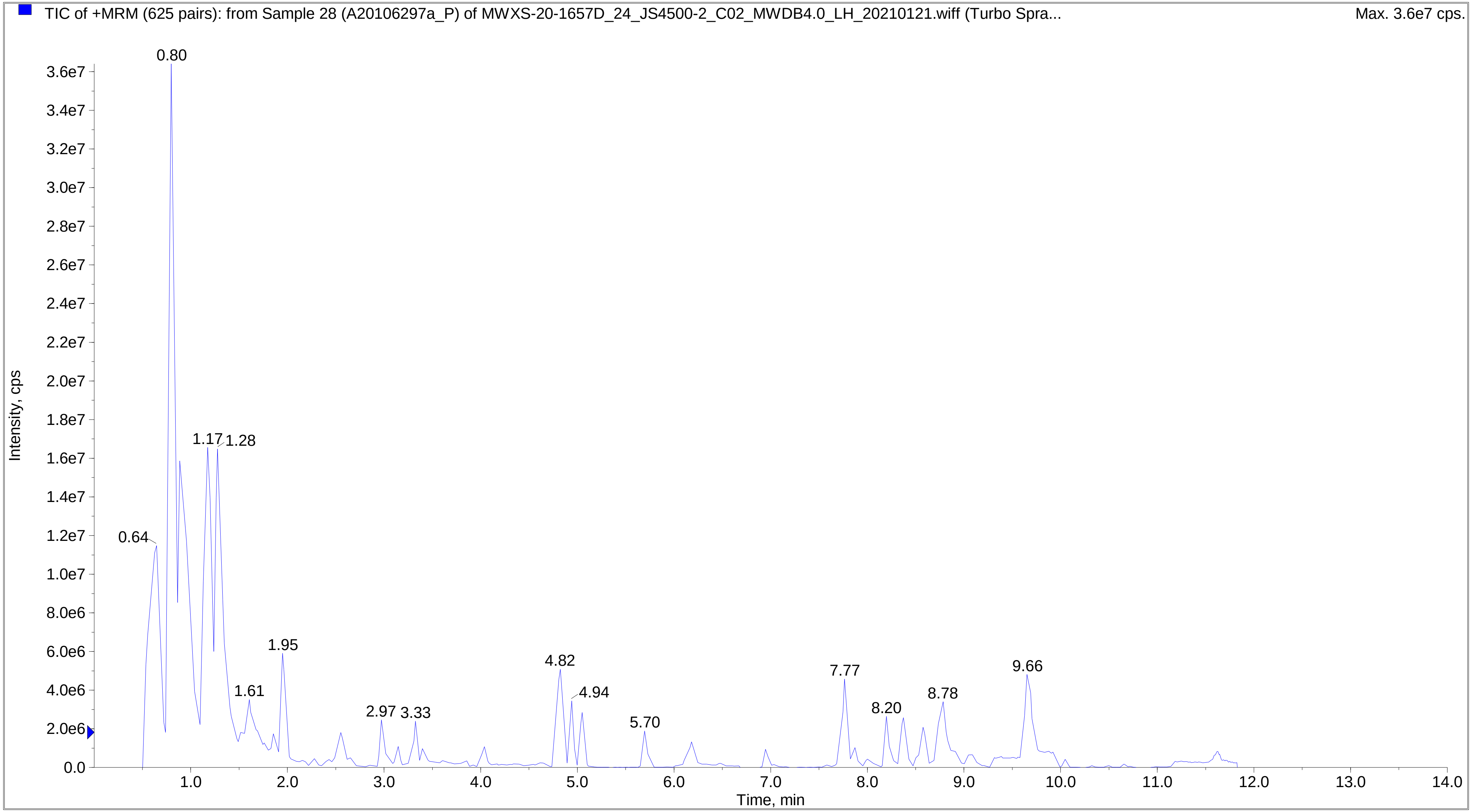

TIC of +MRM (625 pairs): from Sample 28 (A20106297a_P) of MWXS-20-1657D_24_JS4500-2_C02_MWDB4.0_LH_20210121.wiff (Turbo Spra...
Max. 3.6e7 cps.
Intensity, cps
Time, min
0.80
0.64
1.17
1.28
1.61
1.95
2.97
3.33
4.82
4.94
5.70
7.77
8.20
8.78
9.66

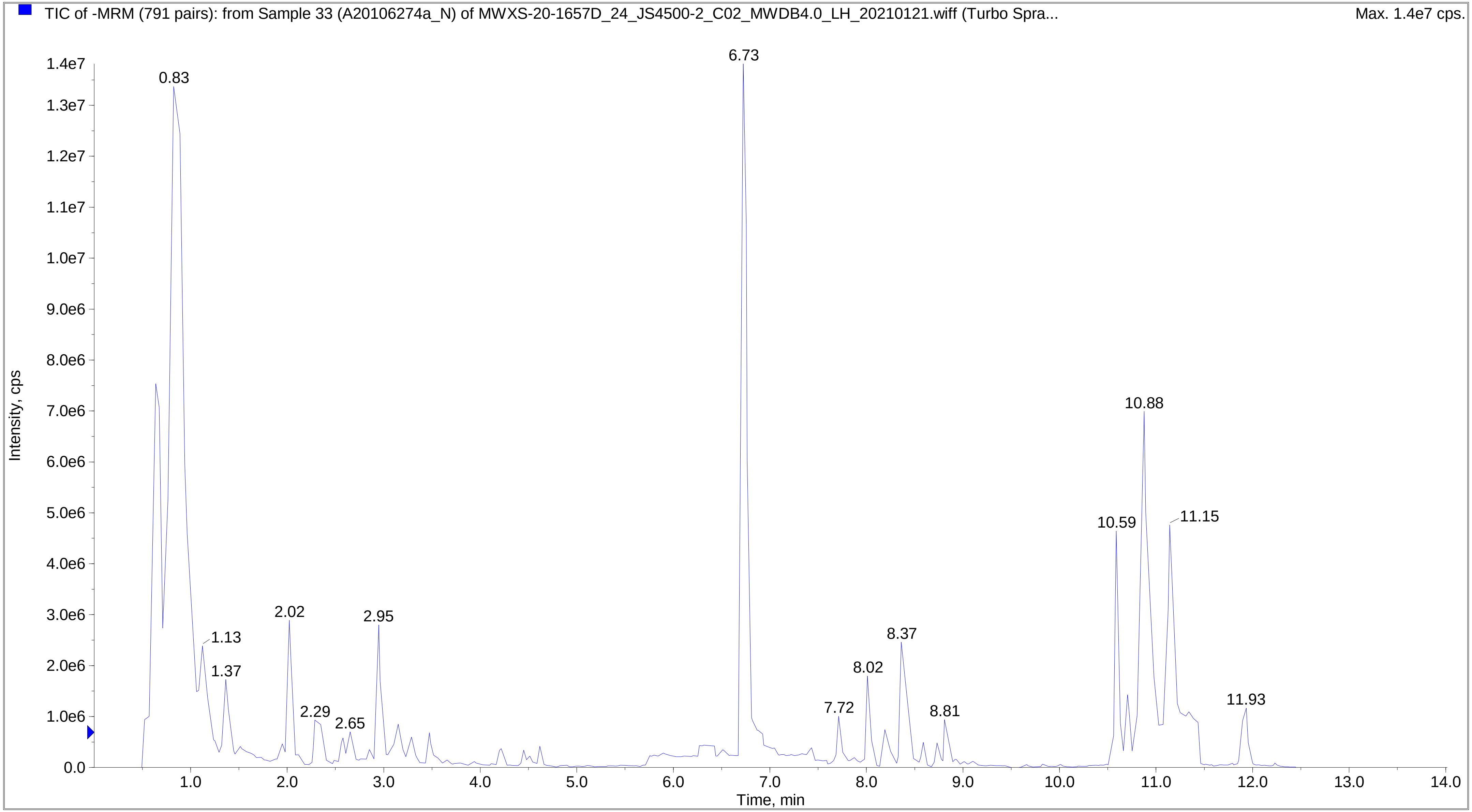
TIC of -MRM (791 pairs): from Sample 33 (A20106274a_N) of MWXS-20-1657D_24_JS4500-2_C02_MWDB4.0_LH_20210121.wiff (Turbo Spra...
Max. 1.4e7 cps.
Intensity, cps
Time, min
0.83
6.73
10.88
11.15
10.59
1.13
1.37
2.02
2.95
8.37
8.02
2.29
2.65
7.72
8.81
11.93
1.4e7
1.3e7
1.2e7
1.1e7
1.0e7
9.0e6
8.0e6
7.0e6
6.0e6
5.0e6
4.0e6
3.0e6
2.0e6
1.0e6
0.0
1.0
2.0
3.0
4.0
5.0
6.0
7.0
8.0
9.0
10.0
11.0
12.0
13.0
14.0

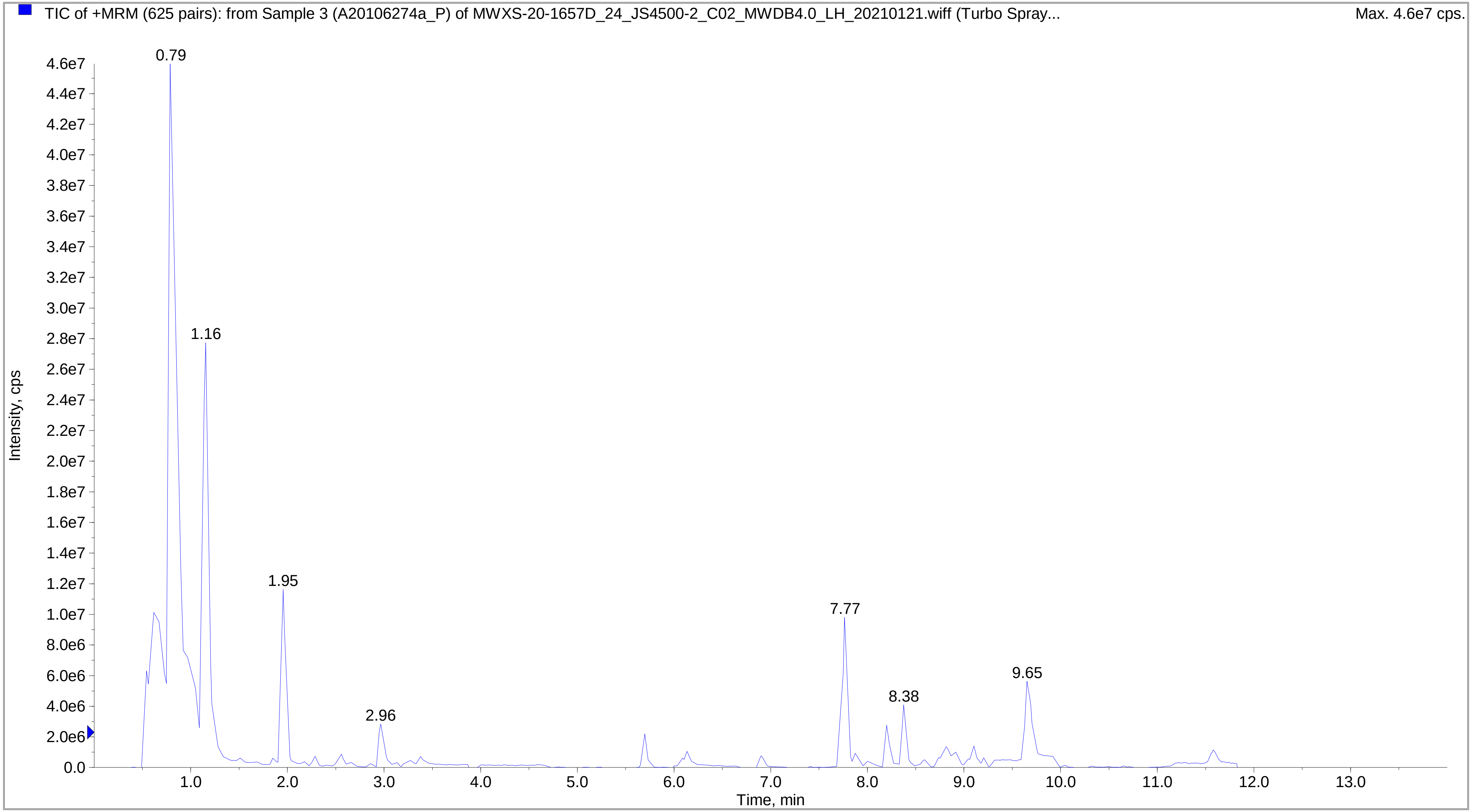
TIC of +MRM (625 pairs): from Sample 3 (A20106274a_P) of MWXS-20-1657D_24_JS4500-2_C02_MWDB4.0_LH_20210121.wiff (Turbo Spray...
Max. 4.6e7 cps.
Intensity, cps
Time, min
0.79
1.16
1.95
2.96
7.77
8.38
9.65

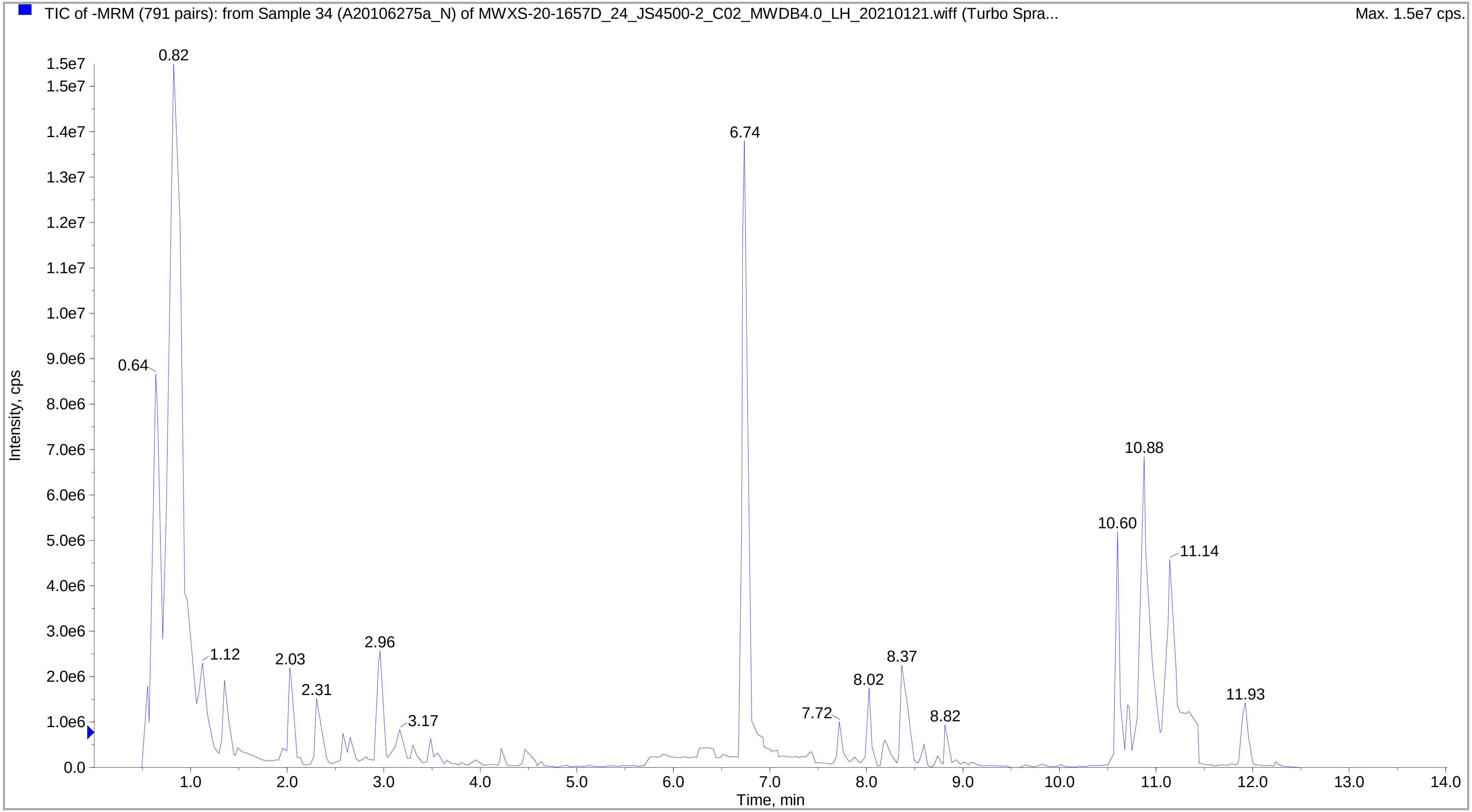

TIC of -MRM (791 pairs): from Sample 34 (A20106275a_N) of MWXS-20-1657D_24_JS4500-2_C02_MWDB4.0_LH_20210121.wiff (Turbo Spra...
Max. 1.5e7 cps.
Intensity, cps
Time, min
0.82
0.64
6.74
10.88
10.60
11.14
1.12
2.03
2.31
2.96
3.17
7.72
8.02
8.37
8.82
11.93

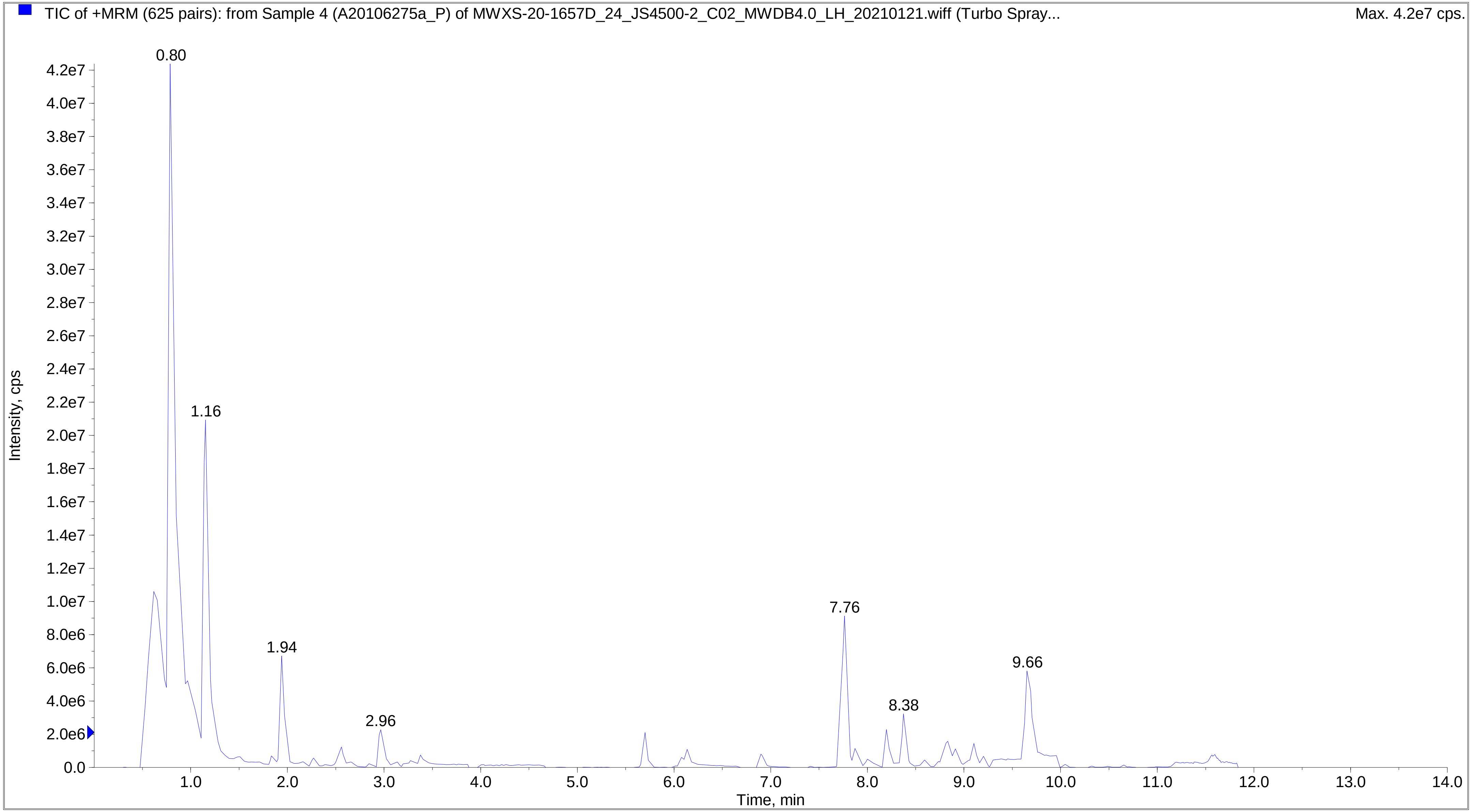
TIC of +MRM (625 pairs): from Sample 4 (A20106275a_P) of MWXS-20-1657D_24_JS4500-2_C02_MWDB4.0_LH_20210121.wiff (Turbo Spray...
Max. 4.2e7 cps.
Intensity, cps
Time, min
0.80
1.16
1.94
2.96
7.76
8.38
9.66

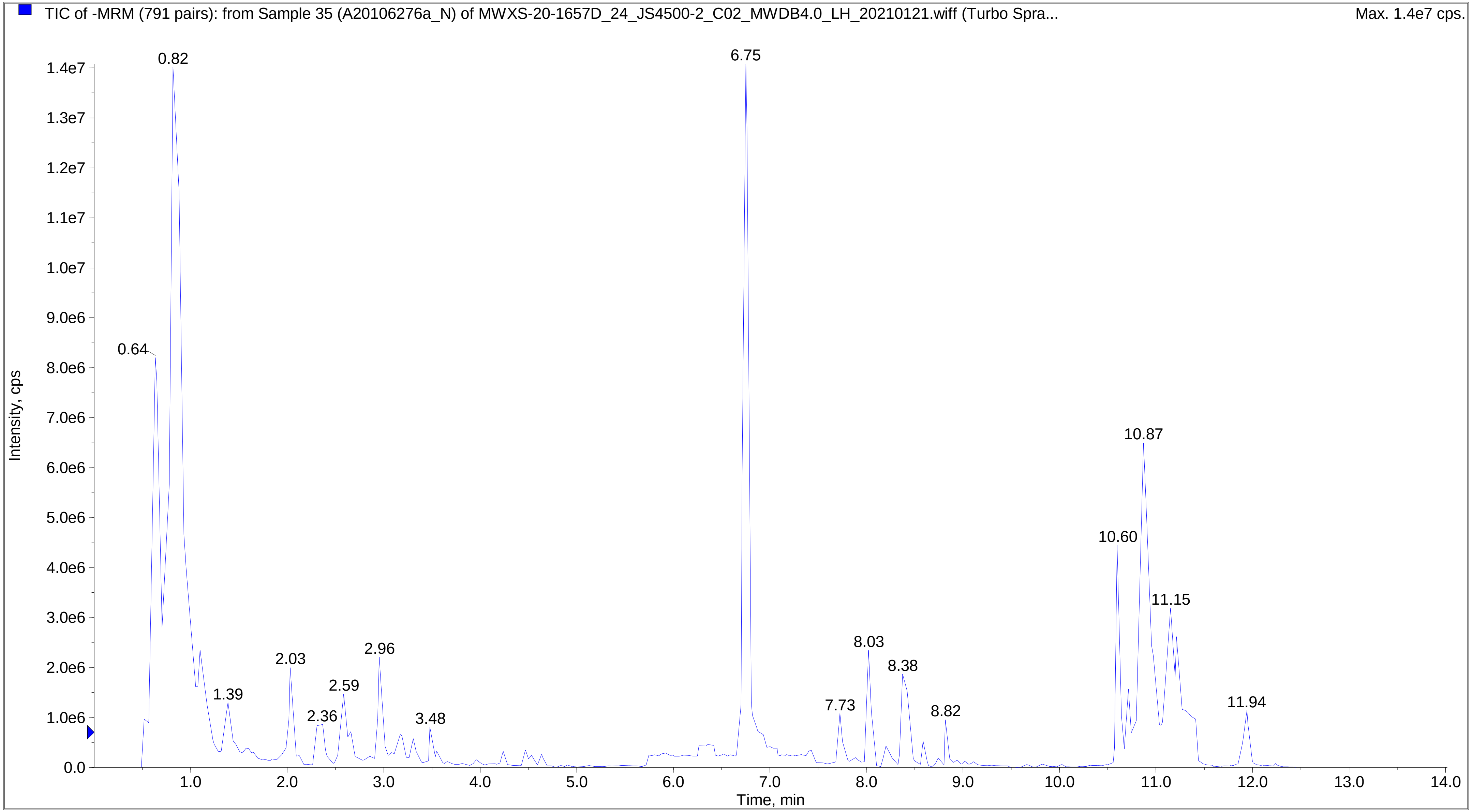
TIC of -MRM (791 pairs): from Sample 35 (A20106276a_N) of MWXS-20-1657D_24_JS4500-2_C02_MWDB4.0_LH_20210121.wiff (Turbo Spra...
Max. 1.4e7 cps.
Intensity, cps
Time, min
0.82
6.75
0.64
10.87
10.60
11.15
8.03
8.38
2.96
2.03
2.59
1.39
2.36
3.48
7.73
8.82
11.94
1.4e7
1.3e7
1.2e7
1.1e7
1.0e7
9.0e6
8.0e6
7.0e6
6.0e6
5.0e6
4.0e6
3.0e6
2.0e6
1.0e6
0.0
1.0
2.0
3.0
4.0
5.0
6.0
7.0
8.0
9.0
10.0
11.0
12.0
13.0
14.0

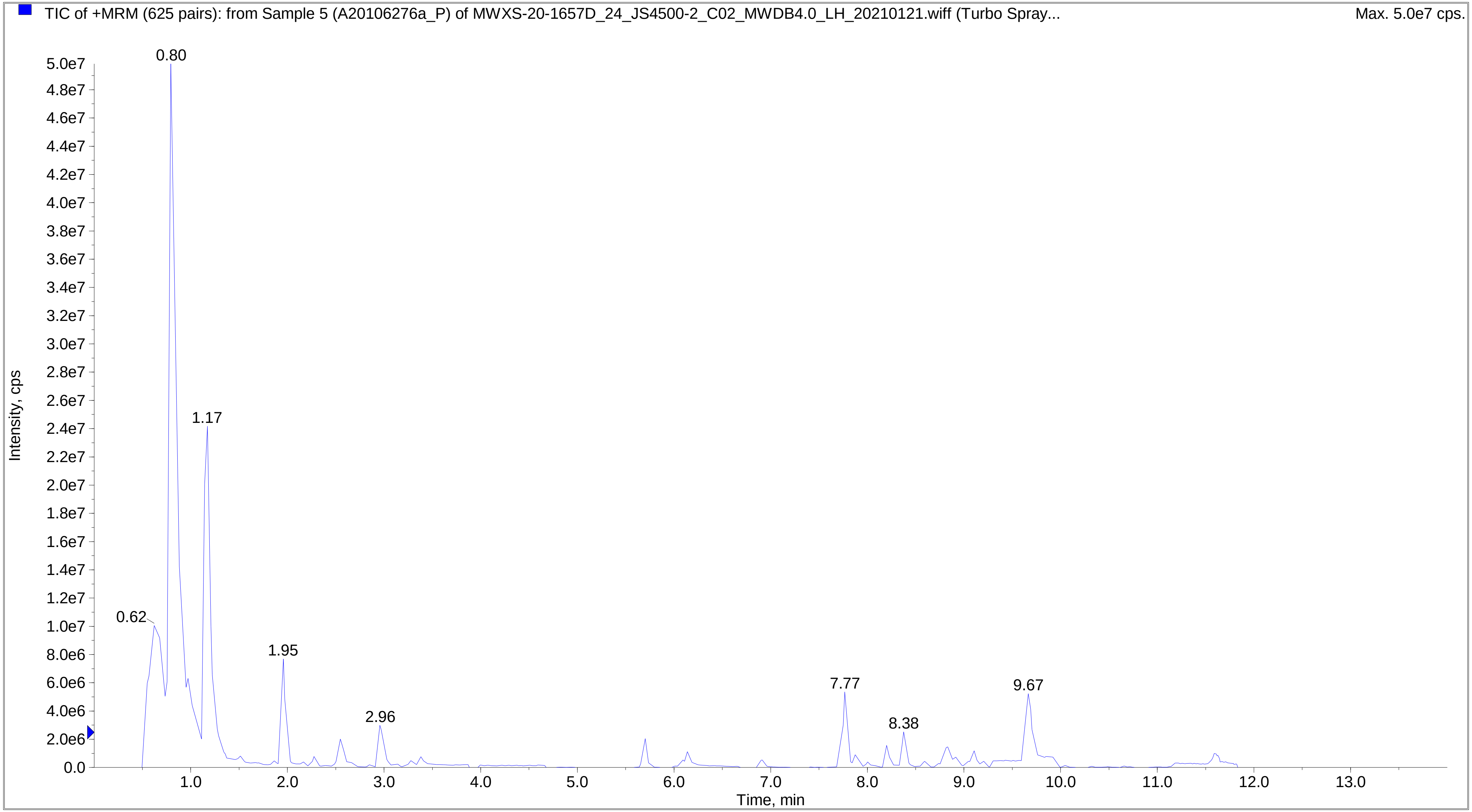
TIC of +MRM (625 pairs): from Sample 5 (A20106276a_P) of MWXS-20-1657D_24_JS4500-2_C02_MWDB4.0_LH_20210121.wiff (Turbo Spray...
Max. 5.0e7 cps.
Intensity, cps
Time, min
0.80
1.17
0.62
1.95
2.96
7.77
8.38
9.67

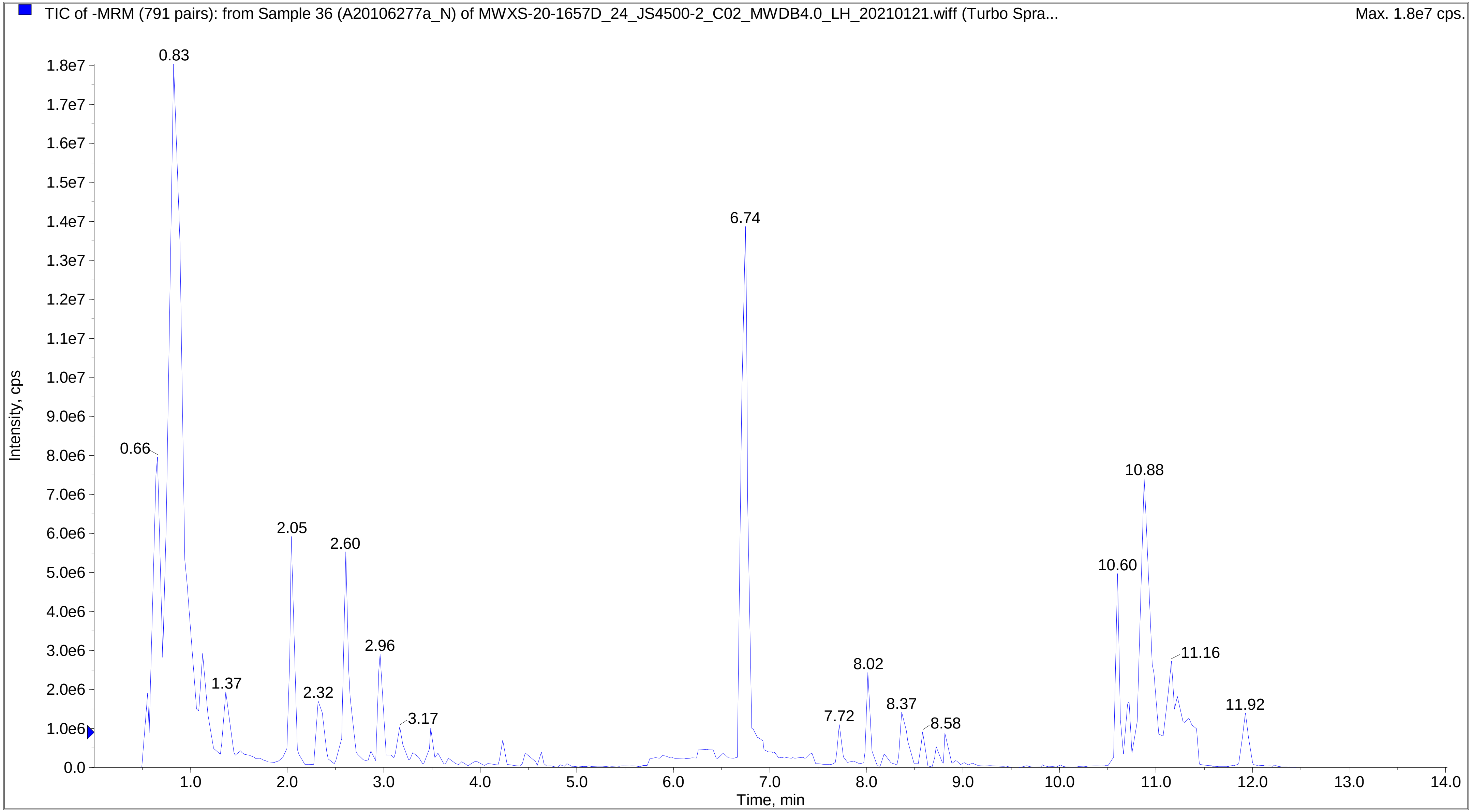

TIC of -MRM (791 pairs): from Sample 36 (A20106277a_N) of MWXS-20-1657D_24_JS4500-2_C02_MWDB4.0_LH_20210121.wiff (Turbo Spra...
Max. 1.8e7 cps.
Intensity, cps
Time, min
0.83
0.66
1.37
2.05
2.32
2.60
2.96
3.17
6.74
7.72
8.02
8.37
8.58
10.60
10.88
11.16
11.92

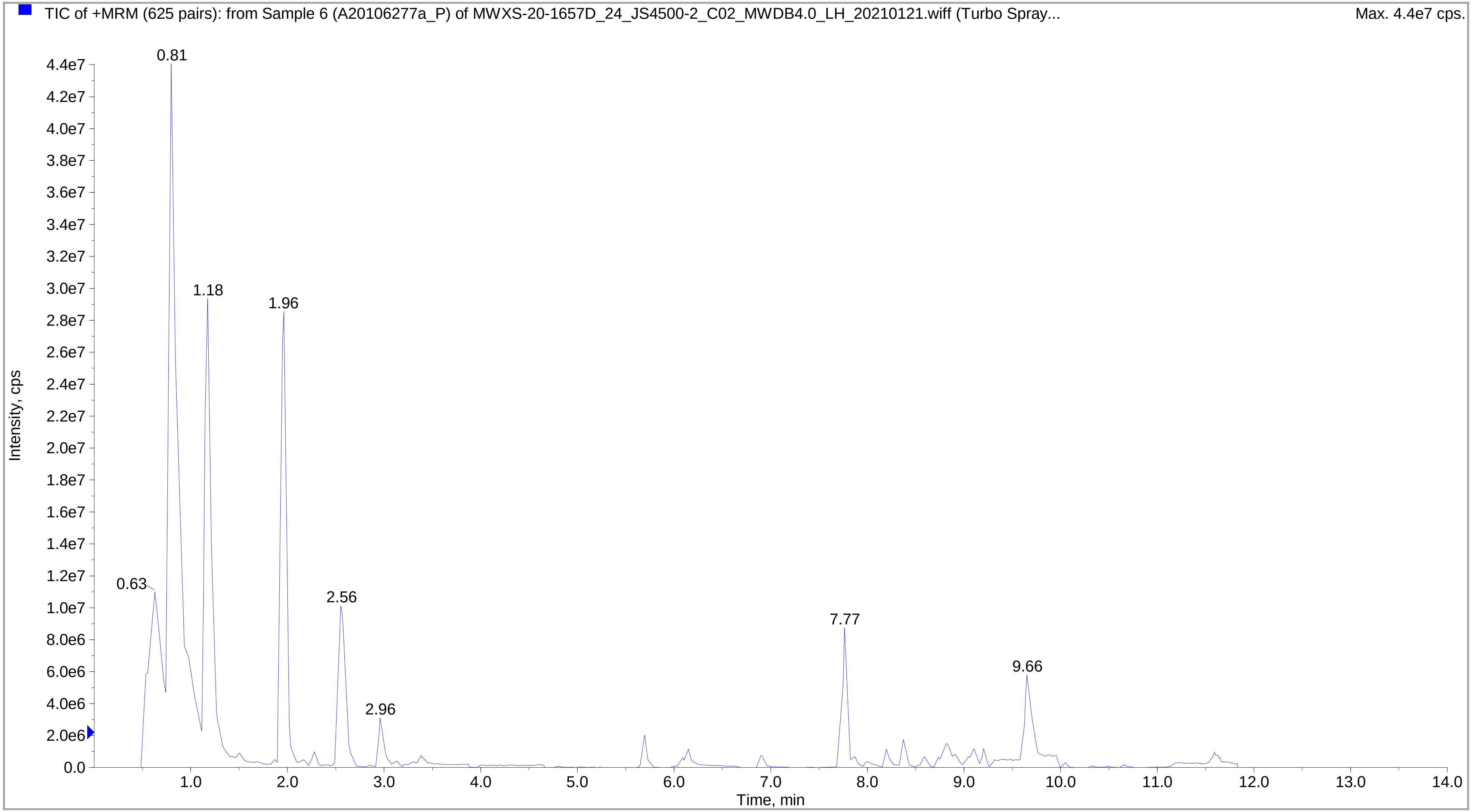

TIC of +MRM (625 pairs): from Sample 6 (A20106277a_P) of MWXS-20-1657D_24_JS4500-2_C02_MWDB4.0_LH_20210121.wiff (Turbo Spray...
Max. 4.4e7 cps.
Intensity, cps
Time, min
0.81
1.18
1.96
0.63
2.56
2.96
7.77
9.66
4.4e7
4.2e7
4.0e7
3.8e7
3.6e7
3.4e7
3.2e7
3.0e7
2.8e7
2.6e7
2.4e7
2.2e7
2.0e7
1.8e7
1.6e7
1.4e7
1.2e7
1.0e7
8.0e6
6.0e6
4.0e6
2.0e6
0.0
1.0
2.0
3.0
4.0
5.0
6.0
7.0
8.0
9.0
10.0
11.0
12.0
13.0
14.0

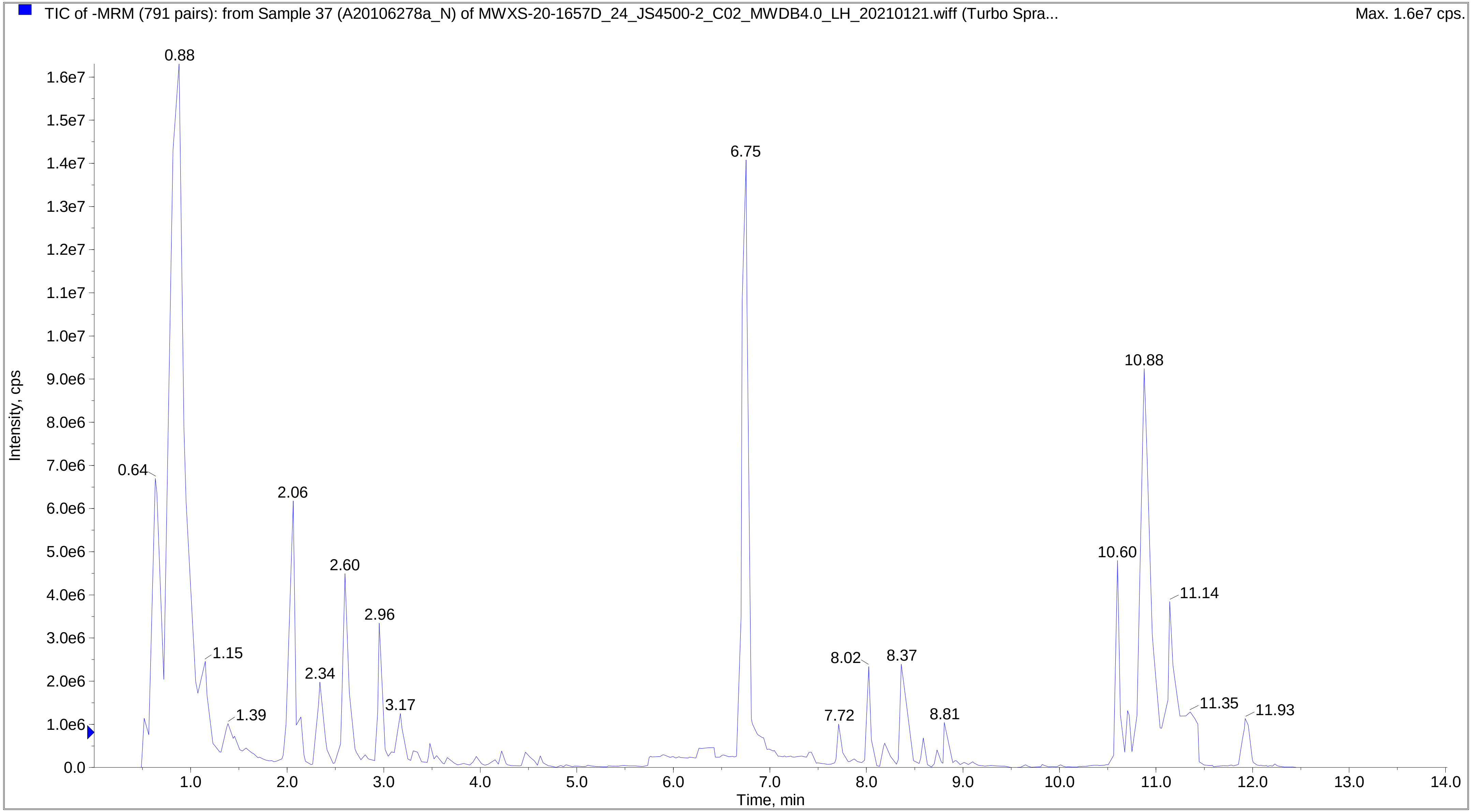

TIC of -MRM (791 pairs): from Sample 37 (A20106278a_N) of MWXS-20-1657D_24_JS4500-2_C02_MWDB4.0_LH_20210121.wiff (Turbo Spra...
Max. 1.6e7 cps.
Intensity, cps
Time, min
0.88
6.75
10.88
0.64
2.06
2.60
10.60
11.14
2.96
1.15
2.34
8.02
8.37
3.17
1.39
7.72
8.81
11.35
11.93

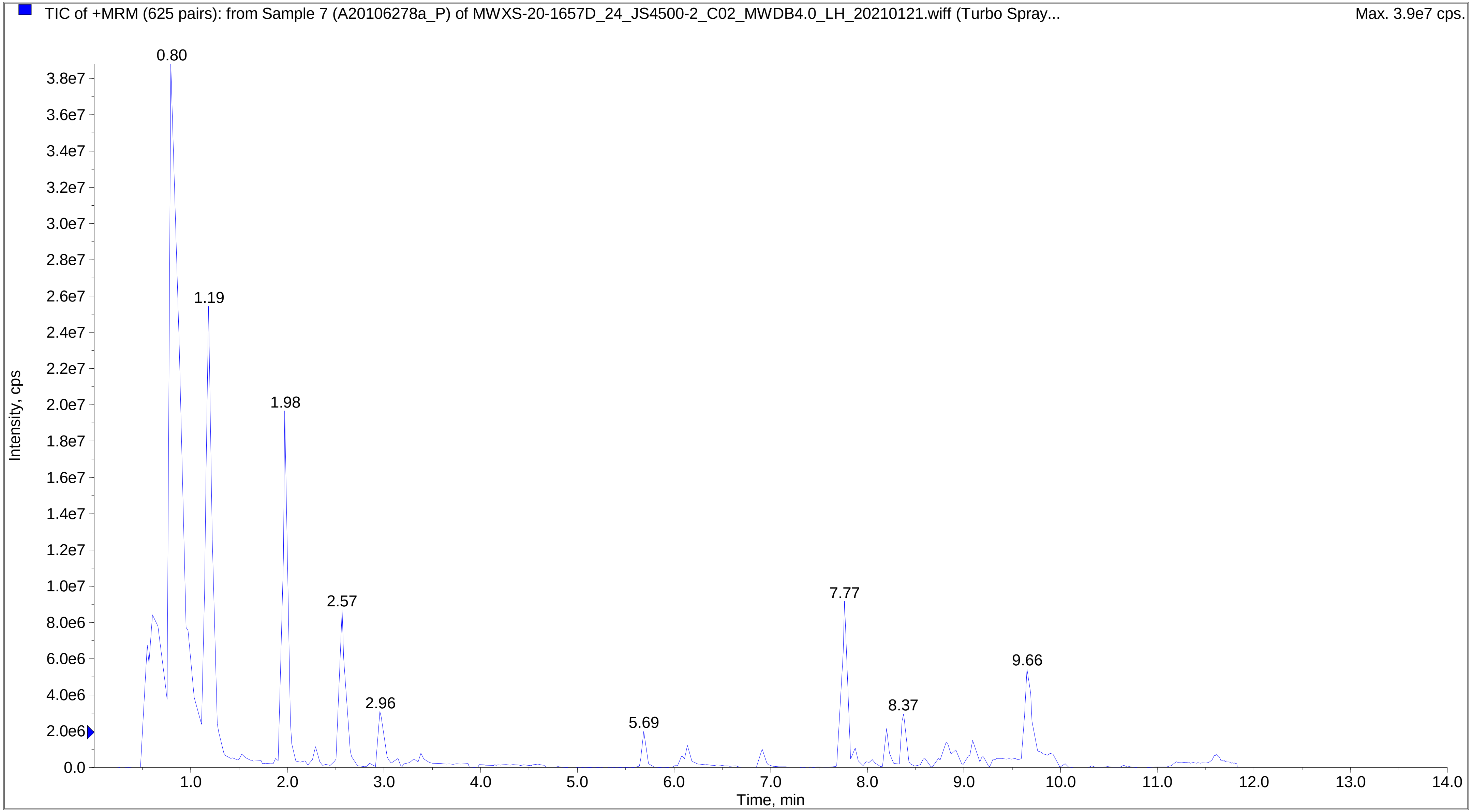

TIC of +MRM (625 pairs): from Sample 7 (A20106278a_P) of MWXS-20-1657D_24_JS4500-2_C02_MWDB4.0_LH_20210121.wiff (Turbo Spray...
Max. 3.9e7 cps.
0.80
1.19
1.98
2.57
2.96
5.69
7.77
8.37
9.66
Intensity, cps
Time, min

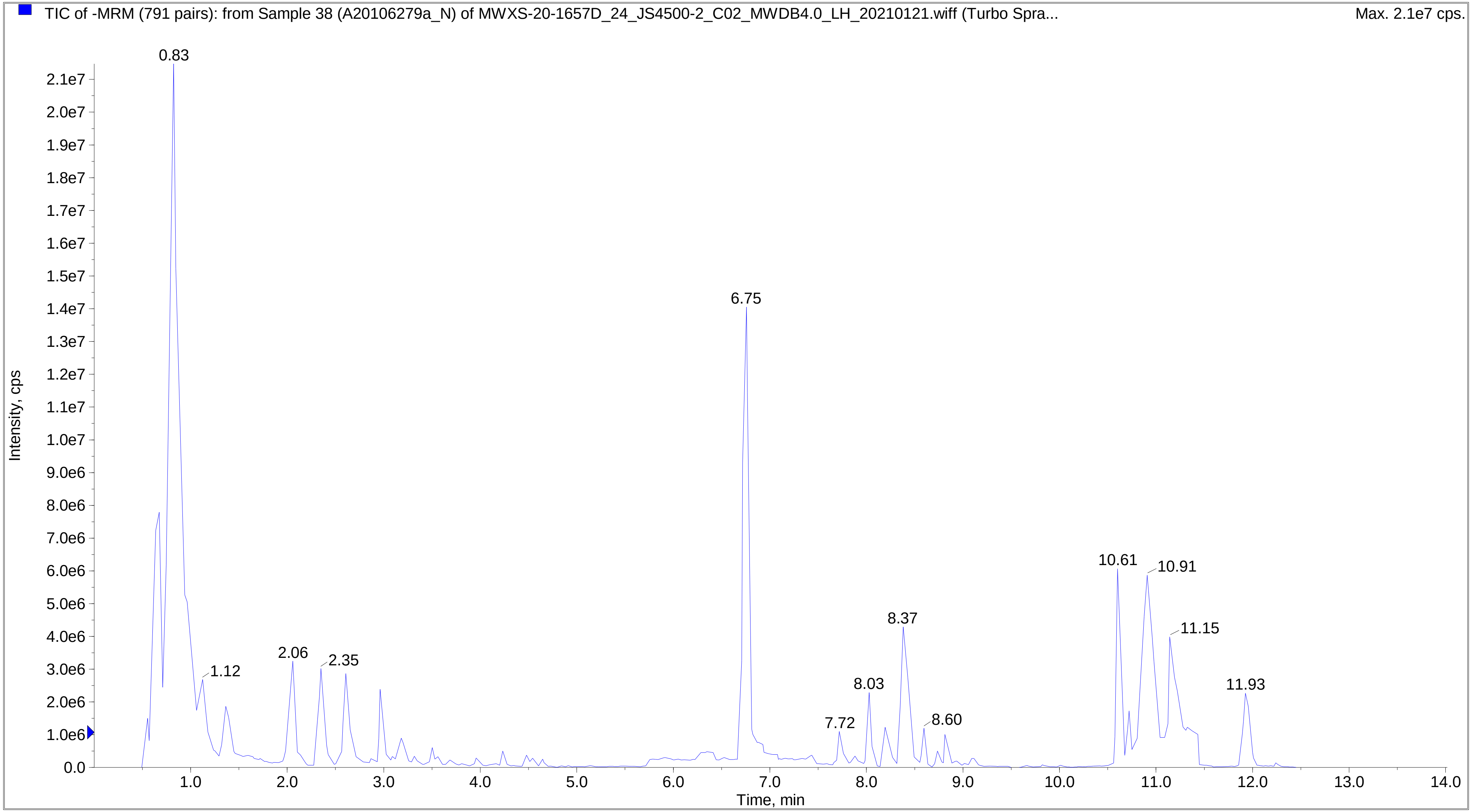

TIC of -MRM (791 pairs): from Sample 38 (A20106279a_N) of MWXS-20-1657D_24_JS4500-2_C02_MWDB4.0_LH_20210121.wiff (Turbo Spra...
Max. 2.1e7 cps.
Intensity, cps
Time, min
0.83
1.12
2.06
2.35
6.75
7.72
8.03
8.37
8.60
10.61
10.91
11.15
11.93

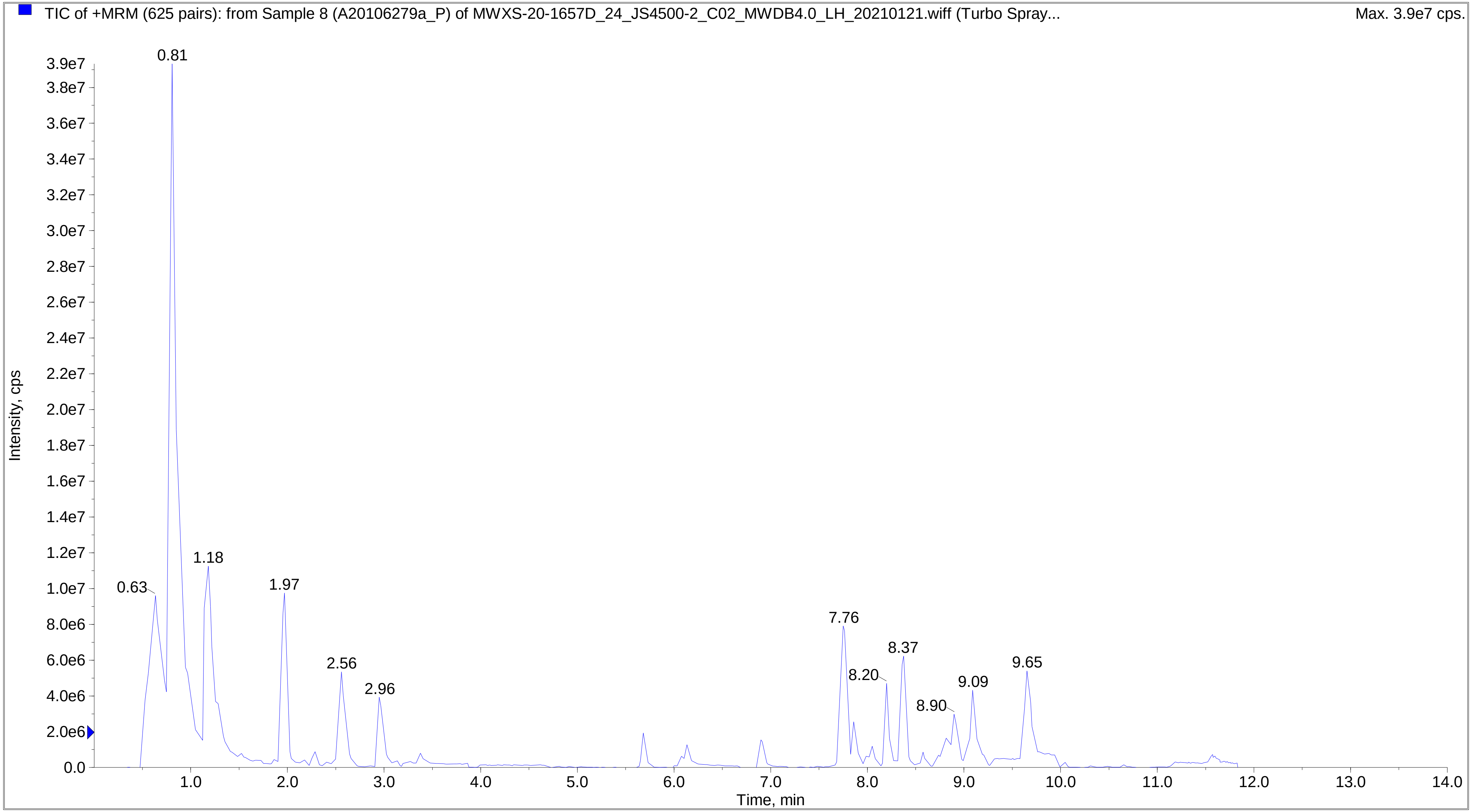
TIC of +MRM (625 pairs): from Sample 8 (A20106279a_P) of MWXS-20-1657D_24_JS4500-2_C02_MWDB4.0_LH_20210121.wiff (Turbo Spray...
Max. 3.9e7 cps.
Intensity, cps
Time, min
0.81
0.63
1.18
1.97
2.56
2.96
7.76
8.20
8.37
8.90
9.09
9.65

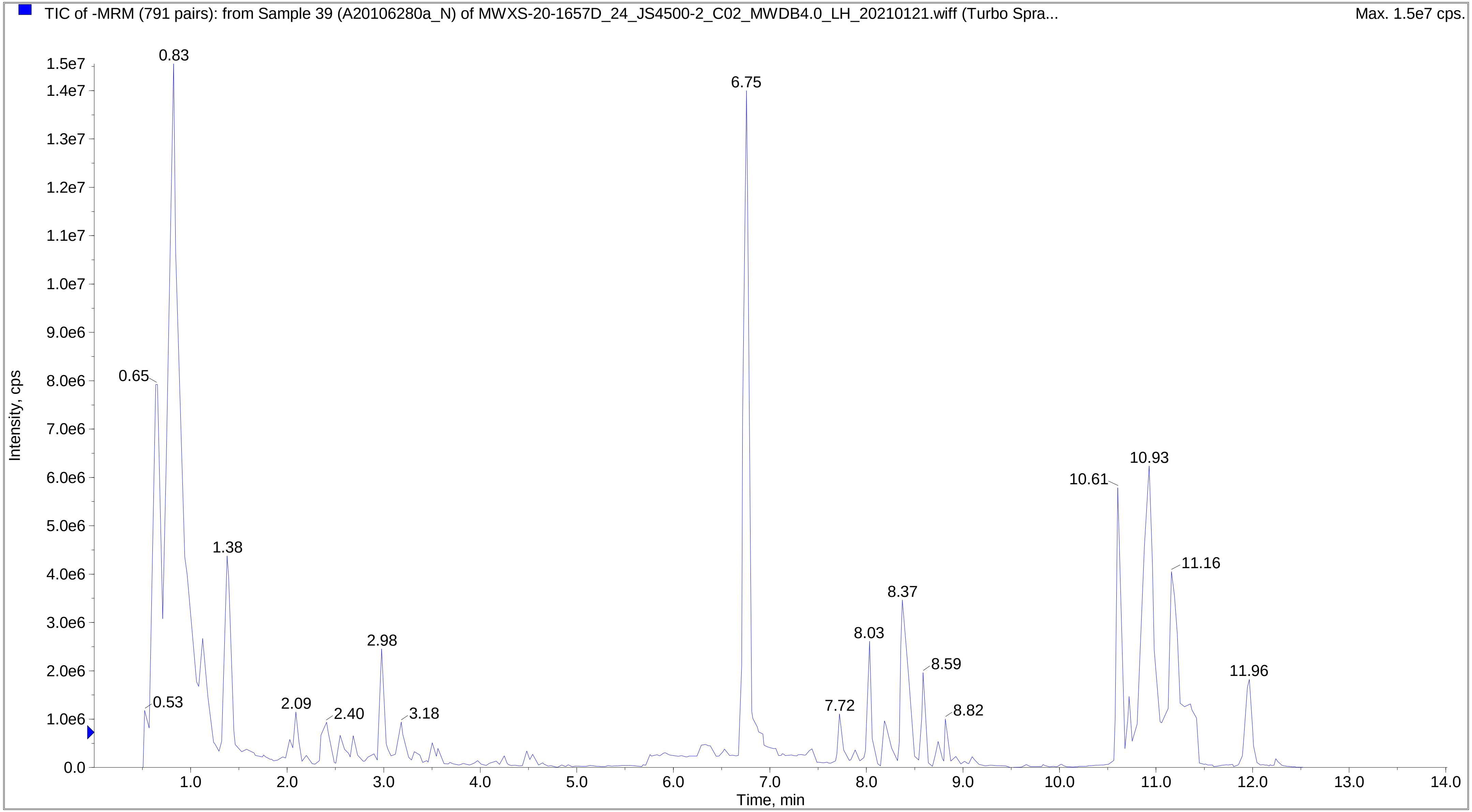

TIC of -MRM (791 pairs): from Sample 39 (A20106280a_N) of MWXS-20-1657D_24_JS4500-2_C02_MWDB4.0_LH_20210121.wiff (Turbo Spra...
Max. 1.5e7 cps.
Intensity, cps
Time, min
0.83
6.75
0.65
10.93
10.61
11.16
1.38
8.37
8.03
2.98
8.59
11.96
0.53
2.09
7.72
8.82
2.40
3.18
1.5e7
1.4e7
1.3e7
1.2e7
1.1e7
1.0e7
9.0e6
8.0e6
7.0e6
6.0e6
5.0e6
4.0e6
3.0e6
2.0e6
1.0e6
0.0
1.0
2.0
3.0
4.0
5.0
6.0
7.0
8.0
9.0
10.0
11.0
12.0
13.0
14.0

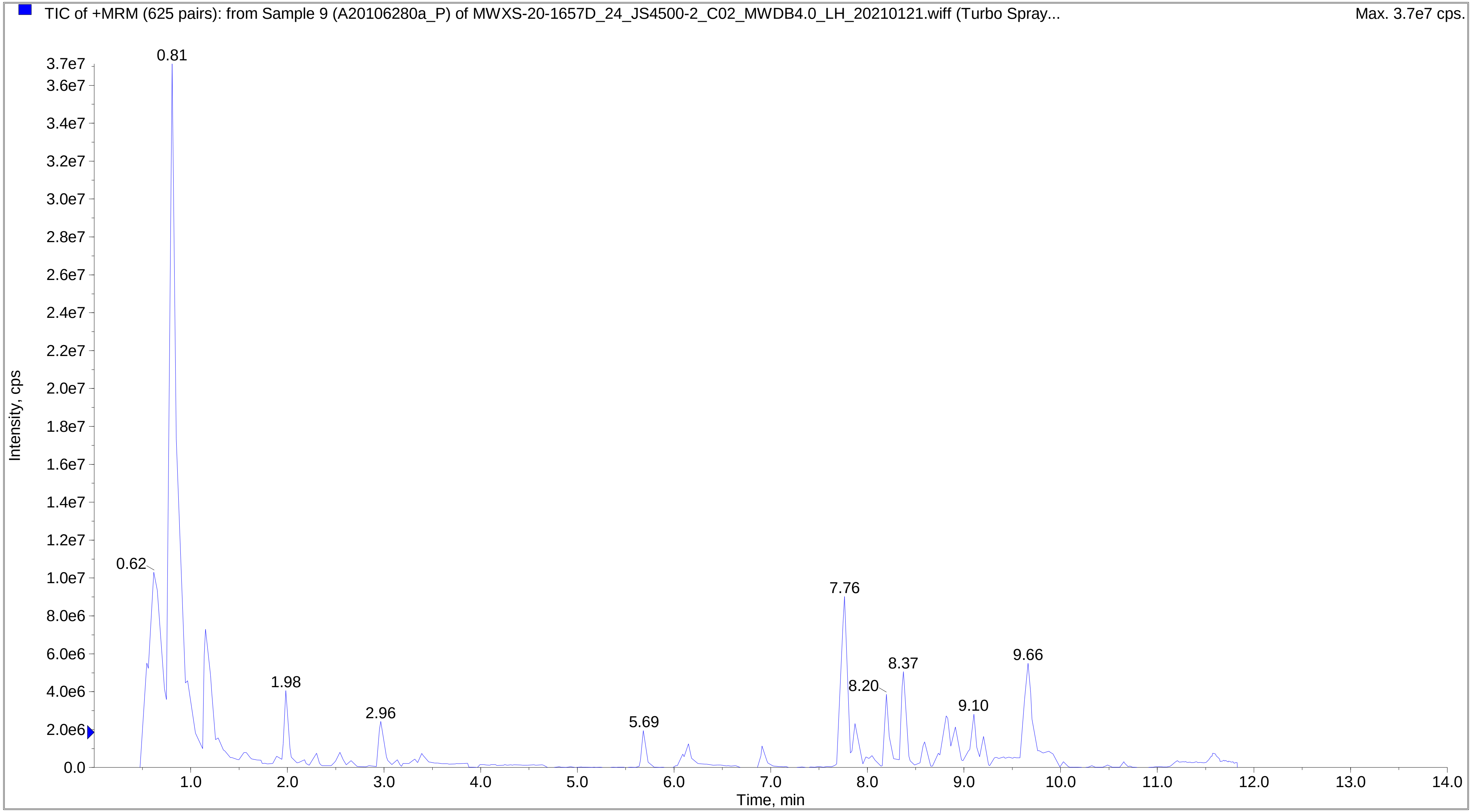
TIC of +MRM (625 pairs): from Sample 9 (A20106280a_P) of MWXS-20-1657D_24_JS4500-2_C02_MWDB4.0_LH_20210121.wiff (Turbo Spray...
Max. 3.7e7 cps.
Intensity, cps
Time, min
0.81
0.62
1.98
2.96
5.69
7.76
8.20
8.37
9.10
9.66

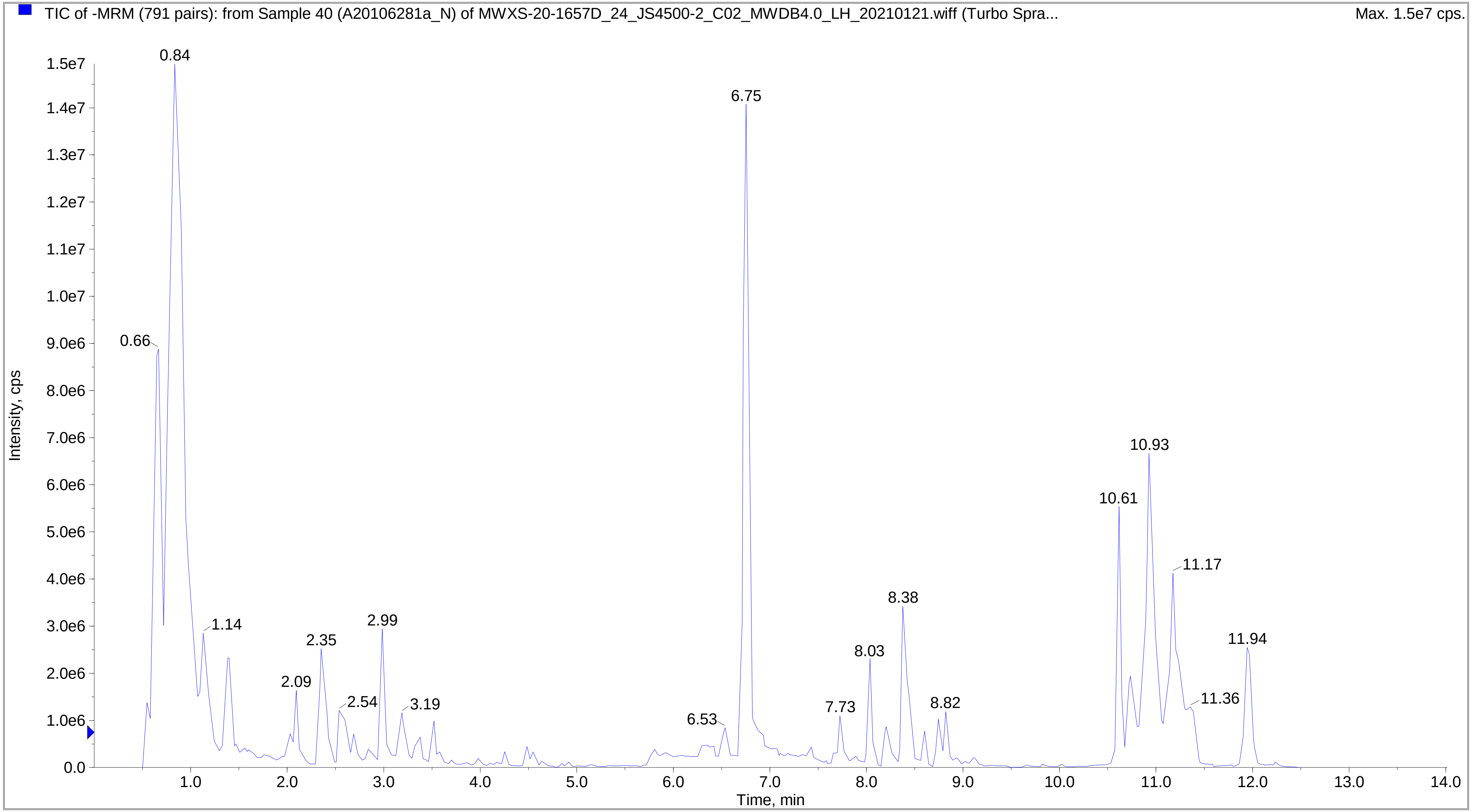

TIC of -MRM (791 pairs): from Sample 40 (A20106281a_N) of MWXS-20-1657D_24_JS4500-2_C02_MWDB4.0_LH_20210121.wiff (Turbo Spra...
Max. 1.5e7 cps.
Intensity, cps
Time, min
0.84
6.75
0.66
10.93
10.61
11.17
8.38
1.14
2.99
2.35
11.94
8.03
2.09
2.54
3.19
7.73
8.82
11.36
6.53
